# COVID-19 Surveillance in Madagascar and Urban Burkina Faso: Addressing Underreporting of Disease Burden Through Integrative Analysis of Diverse Data Streams

**DOI:** 10.1093/cid/ciaf041

**Published:** 2025-07-22

**Authors:** Njariharinjakamampionona Rakotozandrindrainy, Sophie S Y Kang, Lady R Wandji Nana, Jonathan D Sugimoto, Yi-Ting Wang, Ndrainaharimira Rakotozandrindrainy, Tsiriniaina Jean Luco Razafindrabe, Tiana Mirana Raminosoa, Sye Lim Hong, Mathilde Razafindrakalia, Gabriel Nyirenda, Tabea Binger, Ellen E Higginson, Hyon Jin Jeon, Namgay Wangmo, Gbènonminvo E Cakpo, YoungAe You, Birkneh Tilahun Tadesse, Abdramane Bassiahi Soura, Raphaël Rakotozandrindrainy, Florian Marks

**Affiliations:** Madagascar Institute for Vaccine Research, University of Antananarivo, Antananarivo, Madagascar; International Vaccine Institute, SNU Research Park, Seoul, Korea, Republic of Korea; Institut Supérieur des Sciences de la Population, Joseph KI-ZERBO University, Ouagadougou, Burkina Faso; International Vaccine Institute, SNU Research Park, Seoul, Korea, Republic of Korea; Department of Epidemiology, University of Washington, Seattle, Washington, USA; International Vaccine Institute, SNU Research Park, Seoul, Korea, Republic of Korea; Madagascar Institute for Vaccine Research, University of Antananarivo, Antananarivo, Madagascar; Madagascar Institute for Vaccine Research, University of Antananarivo, Antananarivo, Madagascar; Madagascar Institute for Vaccine Research, University of Antananarivo, Antananarivo, Madagascar; International Vaccine Institute, SNU Research Park, Seoul, Korea, Republic of Korea; Madagascar Institute for Vaccine Research, University of Antananarivo, Antananarivo, Madagascar; Madagascar Institute for Vaccine Research, University of Antananarivo, Antananarivo, Madagascar; International Vaccine Institute, SNU Research Park, Seoul, Korea, Republic of Korea; International Vaccine Institute, SNU Research Park, Seoul, Korea, Republic of Korea; Wellcome Sanger Institute, Wellcome Genome Campus, Hinxton, United Kingdom; Cambridge Institute of Therapeutic Immunology and Infectious Disease, University of Cambridge School of Clinical Medicine, Cambridge Biomedical Campus, Cambridge, United Kingdom; Madagascar Institute for Vaccine Research, University of Antananarivo, Antananarivo, Madagascar; International Vaccine Institute, SNU Research Park, Seoul, Korea, Republic of Korea; Cambridge Institute of Therapeutic Immunology and Infectious Disease, University of Cambridge School of Clinical Medicine, Cambridge Biomedical Campus, Cambridge, United Kingdom; International Vaccine Institute, SNU Research Park, Seoul, Korea, Republic of Korea; Institut Supérieur des Sciences de la Population, Joseph KI-ZERBO University, Ouagadougou, Burkina Faso; International Vaccine Institute, SNU Research Park, Seoul, Korea, Republic of Korea; International Vaccine Institute, SNU Research Park, Seoul, Korea, Republic of Korea; Heidelberg Institute of Global Health, University of Heidelberg, Heidelberg, Germany; Division of Clinical Pharmacology, Department of Global Public Health, Karolinska Institutet, Stockholm, Sweden; Institut Supérieur des Sciences de la Population, Joseph KI-ZERBO University, Ouagadougou, Burkina Faso; Madagascar Institute for Vaccine Research, University of Antananarivo, Antananarivo, Madagascar; Madagascar Institute for Vaccine Research, University of Antananarivo, Antananarivo, Madagascar; International Vaccine Institute, SNU Research Park, Seoul, Korea, Republic of Korea; Cambridge Institute of Therapeutic Immunology and Infectious Disease, University of Cambridge School of Clinical Medicine, Cambridge Biomedical Campus, Cambridge, United Kingdom; Heidelberg Institute of Global Health, University of Heidelberg, Heidelberg, Germany; The Hong Kong Jockey Club Global Health Institute, Hong Kong Special Administrative Region, China

**Keywords:** COVID-19, serosurveillance, Madagascar, Burkina Faso

## Abstract

**Background:**

Coronavirus disease 2019 (COVID-19) caused substantial disease and death worldwide since December 2019, but the burden was lower in Africa than in high-income countries. To address potential underreporting, we modeled severe acute respiratory syndrome coronavirus 2 (SARS-CoV-2) infection and disease burden in Burkina Faso and Madagascar.

**Methods:**

Prospectively enrolled patients who presented with fever at sentinel healthcare facilities were assessed for active SARS-CoV-2 infection. Household members of SARS-CoV-2–infected patients were prospectively followed for confirmed SARS-CoV-2 infection. Archived serum specimens that spanned the pandemic onset in Madagascar to the start of prospective surveillance were tested for anti–SARS-CoV-2 immunoglobulins. Data from these multiple sources contributed to an integrated analysis to calibrate an epidemiologic mass action model.

**Results:**

COVID-19 accounted for a substantial fraction of healthcare-ascertained febrile illness in both Burkina Faso and Madagascar, with symptom profiles consistent with those previously reported. SARS-CoV-2 vaccination coverage was very low in Burkina Faso and unavailable in Madagascar. The household secondary attack rate was 28% (95% confidence intervals [CI], 22%–35%] in Madagascar and 31% (95% CI: 9%–68%) in Burkina Faso, indicating substantial transmission of the disease within households in both locations. Model simulations estimated that the actual number of SARS-CoV-2 infections was at least nine times higher than the reported number of febrile COVID-19 cases.

**Conclusions:**

Africa has faced persistent challenges due to underinvestment in vaccination programs and disease surveillance programs. There was substantial underreporting of COVID-19 cases during the pandemic in both countries. Our findings call for improving systems and resources in disease surveillance during epidemic and interepidemic periods in these countries.

## Teaser

Africa's underinvestment in vaccination and disease surveillance led to significant COVID-19 underreporting. Our findings highlight the need for improved systems and resources for disease surveillance during both epidemic and interepidemic periods in Burkina Faso and Madagascar.

Four years have passed since the coronavirus disease 2019 (COVID-19) pandemic was declared in March 2020. This crisis has substantially affected people's livelihoods worldwide. Multiple waves have been driven by various severe acute respiratory syndrome coronavirus 2 (SARS-CoV-2) variants of concern, including Alpha, Beta, Delta, and Omicron. As of 20 October 2024, more than seven million people had died as a result of COVID-19 [1, 2], and all countries worldwide were affected. Although high-throughput testing was conducted in the global north, the response in the African region has differed. Of these global totals, 9 582 835 cases and 175 531 fatalities were reported for the Africa Region, suggesting a lower cumulative impact of SARS-CoV-2 than observed in higher-income settings [2]. Yet, the pandemic has profoundly disrupted socioeconomic aspects of life.

Early in the pandemic, manufacturers quickly developed rapid, inexpensive, globally available diagnostic tools for SARS-CoV-2 infection, many of which could provide results *in situ* within 10–30 minutes. The World Health Organization (WHO) supported the use of a low-cost, high-quality COVID-19 antigen rapid test in low- and middle-income countries [3], that is, SD Biosensor (Standard Diagnostics, Korea).

Countries across the globe implemented measures to control the spread of SARS-CoV-2, relying on prior experience with pandemic infections. Some nations, including African countries that initially reported few cases, used stringent restrictions to control excess mortality. Madagascar and Burkina Faso, for example, initiated national lockdowns in 2020 [4–7].

Madagascar, with a population that exceeds 25 million people, reported more than 68 572 polymerase chain reaction (PCR)–confirmed cases that resulted in 1428 deaths as of September 2024 [2]. Burkina Faso has a population of 23 703 214 people [8] and reported 22 139 PCR-confirmed cases and 400 deaths [2] as of September 2024. Most confirmed cases were concentrated around the capitals of each country. Despite facing measles and plague epidemics, the reported SARS-CoV-2 infection rates for Madagascar remained relatively low. For both countries, low testing rates underscore the need for more reliable data, but underfunding, high patient-to-physician ratios [9], and poor access to primary healthcare services [10] are significant hurdles to improving testing rates for infectious diseases.

To assess the level of potential underreporting of symptomatic SARS-CoV-2 infection that requires healthcare, existing healthcare facility–based surveillance systems for febrile illness in Madagascar and Burkina Faso were augmented to test for SARS-CoV-2 infection. In both countries, prospectively collected specimens were tested for evidence of SARS-CoV-2 infection using real-time reverse-transcription PCR (rRT-PCR), a rapid antigen test, and/or serologic assays. Additionally, Madagascar also investigated retrospectively collected samples [11]. Since transmission of SARS-CoV-2 to the household contacts of confirmed COVID-19 cases is estimated to be 10 times higher than for nonhousehold contacts [12, 13], the risk of infection among the contacts of prospectively ascertained, rRT-PCR–confirmed symptomatic SARS-CoV-2 cases was also evaluated in Madagascar and Burkina Faso. All available study data for each country were integrated analytically to estimate the weekly burden of SARS-CoV-2 infection that required healthcare from the date of the first reported COVID-19 case in each country [14] until the end of the study's prospective surveillance.

## METHODS

### Study Areas

The activities in Madagascar were led by the Madagascar Institute for Vaccine Research (MIVR) and supported by the International Vaccine Institute (IVI). Three areas of Madagascar were studied: a semi-urban site (Imerintsiatosika) and two rural areas (Ampefy, 120 km west of Antananarivo, and Andina, 270 km south of Antananarivo). In Imerintsiatosika, surveillance was performed at the Centre de Santé Base-II (CSB-II), the main health center that recruited people with suspected COVID-19. The catchment areas included Imerintsiatosika and adjacent regions, covering a population of approximately 150 000 people and aligning with the Severe Typhoid Fever in Africa Program (SETA) study catchment area [11]. Further west, recruitment was performed at the CSB-II in Ampefy. Although Ampefy's population is approximately 20 000, the CSB-II covers a larger surveillance area of approximately 50 000 people who originate from adjacent areas. Finally, in the very remote rural site of Andina, recruitment was performed at the Centre Communautaire de Santé, which serves approximately 70 000 people.

In Burkina Faso, activities were conducted in adjacent neighborhoods in urban Ouagadougou [11, 15, 16]. The IVI and the Joseph KI-ZERBO University/Institut Supérieur des Sciences de la Population (ISSP) have supported the surveillance for febrile illness in this population since 2011, covering a population of approximately 25 000 [15, 16].

As an augment to existing surveillance efforts, the IVI has provided participating healthcare facilities in Madagascar and Burkina Faso with appropriate test kits to improve understanding of the local course of the COVID-19 pandemic. Various activities were conducted in collaboration with national authorities to enhance screening and diagnostic capabilities. Both rRT-PCR and rapid antigen tests were used for diagnosing COVID-19, thereby enabling a comparison of rapid antigen test performance against the gold standard, PCR.

### Study Population

Prospective surveillance and the household study were conducted from 24 May 2021 until 17 June 2023 in Madagascar and from 15 June 2021 until 19 December 2022 in Burkina Faso. The surveillance sites in Madagascar and Burkina Faso serve estimated catchment populations of 220 000 and 80 000 people, respectively. Febrile illness surveillance for *Salmonella* Typhi [16] has been conducted at the Madagascar site for more than ten years, and historical serum specimens were available from 14 December 2018 onward for rapid antibody testing.

### Inclusion and Exclusion Criteria

At all sites in Madagascar and Burkina Faso, we installed dedicated COVID-19 enrollment booths outside the healthcare centers to ensure social distancing. Healthcare workers who were provided with essential personal protective equipment comprising masks and hand sanitizer screened patients at the entry doors for symptoms such as fever, recent symptoms, and potential recent exposure. Arriving febrile patients were directed to a designated waiting area (outside) and given face masks. The COVID-19 screening criteria involved identification of suspected cases who presented with influenza-like illness and/or respiratory symptoms or who self-reported recent exposure to COVID-19. Screening tests were conducted in a dedicated room away from other patients. Individuals with suspected SARS-CoV-2 infection received information on necessary distancing and quarantine procedures, face masks, and sanitizer. Patients with COVID-19 signs and symptoms and those who reported recent exposure to the virus were invited to enroll. Informed consent forms were signed.

### Prospective Surveillance Specimen Collection

At all surveillance sites, study staff members collected oropharyngeal and nasopharyngeal swab samples for SARS-CoV-2 detection in nasal and/or oral mucosa. Customized training courses for healthcare staff were initiated before recruitment and repeated periodically to ensure appropriate sample collection. Samples for quantitative PCR (qPCR) testing were stored locally at between 2°C and 8°C and transported daily to the reference laboratories. In Madagascar, they were sent to the University of Antananarivo (UoA)/MIVR laboratory; in Burkina Faso, they were sent to the laboratory of the Schiphra Protestant Hospital. These samples included serum samples taken as part of the ongoing SETA study [11]. Completed recruitment forms were sent to UoA/MIVR for Madagascar and ISSP/Université Joseph KI-ZERBO for Burkina Faso.

### Household Transmission Study

Surveillance participants who tested positive for SARS-CoV-2 at enrollment were invited to participate in a household transmission study. The household members of surveillance participants (household index cases) were asked to provide informed consent to participate in the household transmission study. Household members were defined as individuals who reported staying at least three nights in the same residence as the index case. At enrollment, consenting household members and their index case provided blood samples for rapid antibody testing and completed a questionnaire regarding COVID-19 risk factors, vaccination history, and demographic information. Household members and their index case were asked to contact study staff and/or seek care at a healthcare facility that was participating in the surveillance program if they experienced febrile illness (≥37.5°C) and cough that lasted three days or self-reported three or more of the following signs or symptoms in the past ten days: fever (≥37.5°C), cough, general weakness/fatigue, headache, myalgia, sore throat, coryza, dyspnea, anorexia/nausea/vomiting, diarrhea, altered mental status, or loss of smell (anosmia) and/or taste (ageusia). Every seven days after enrollment, enrolled households were contacted in person to determine whether any member had experienced febrile illness since the previous study contact. Contact (excluding the index case) continued until there were no susceptible household members in the household or there were no new confirmed cases by rapid antigen test or qPCR over 14 days since the last confirmed case. All available members underwent rapid serum antibody testing at the final household visit. Oral/nasal mucosal swab samples were collected from household transmission study participants who reported febrile illness, stored, transported, and assayed using qPCR in the same manner as swabs collected from surveillance participants.

### Archived Specimens From Madagascar

The MIVR and IVI maintain a biorepository of SETA serum specimens collected since 2014. After appropriate ethical review and approval, anonymized specimens from 2018 to the early 2020s were made available when the first confirmed COVID-19 case was reported in Madagascar [14]. These specimens were rapidly tested in situ for evidence of the presence or absence of anti–SARS-CoV-2 immunoglobulin (Ig) G and/or IgM. A random subset of specimens was also assayed with a more sensitive enzyme-linked immunosorbent assay (ELISA) for anti–SARS-CoV-2 IgG.

### Specimen Testing During the Prospective Surveillance

Nasal and oral mucosal swab specimens collected for prospective disease surveillance and the household transmission studies in Burkina Faso and at the CSB-II at the Imerintsiatosika and Ampefy sites in Madagascar were tested for SARS-CoV-2 RNA via qPCR. The MIVR has a dedicated laboratory with real-time qPCR capability (Mic qPCR cycler, Biomolecular Systems, Coomera, Australia) for COVID-19 diagnostics [17]. The laboratory followed established standard operating procedures and routine quality control checks conducted through physical visits or virtual audits. Swab specimens from all sites were tested with rapid STANDARD Q COVID-19 Ag Tests (SD Biosensor, Korea), which has a reported sensitivity of 84.97% and specificity of 98.94% [18]. Blood samples collected via fingerpick were used in the household transmission study, and archived SETA specimens in Madagascar were tested for anti–SARS-CoV-2 IgG and IgM presence/absence with rapid STANDARD Q COVID-19 Ab Tests (SD Biosensor, Korea). A random subset of archived SETA specimens was also tested via a more sensitive and specific anti–SARS-CoV-2 IgG ELISA (WANTAI) that targeted the Spike domain [19].

### Statistical Analyses

For Madagascar, only enrollment dates and rapid antibody test results were available for retrospective samples, whereas prospective surveillance captured detailed data on symptoms, test results, treatments, and risk factors. In Burkina Faso, although SARS-CoV-2 vaccination data were collected and no such data were collected for Madagascar, they were excluded from analysis because of low vaccination coverage. The household study focused on estimating secondary attack rates and infection probabilities from symptomatic and asymptomatic SARS-CoV-2 infection data from rapid antigen, antibody, and rRT-PCR tests. Further details are provided in the Supplementary Material (Household Study Analysis section).

### Analytical Methods

The burden of SARS-CoV-2 infection was estimated through a two stage integrated analysis of the retrospective and prospective disease surveillance and household transmission study data. First, a chain-binomial model [20] estimated the household secondary attack rate and related parameters that characterize the risk of SARS-CoV-2 infection among household contacts and account for missing immunity status and the role of exposure to external infection. Next, parameter estimates from the first stage of the analysis were incorporated into the calibration of a susceptible, infectious, and recovered (SIR) model modified to track cumulative exposure to SARS-CoV-2 infection. Using a Poisson measurement model, the modified SIR was calibrated to the number of prospectively ascertained rapid antigen test or rRT-PCR–positive symptomatic SARS-CoV-2 surveillance cases and, for the Madagascar model only, the expected number of anti–SARS-CoV-2 IgG-positive members of the population, given the results of the rapid antibody test of the retrospective surveillance specimens. Model calibration was conducted using a modified iterated filtering algorithm (*mif2*) implemented in the *pomp* library for the R (v4.2.3) statistical computing environment [21]. The calibrated model for each country was used to simulate the expected weekly number of laboratory-confirmed symptomatic SARS-CoV-2 infections detected by the study's surveillance system and the weekly number of SARS-CoV-2 infections expected to have occurred among members of the system's catchment population. Additional details on methods and model inputs are provided in the Supplementary Material (Household Data Analysis and Surveillance Data Analysis sections).

### Ethics

This study was approved by the IVI Institutional Review Board (IRB), the Ministry of Health/Agence de Medicament/Comité d’Ethique de la Recherche Biomédicale in Madagascar, and the Ministry of Health/Health Research Ethics Committee in Burkina Faso. The use of archived SETA specimens was approved by the IVI IRB and the Malagasy Ethical Review Board. During and after the program (until the primary objectives of the study were reported), COVID-19 cases were reported to the Malagasy and Burkina Faso authorities. All other data on participants were strictly confidential and were not revealed to any third party by any member of the study team. All necessary precautions were taken to ensure that participants' rights, privacy, and freedom were respected. Each participant was informed of the study objective and methods and freely provided signed informed consent before participating.

## RESULTS

### Retrospective Surveillance Specimens From Madagascar

A total of 50–100 specimens from 1- to 3-month periods from 14 December 2018 underwent rapid antibody testing for the presence/absence of anti–SARS-CoV-2 IgG and IgM (Table 1*A*). The prevalence of seropositivity to SARS-CoV-2 IgG and/or IgM remained less than 5% until early March 2020, coinciding with report of the first cases of SARS-CoV-2 in Madagascar [22, 23]. The seroprevalence for ELISA-detected IgG was 0.2% between 14 December 2018 and 2 March 2020. After 3 March 2020, the observed seroprevalence for anti–SARS-CoV-2 IgG detected by rapid antibody test ranged from 4% to 43% and, with the exception of 3 March 2020 to 5 June 2020, was lower than the corresponding estimate that relied on ELISA-based detection, which ranged from 36% to 94%.

**Table 1.** Description of the Study Populations From Madagascar and Burkina Faso

Retrospective Surveillance Specimens From Madagascar							
Time Period		Number of Specimens	Number (%) of Specimens Positive by Rapid Antibody Test			Number (%) of Specimens in Validation Set	Number (%) of Specimens in the Validation Set Positive for SARS-CoV-2 IgG by Enzyme-Linked Immunosorbent Assay
Start	End		IgG-Positive Only	IgM-Positive Only	IgM- and IgG-Positive		
12/14/2018	1/21/2019	80	1 (1)	1 (1)	0 (0)	9 (11)	0 (0)
1/22/2019	2/19/2019	81	0 (0)	0 (0)	0 (0)	3 (4)	0 (0)
2/20/2019	4/4/2019	81	1 (1)	3 (4)	0 (0)	8 (10)	0 (0)
4/5/2019	6/2/2019	81	1 (1)	0 (0)	0 (0)	11 (14)	0 (0)
6/3/2019	7/25/2019	80	0 (0)	3 (4)	0 (0)	7 (9)	0 (0)
7/26/2019	10/9/2019	81	2 (2)	0 (0)	0 (0)	12 (15)	1 (8)
10/12/2019	12/12/2019	81	0 (0)	0 (0)	0 (0)	4 (5)	0 (0)
1/1/2020	1/31/2020	48	1 (2)	0 (0)	0 (0)	5 (10)	0 (0)
2/3/2020	3/2/2020	52	1 (2)	0 (0)	0 (0)	3 (6)	0 (0)
3/3/2020	6/5/2020	100	2 (2)	3 (3)	2 (2)	19 (19)	0 (0)
6/6/2020	10/5/2020	100	9 (9)	2 (2)	9 (9)	31 (31)	15 (48)
10/6/2020	12/4/2020	100	10 (10)	4 (4)	3 (3)	24 (24)	12 (50)
12/5/2020	2/4/2021	81	4 (5)	1 (1)	4 (5)	25 (31)	9 (36)
2/5/2021	4/15/2021	100	21 (21)	0 (0)	6 (6)	37 (37)	27 (73)
4/16/2021	7/5/2021	100	31 (31)	3 (3)	8 (8)	53 (53)	35 (66)
7/6/2021	7/22/2021	70	28 (40)	1 (1)	2 (3)	31 (44)	29 (94)

Prospective Surveillance and Household Contacts of SARS-CoV-2–Positive Index Cases From Madagascar, Frequency (%) by Characteristic					
Characteristic	Prospective Surveillance by SARS-CoV-2 RT-PCR and/or Rapid Antigen Test			Household Contacts	All Prospectively Enrolled Participants
	Negative	Positive			
		Not Enrolled in Household Study	Enrolled in Household Study		
N	1129	271	232	596	2228
Enrollment year–Month					
2021–5	2 (0)	1 (0)	0 (0)	0 (0)	3 (0)
2021–6	14 (1)	3 (1)	0 (0)	0 (0)	17 (1)
2021–7	17 (2)	3 (1)	0 (0)	0 (0)	20 (1)
2021–8	12 (1)	0 (0)	0 (0)	0 (0)	12 (1)
2021–9	38 (3)	0 (0)	1 (0)	0 (0)	39 (2)
2021–10	47 (4)	1 (0)	2 (1)	0 (0)	50 (2)
2021–11	58 (5)	11 (4)	7 (3)	34 (6)	110 (5)
2021–12	178 (16)	101 (37)	49 (21)	147 (25)	475 (21)
2022–1	150 (13)	87 (32)	87 (38)	173 (29)	497 (22)
2022–2	81 (7)	28 (10)	33 (14)	113 (19)	255 (11)
2022–3	88 (8)	6 (2)	6 (3)	17 (3)	117 (5)
2022–4	57 (5)	3 (1)	1 (0)	1 (0)	62 (3)
2022–5	48 (4)	2 (1)	1 (0)	3 (1)	54 (2)
2022–6	47 (4)	13 (5)	25 (11)	44 (7)	129 (6)
2022–7	46 (4)	3 (1)	8 (3)	28 (5)	85 (4)
2022–8	51 (5)	0 (0)	0 (0)	8 (1)	59 (3)
2022–9	40 (4)	0 (0)	0 (0)	0 (0)	40 (2)
2022–10	40 (4)	0 (0)	0 (0)	0 (0)	40 (2)
2022–11	66 (6)	5 (2)	12 (5)	28 (5)	111 (5)
2022–12	49 (4)	4 (1)	0 (0)	0 (0)	53 (2)
Age at enrollment, y					
0–4	131 (12)	15 (6)	6 (3)	67 (11)	219 (10)
5–9	65 (6)	4 (1)	1 (0)	79 (13)	149 (7)
10–14	59 (5)	8 (3)	4 (2)	69 (12)	140 (6)
15–19	128 (11)	30 (11)	17 (7)	69 (12)	244 (11)
20–24	114 (10)	19 (7)	25 (11)	52 (9)	210 (9)
25–54	493 (44)	138 (51)	132 (57)	195 (33)	958 (43)
≥55	139 (12)	57 (21)	47 (20)	65 (11)	308 (14)
Sex^a^					
Male	509 (45)	122 (46)	92 (41)	270 (47)	993 (45)
Female	620 (55)	146 (54)	135 (59)	307 (53)	1208 (55)
Presence of symptoms in preceding 14 d					
Fever	818 (72)	159 (59)	184 (79)	27 (5)	1188 (53)
Cough	906 (80)	248 (92)	212 (91)	79 (13)	1445 (65)
Fatigue	975 (86)	248 (92)	216 (93)	50 (8)	1489 (67)
Headache	922 (82)	240 (89)	201 (87)	55 (9)	1418 (64)
Muscle pain	784 (69)	224 (83)	199 (86)	37 (6)	1244 (56)
Sore throat	309 (27)	77 (28)	74 (32)	12 (2)	472 (21)
Difficulty breathing	251 (22)	112 (41)	43 (19)	10 (2)	416 (19)
Loss of taste	157 (14)	104 (38)	78 (34)	14 (2)	353 (16)
Loss of smell	121 (11)	85 (31)	43 (19)	4 (1)	253 (11)
Nausea	213 (19)	59 (22)	37 (16)	3 (1)	312 (14)
Vomiting	162 (14)	26 (10)	25 (11)	2 (0)	215 (10)
Diarrhea	115 (10)	22 (8)	26 (11)	5 (1)	168 (8)

Prospective Surveillance and Household Contacts of SARS-CoV-2–Positive Index Cases From Burkina Faso, Frequency (%) by Characteristic					
Characteristic	Prospective Surveillance by SARS-CoV-2 RT-PCR and/or Rapid Antigen Test			Household Contacts	All Prospectively Enrolled Participants
	Negative	Positive			
		Not Enrolled in Household Study	Enrolled in Household Study		
N	1572	208	15	50	1845
Enrollment Year–Month					
2021–6	3 (0)	1 (0)	0 (0)	0 (0)	4 (0)
2021–7	8 (1)	0 (0)	0 (0)	0 (0)	8 (0)
2021–8	25 (2)	1 (0)	0 (0)	0 (0)	26 (1)
2021–9	18 (1)	2 (1)	0 (0)	0 (0)	20 (1)
2021–10	30 (2)	1 (0)	3 (20)	19 (38)	53 (3)
2021–11	29 (2)	4 (2)	2 (13)	5 (10)	40 (2)
2021–12	478 (30)	147 (71)	5 (33)	11 (22)	641 (35)
2022–1	200 (13)	23 (11)	1 (7)	7 (14)	231 (13)
2022–2	50 (3)	1 (0)	0 (0)	0 (0)	51 (3)
2022–3	72 (5)	0 (0)	0 (0)	0 (0)	72 (4)
2022–4	45 (3)	0 (0)	0 (0)	0 (0)	45 (2)
2022–5	66 (4)	5 (2)	1 (7)	1 (2)	73 (4)
2022–6	128 (8)	2 (1)	0 (0)	0 (0)	130 (7)
2022–7	132 (8)	1 (0)	0 (0)	0 (0)	133 (7)
2022–8	151 (10)	7 (3)	0 (0)	0 (0)	158 (9)
2022–9	137 (9)	13 (6)	3 (20)	7 (14)	160 (9)
Age at enrollment, y^b^					
0–4	436 (28)	10 (5)	2 (13)	14 (28)	462 (25)
5–9	48 (3)	3 (1)	0 (0)	8 (16)	59 (3)
10–14	44 (3)	4 (2)	0 (0)	4 (8)	52 (3)
15–19	79 (5)	4 (2)	2 (13)	9 (18)	94 (5)
20–24	165 (10)	24 (12)	4 (27)	5 (10)	198 (11)
25–54	691 (44)	143 (69)	7 (47)	8 (16)	849 (46)
≥55	107 (7)	20 (10)	0 (0)	2 (4)	129 (7)
Sex					
Male	814 (52)	103 (50)	6 (40)	23 (53)	946 (52)
Female	756 (48)	105 (50)	9 (60)	20 (47)	890 (48)
Presence of symptoms in preceding 14 d	677 (43)	81 (39)	11 (73)	4 (8)	773 (42)
Fever	974 (62)	149 (72)	14 (93)	4 (8)	1141 (62)
Cough	477 (30)	116 (56)	7 (47)	4 (8)	604 (33)
Fatigue	586 (37)	131 (63)	10 (67)	2 (4)	729 (40)
Headache	326 (21)	95 (46)	4 (27)	3 (6)	428 (23)
Muscle pain	185 (12)	64 (31)	3 (20)	2 (4)	254 (14)
Sore throat	68 (4)	13 (6)	0 (0)	0 (0)	81 (4)
Difficulty breathing	52 (3)	30 (14)	1 (7)	0 (0)	83 (4)
Loss of taste	45 (3)	25 (12)	1 (7)	0 (0)	71 (4)
Loss of smell	107 (7)	23 (11)	1 (7)	1 (2)	132 (7)
Nausea	123 (8)	9 (4)	0 (0)	3 (6)	135 (7)
Vomiting	149 (9)	5 (2)	1 (7)	2 (4)	157 (9)
Diarrhea	677 (43)	81 (39)	11 (73)	4 (8)	773 (42)
Self-reported SARS-CoV-2 vaccination status at enrollment					
Unvaccinated	1463 (93)	183 (88)	15 (100)	48 (96)	1709 (93)
Vaccinated	109 (7)	25 (12)	0 (0)	2 (4)	136 (7)

Abbreviations: Ig, immunoglobulin; RT-PCR, reverse-transcription polymerase chain reaction; SARS-CoV-2, severe acute respiratory syndrome coronavirus 2.

^a^The numbers for sex do not add up due to missing information on this variable.

^b^The numbers in the age groups do not add up because we were unable to categorize the age of 2 study participants due to insufficient information.

### Prospective Surveillance Participants in Madagascar

Between May 2021 and December 2022, the prospective surveillance system in Madagascar captured 1632 febrile illness cases, 31% (503/1632) of which were SARS-CoV-2–positive by rapid antigen or rRT-PCR testing at enrollment (Table 1*B*). Moreover, 55% (1208/2228) of surveillance cases were in women. More than 74% (374/503) of SARS-CoV-2–positive prospective surveillance cases were in people aged >24 years, whereas only 56% (632/1129) of SARS-CoV-2–negative cases were in this age range. Fever, cough, fatigue, headache, and muscle pain were the most frequently reported symptoms among all prospectively enrolled surveillance cases in Madagascar.

### Prospective Surveillance Participants in Burkina Faso

Between June 2021 and September 2022, the prospective surveillance system in Burkina Faso captured 1795 febrile illness cases, 12% (223/1795) of which were SARS-CoV-2–positive by rapid antigen or rRT-PCR testing at enrollment (Table 1*C*). Moreover, 48% (890/1845) of surveillance cases were in women, and 76% (170/223) of SARS-CoV-2–positive and 51% (798/1572) of SARS-CoV-2–negative prospective surveillance cases were aged >24 years. Fever, cough, fatigue, headache, and muscle pain were the most frequently reported symptoms among all surveillance cases in Burkina Faso.

### Household Transmission Studies

Among the 581 enrolled household contacts in Madagascar, 53% (287/581) were seropositive for anti–SARS-CoV-2 IgM and/or IgG, 47% (275/581) were seronegative, and 3% (19/581) were missing this information. In Burkina Faso, 46% (23/50) were seropositive, 50% (25/50) were seronegative, and 4% (2/50) were missing their enrollment serological status. Among household contacts seronegative at enrollment, 41% (112/275) in Madagascar and 40% (10/25) in Burkina Faso were rRT-PCR– or rapid antigen test–positive, and an additional 16% (43/275) and 24% (6/25) seroconverted by the end of study follow-up (Table 2). Rates of incident rRT-PCR– or rapid antigen test–positive SARS-CoV-2 among household contacts during follow-up in Madagascar were similar by age group and number of household contacts. Too few household contacts enrolled in Burkina Faso to enable statistically valid descriptions of the association between covariates and the risk of incident SARS-CoV-2 infection among household contacts.

**Table 2.** Laboratory Assay and Household Analysis Results Among Contacts in 230 Households in Madagascar and 15 Households in Burkina Faso

Laboratory analysis								
Country Characteristic	Number of Participants	Number Seropositive at Enrollment	Number With Missing Serostatus at Enrollment, by End of Follow-up Status	Number Seronegative at Enrollment	Number (%) of Participants Seronegative at Baseline, by their Severe Acute Respiratory Syndrome Coronavirus 2 Status at the End of Follow-up			
					RT-PCR– or Rapid Antigen Test–Positive	Sero-Incident Infection Only		Status Missing
		IgG-only : IgM and IgG	(RT-PCR– or Rapid Antigen Test–Positive : IgG-Positive Only : Missing: Seronegative)			IgG-Positive Only	IgM- and IgG-Positive	
Madagascar								
All contacts	581	269 : 18	(2 : 1 : 13 : 3)	275	112 (41)	41 (15)	2 (1)	26 (9)
Age group, y								
0–4	61	19 : 4	(1 : 0 : 2 : 0)	35	16 (46)	2 (6)	0 (0)	3 (9)
5–9	75	30 : 2	(1 : 1 : 1 : 0)	40	15 (38)	9 (22)	0 (0)	1 (2)
10–14	72	27 : 2	(0 : 0 : 3 : 1)	39	17 (44)	6 (15)	0 (0)	5 (13)
15–19	71	28 : 1	(0 : 0 : 3 : 0)	39	18 (46)	6 (15)	0 (0)	5 (13)
20–24	50	32 : 0	(0 : 0 : 0 : 0)	18	9 (50)	3 (17)	1 (6)	1 (6)
25–54	189	94 : 7	(0 : 0 : 4 : 1)	83	29 (35)	12 (14)	1 (1)	10 (12)
≥55	63	39 : 2	(0 : 0 : 0 : 1)	21	8 (38)	3 (14)	0 (0)	1 (5)
Contacts per household								
1	76	36 : 2	(1 : 0 : 1 : 1)	35	9 (26)	9 (26)	0 (0)	3 (9)
2	100	63 : 2	(0 : 0 : 0 : 0)	35	15 (43)	4 (11)	0 (0)	4 (11)
3	180	69 : 5	(1 : 0 : 12 : 1)	92	39 (42)	13 (14)	1 (1)	8 (9)
4	92	50 : 3	(0 : 1 : 0 : 1)	37	15 (41)	4 (11)	0 (0)	3 (8)
5	55	15 : 6	(0 : 0 : 0 : 0)	34	12 (35)	6 (18)	0 (0)	5 (15)
6	30	15 : 0	(0 : 0 : 0 : 0)	15	8 (53)	0 (0)	0 (0)	1 (7)
7	7	5 : 0	(0 : 0 : 0 : 0)	2	1 (50)	0 (0)	0 (0)	0 (0)
8	8	2 : 0	(0 : 0 : 0 : 0)	6	3 (50)	2 (33)	0 (0)	1 (17)
9 or more	33	14 : 0	(0 : 0 : 0 : 0)	19	10 (53)	3 (16)	1 (5)	1 (5)
Burkina Faso								
All contacts	50	23 : 0	(1 : 1)	25	10 (40)	6 (24)	0 (0)	2 (8)
Age group, y								
0–4	13	2 : 0	(0 : 1)	10	5 (50)	2 (20)	0 (0)	0 (0)
5–9	8	4 : 0	(0 : 0)	4	1 (25)	2 (50)	0 (0)	1 (25)
10–14	4	2 : 0	(0 : 0)	2	1 (50)	0 (0)	0 (0)	0 (0)
15–19	9	6 : 0	(0 : 0)	3	0 (0)	1 (33)	0 (0)	1 (33)
20–24	5	3 : 0	(1 : 0)	1	1 (100)	0 (0)	0 (0)	0 (0)
25–54	9	6 : 0	(0 : 0)	3	2 (67)	0 (0)	0 (0)	0 (0)
≥55	2	0 : 0	(0 : 0)	2	0 (0)	1 (50)	0 (0)	0 (0)
Contacts per household								
1	5	1 : 0	(0 : 0)	4	3 (75)	0 (0)	0 (0)	0 (0)
2	4	2 : 0	(0 : 0)	2	1 (50)	1 (50)	0 (0)	0 (0)
3	12	2 : 0	(1 : 1)	8	3 (38)	3 (38)	0 (0)	0 (0)
4	8	5 : 0	(0 : 0)	3	3 (100)	0 (0)	0 (0)	0 (0)
5	5	4 : 0	(0 : 0)	1	0 (0)	1 (100)	0 (0)	0 (0)
9 or more	16	9 : 0	(0 : 0)	7	0 (0)	1 (14)	0 (0)	2 (29)

Household analysis								
Country	Proportion Immune^a^ at Enrollment (%, 95% CI)	Probability of Becoming Infected From Outside of the Household^b^ (%, 95% CI)	Household Secondary Attack Rate (%, 95% CI)					
			P1^c^	P2	P3	P4	P5	P1 to P5
Madagascar	48 (44–52)	7 (5–10)	17 (7–37)	33 (25–41)	16 (1–79)	15 (3–53)	11 (1–71)	28 (22–35)
Burkina Faso	42 (29–57)	16 (8–31)	31 (9–68)					31 (9–68)

Abbreviations: CI, confidence interval; Ig, immunoglobulin; RT-PCR, reverse-transcription polymerase chain reaction.

^a^Those positive for IgG and/or IgM by rapid antibody test were considered immune to severe acute respiratory syndrome coronavirus 2 infection for the duration of the relatively short period that each household was followed. As the model integrates over missing rapid antibody test results for enrollment measurements, the estimated proportion is not expected to be exactly equal to the observed proportion of household contacts who were rapid antibody test–positive at enrollment.

^b^The estimated probability of becoming infected through contact with someone other than the members of one's household over 10.4 days, the mean length of the assumed infectious period distribution.

^c^For Madagascar, P1, 06/25/2021 to 12/09/2021; P2, 12/10/2021 to 03/03/2022; P3, 03/04/2022 to 05/26/2022; P4, 05/27/2022 to 08/18/2022; and P5, 08/19/2022 to 12/12/2022. For Burkina Faso, P1–P5, 10/12/2021 to 11/11/2022.

An estimated 48% (95% confidence interval [CI]: 44%–52%) of all participating household contacts in Madagascar and 42% (95% CI: 29%–57%) in Burkina Faso were not susceptible to SARS-CoV-2 infection at enrollment (Table 3). The estimated infection probability from exposures outside the household during a 10.4-day period (mean length of the infectious period) was 7% (95% CI: 5%–10%) in Madagascar and 16% (95% CI: 8%–31%) in Burkina Faso. The estimated household secondary attack rate for Burkina Faso was 31% (95% CI: 9%–68%) throughout follow-up. In Madagascar, the estimated household secondary attack rate ranged from 11% (95% CI: 1%–71%) between 19 August 2022 and 12 December 2022 to 33% (95% CI: 25%–41%) between 10 December 2021 and 3 March 2022; the average household secondary attack rate was 28% (95% CI: 22%–35%) over the entire household study period in Madagascar.

**Table 3.** Input and Calibrated Parameter Values and Descriptive Statistics From 100 Simulated Epidemics Using the Calibrated Susceptible, Infectious, and Recovered Epidemic Models for Burkina Faso and Madagascar

Parameter	Description	Estimate (95% Confidence Interval^a^)	
		Burkina Faso	Madagascar
Calibrated			
δ	Proportional reduction in susceptibility after recovery from a primary SARS-CoV-2 infection	0.473 (.471–.480)	0.217 (.000–.366)
$\rho_{1}$	Proportion of symptomatic SARS-CoV-2 infections detected during prospective surveillance	0.101 (.098–.104)	0.123 (.086–.198)
Assumed or estimated from external data			
*γ*	7/mean duration of the infectious period	7/10.4 d	7/10.4 d
Time 0	Start date	03/08/2021	03/03/2020
$p_{r}$	Estimated probability of transmission per close contact: periods 1 to 5^b^	0.036 (.010–.119)	0.031 (.024–.041)
*b*	Estimated daily probability of infection from exposures to infection occurring outside of the household	0.017 (.008–.036)	0.007 (.005–.010)
*q*	Estimated proportion (%) of household contacts who are not susceptible at enrollment into the household transmission study	42 (29–57)	48 (44–52)
propI	Proportion of population infectious at time 0	1/80 000	1/220 000
w_v_	Wavelength of the cosine component of the temporal forcing function	37 wk	23 wk
delay	Temporal shift in the cosine component of the temporal forcing function	12 wk	0 wk
Simulation output and observed case counts			
Observed number of SARS-CoV-2–positive cases with symptomatic onset during prospective follow-up^c^		223	503
Model estimated (1000 synthetic epidemics): mean (standard error)			
Number of SARS-CoV-2–positive surveillance cases that would have been detected during prospective follow-up^c^		683 (2010)	573 (2301)
Number of SARS-CoV-2 infections during prospective follow-up		6572 (19 400)	4641 (18 652)
Seroprevalence at the start of prospective follow-up		0.35	0.105
Seroprevalence at the end of prospective follow-up		0.51	0.112

Abbreviation: SARS-CoV-2, severe acute respiratory syndrome coronavirus 2.

^a^Estimated by profile likelihood using a modified iterated filtering algorithm implemented by the *pomp* library in R (R Statistical Computing Foundation, v 4.2.3).

^b^For Madagascar, P1, 06/25/2021 to 12/09/2021; P2, 12/10/2021 to 03/03/2022; P3, 03/04/2022 to 05/26/2022; P4, 05/27/2022 to 08/18/2022; and P5, 08/19/2022 to 12/12/2022. For Burkina Faso, P1–P5, 10/12/2021 to 11/11/2022.

^c^The observed number of laboratory-confirmed, symptomatic SARS-CoV-2 cases included here differs from the numbers listed in Tables 1*B* and 1*C*. At the Madagascar site, 7 prospective surveillance cases were excluded because they experienced symptom onset before the earliest prospective surveillance visit. In additional, household contacts with laboratory-confirmed symptomatic infection at enrollment into the household study were included in the model along with the prospective surveillance cases (Burkina Faso, 2; Madagascar, 8).

### Epidemic Modeling of Surveillance Data

**Figure 1.**
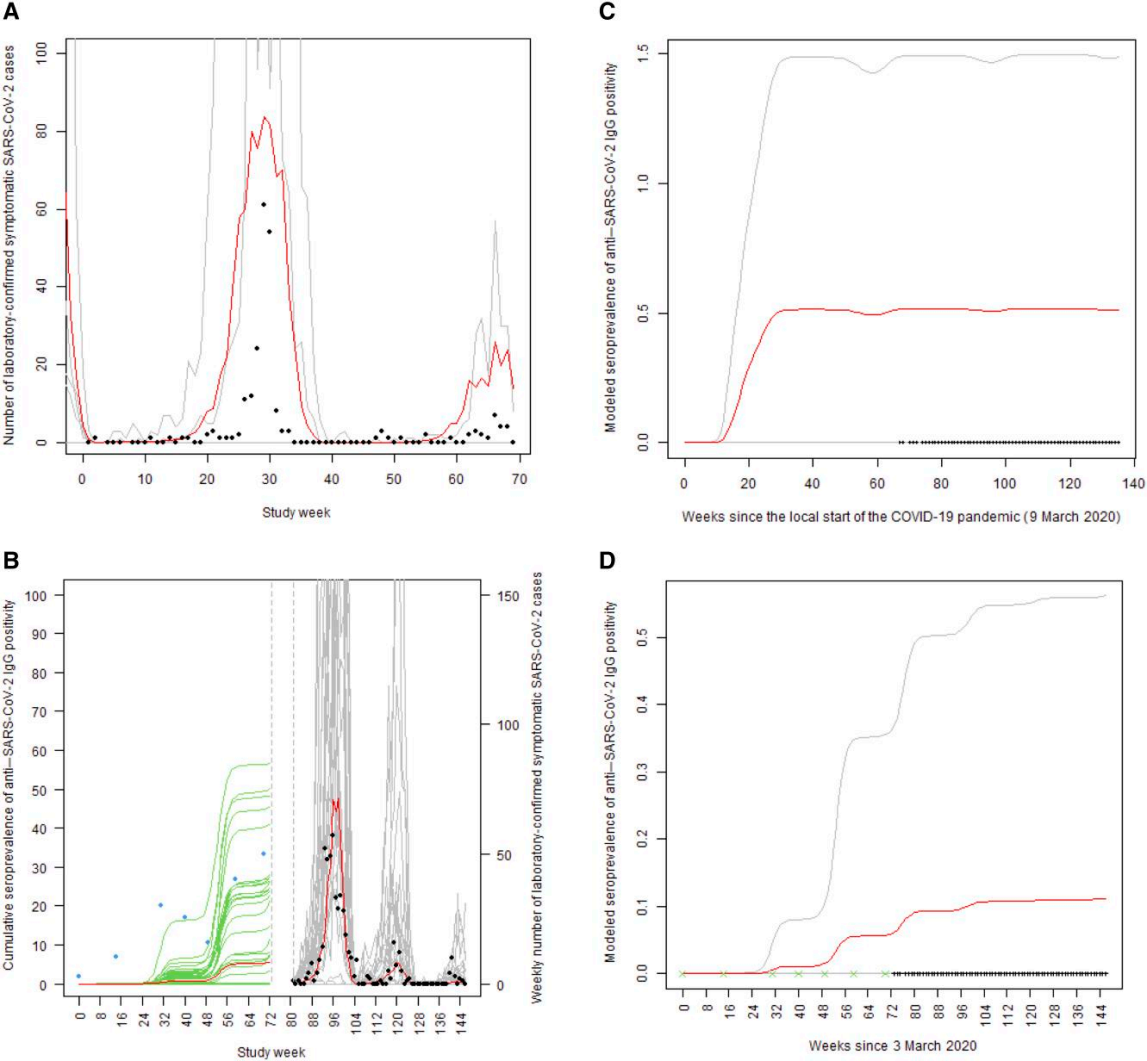
Observed and susceptible, infectious, and recovered (SIR)–modeled weekly frequency of reverse-transcription polymerase chain reaction–or rapid antigen test–positive symptomatic SARS-CoV-2 cases for the period of prospective surveillance in Burkina Faso (*A*) and Madagascar (*B*). Observed case counts are shown as solid black points. The red line plots the weekly average for 1000 synthetic epidemics, a randomly selected 10% of which are shown in gray. For Madagascar, the period seroprevalence of anti–SARS-CoV-2 IgG positivity among retrospectively analyzed serologic specimens collected from febrile illness cases (blue solid points) is compared to the model predicted proportion of individuals with a history of at least 1 SARS-CoV-2 infection (mean of 1000 epidemics, red; 100 sample epidemics, green). Dashed vertical gray lines denote the 3-week period during which prospective surveillance in Madagascar was being initiated. SIR model–derived seroprevalence curve for SARS-CoV-2 IgG seropositivity (mean, red; 95% confidence bands, gray) in Burkina Faso (*C*) and Madagascar (*D*) starting in early March 2020. The data collection times are indicated as ticks on the *x*-axis, with green indicating the start of each collection period for retrospectively analyzed archived serum specimens. Abbreviations: COVID-19, coronavirus disease 2019; Ig, immunoglobulin; SARS-CoV-2, severe acute respiratory syndrome coronavirus 2.

**Figure 2.**
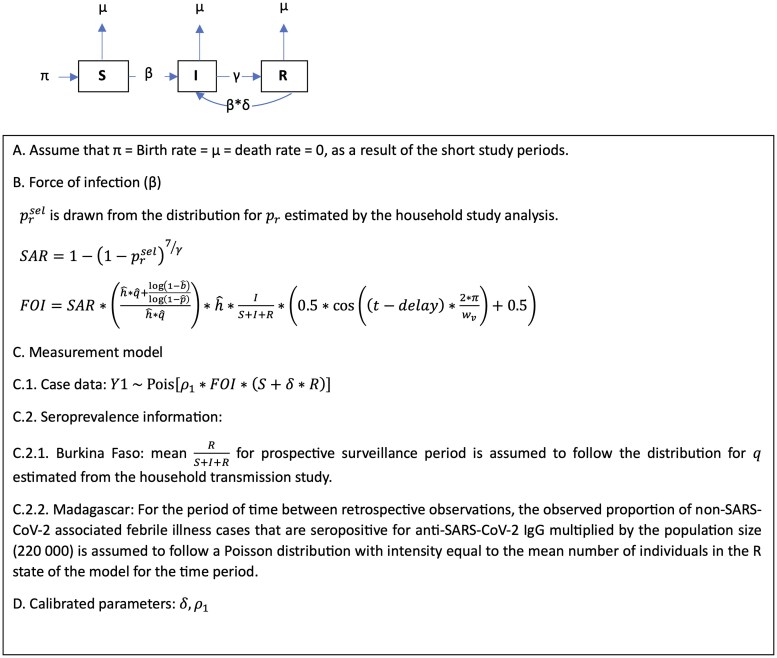
Diagram of dynamic epidemic model describing the transmission of SARS-CoV-2 at surveillance SETA/COVIA surveillance sites in Madagascar and Burkina Faso. Abbreviations: FOI, force of infection; Ig, immunoglobulin; SAR, secondary attack rate; SARS-CoV-2, severe acute respiratory syndrome coronavirus 2.

In both countries, the number of SARS-CoV-2–positive cases captured by prospective surveillance showed an initial peak followed by smaller peaks (Figure 1), a behavior replicated by the calibrated SIR model (model parameters in Table 3). One hundred randomly selected simulated epidemic curves are shown per country in Figure 1. The model-predicted seroprevalence of anti–SARS-CoV-2 IgG positivity increased during the prospective follow-up in each country (Figure 2, Table 3). The estimated number of SARS-CoV-2 infections was 9 times greater in Madagascar at 4641, whereas the observed number of COVID-19 cases was 503 (Table 3). This ratio was even greater in Burkina Faso.

## DISCUSSION

Here, we provide estimates of the observed burden of SARS-CoV-2–associated outcomes according to prospective monitoring of two populations in Burkina Faso and Madagascar. SARS-CoV-2 infections in Burkina Faso and Madagascar were estimated to be at least nine times the observed number of healthcare-ascertained, laboratory-confirmed symptomatic cases.

Our work illustrates that the integration of multiple data types can provide a comprehensive picture of the epidemiology of an infectious disease in resource-limited settings. Considering each data type in this study alone, including the household transmission study, the surveillance data, and the archived specimens, would have provided limited understanding of SARS-CoV-2 local epidemiology in each African setting. Integrating these data within a relatively simple mathematical model of disease transmission provided additional insights, for example, the likely total infection burden in each population. This approach could be more widely applied to characterize the epidemiology of vaccine-preventable infections in low-resource settings. In addition, calibrated transmission models could be extended to in silico interrogation of the potential effects of different vaccine delivery strategies.

Although the integration of data from multiple sources is a strength of our study, it also represents an important limitation. Because data from multiple sources were collected for various purposes, integrating these data required several untestable assumptions. For example, our modified SIR model incorporated the common assumption that every member of the catchment population in each study area's surveillance system was equally likely to have potentially infectious contacts with every other member (homogeneous mixing), an assumption that is almost certainly not true. To address the potential for bias associated with the homogeneous mixing assumption, we estimated the average number of daily contacts from the household transmission study analysis results that, in turn, relied on the assumption that the household contacts enrolled in the transmission study were representative of the average member of the wider population who was susceptible during the prospective surveillance period. The relatively small sample size in the household transmission study in Burkina Faso is an additional study limitation, although we propagated the statistical uncertainty associated with that small sample size to the calibration of the country's SIR model.

Our findings emphasize the critical role of integrated disease surveillance during both epidemic and interepidemic periods. High-resolution data on disease burden and epidemiological patterns are essential to inform public health strategies for controlling outbreaks that could escalate into pandemics. The results also underscore the need for investment in diagnostic infrastructure, such as point-of-care rapid diagnostic tests, in these settings. For instance, antibody positivity detected in archived samples collected before the pandemic in this study suggests a lack of specificity, potentially due to cross-reactivity with other viruses, including coronaviruses. Additionally, in the absence of high-resolution epidemiological data from systematic perspective surveillance, integrative disease modeling approaches provide valuable insights into the true burden of disease.

Furthermore, the household secondary attack rates observed in our study were comparable to other rates reported from settings without vaccination programs, often during the early phases of the pandemic [24]. However, the observed rate was higher than what we documented in a household transmission study in the Philippines [12]. The variation in household attack rates across different settings could be explained by various factors, including household size, vaccine coverage, which was very low in Madagascar and Burkina Faso, and whether the settings are rural or urban.

In conclusion, because SARS-CoV-2 vaccines are effective against severe outcomes, healthcare efforts have shifted to living with the virus. While we did not know about circulating variants at the time of study, the clinically milder nature of post-Delta variants put socioeconomic restoration back as a priority in resource-limited settings. Local capacity enhancement for surveillance, including genomic sequencing and vaccine manufacturing, together with investment in clinical and epidemiological research, is imperative. Our integrated approach using available data from multiple sources provides promise for strengthening epidemiologic research capacity in these settings.

## Supplementary Material

ciaf041_Supplementary_Data

## Supplementary Data

Supplementary materials are available at *Clinical Infectious Diseases* online. Consisting of data provided by the authors to benefit the reader, the posted materials are not copyedited and are the sole responsibility of the authors, so questions or comments should be addressed to the corresponding author.

## Notes

***Author Contributions.*** All authors interpreted the data, reviewed the manuscript critically for important intellectual content, and approved the final draft of the manuscript.

***Acknowledgments.*** The authors express their deep gratitude to the participants in this study, the community health workers and field staff for their hard work, and the administration and health officials who generously provided facilities and support for the conduct of the study.

***Financial support***. The Swedish International Development Agency provided financial support to the International Vaccine Institute for this research.

***Supplement sponsorship.*** This article appears as part of the supplement “COVID-19 Vaccine Research in Africa,” sponsored by the International Vaccine Institute and funded by the Coalition for Epidemic Preparedness Innovations (CEPI).
