# Supplementary material for: COVID-19 Surveillance in Madagascar and Urban Burkina Faso: Addressing Underreporting of Disease Burden Through Integrative Analysis of Diverse Data Streams
Source: Clin Infect Dis. 2025 Jul 22;80(Suppl 1):S16–28. doi: 10.1093/cid/ciaf041 (PMC12282513; doi:10.1093/cid/ciaf041)
Supplement: ciaf041_Supplementary_Data [file ciaf041_supplementary_data.docx]

**Coronavirus Disease 2019 Surveillance in Madagascar and Urban Burkina Faso: Addressing Underreporting of Disease Burden Through Integrative Analysis of Diverse Data Streams**

**Supplementary Materials**

The following are supplementary methods and results to support the manuscript entitled “Coronavirus Disease 2019 Surveillance in Madagascar and Urban Burkina Faso: Addressing Underreporting of Disease Burden Through Integrative Analysis of Diverse Data Streams”.

The document is organized into the following sections:

Section 1. Supplementary methods……………………………………….…………………………………….Page 2

Section 2. Input files for the analyses of the household transmission studies in Burkina Faso and Madagascar………………………………………………………………………….……………………………….Page 5

Section 3. Output files from the analyses of the household transmission studies in Burkina Faso and Madagascar………………………………………………………………..……………………………….Page 64

Section 4. Input files for the calibration of each country’s Susceptible, Infectious, and Recovered (SIR) mathematical model ……………………………………………..………………………….Page 67

Section 5. R code and data for implementing the exploratory test-negative case control study for vaccination in Burkina Faso………………………………………………………….……………………….Page 88

**Section 1. Supplementary methods**

*Statistical analyses*

*Data elements.* Data collection for the retrospective Madagascar SETA specimens is described elsewhere [1]. For this analysis, only the date range of enrollment and the rapid antibody test results were available for each archived SETA specimen. The prospective surveillance for febrile illness captured more detailed information, including the date of enrollment, age, sex, 14-day history of COVID-19 associated symptoms, earliest onset date for symptoms reported, treatment received for the current illness episode, history of comorbid risk factors, other risk factors for exposure to SARS-CoV-2, rapid antigen test results (positive, negative, and indeterminate/inconclusive/not-conducted), and rRT-PCR test results (positive, negative, and invalid/not-conducted). In addition to the baseline data elements captured by the prospective surveillance system, rapid antibody test (IgM, IgG, IgM/IgG, negative, or inconclusive/not-conducted), rRT-PCR test, and rapid antigen test results were collected at baseline and follow-up visits with the household contacts of SARS-CoV-2 positive cases detected through prospective surveillance. Though self-reported receipt of a SARS-CoV-2 vaccine was collected at the Burkina Faso site, the reported vaccination coverage in the Burkina Faso households was too sparse to provide any meaningful information, leading to this variable’s exclusion from further analysis. Note that SARS-CoV-2 positive cases detected by prospective surveillance could also, by design, participate in the household study as index cases.

Analytic methods

Data summaries were generated to provide a basic description of the epidemiology of SARS-CoV-2 and COVID-19 in the two sub-Saharan study populations. A two-stage approach was employed to conduct an integrated analysis of these data. First, data collected from the household transmission study were used to estimate the country-specific transmission potential of SARS-CoV-2, and then the results of this analysis informed the fitting of an epidemiologic mass-action model to the weekly retrospective and/or prospective surveillance data.

Household study analysis

This stage of the analysis estimated the household secondary attack rate (SAR), the probability that an infective individual will transmit SARS-CoV-2 to a household contact during his/her infectious period, and the daily probability of being infected through exposures occurring outside of the household ($b$). For the purposes of the household analysis, SARS-CoV-2 infection was defined as either 1) a positive rapid antigen or rRT-PCR test during study follow-up of a household and/or 2) a negative baseline rapid antibody test paired with an IgG and/or IgM positive rapid antibody test at the final follow-up visit. Symptomatic SARS-CoV-2 infection was defined as infection detected by rRT-PCR or rapid antigen test with concurrent self-report of a COVID-19 associated symptom. Symptom onset was defined as the date of earliest symptom occurrence. Where missing, the onset date for symptomatic infections was assumed to fall between the date of the study visit during which symptoms were first reported and the latest of either 21-days prior or, where available, the date of the preceding study visit. All SARS-CoV-2 infections were assumed potentially infectious, with onset of infectiousness occurring at symptom onset for those reporting symptoms. For asymptomatic SARS-CoV-2 infections detected by rapid antigen or rRT-PCR, onset of infectiousness is assumed to occur between the dates of the first positive test during study follow-up and the preceding study visit. Onset of infectiousness among asymptomatic infections detected by paired rapid antibody tests is assumed to occur between the dates of the two rapid antibody tests involved. A positive rapid antibody/antigen or rRT-PCR test at enrollment is assumed to be a strong indicator of non-susceptibility to SARS-CoV-2 infection during the relatively-short duration of the household study follow-up period. Household members with a positive rapid antigen or rRT-PCR test at enrollment are considered potentially infectious.

A chain-binomial model [2] was fit to the household data for each country. Corrections were applied to account for the index-cluster nature of this study design [2]. A hybrid expectation maximization (EM) and Monte Carlo EM algorithm [3] was employed to efficiently integrate over the missing information regarding immunity status at enrollment and/or the date of onset of infectiousness for asymptomatic SARS-CoV-2 infections among household contacts. The model estimated the probability *q* that a household contact was already immune at enrollment, as well as the daily probability *b* of infection due to exposures to SARS-CoV-2 occurring outside of the household. Where sufficient data were available, efforts were made to estimate time-period ($r$) specific probabilities of infection per daily person-to-person contact between members of the same household ($p_{r}$). There were only sufficient data in Burkina Faso to estimate a single $p_{r}$_;_ additional data resources in Madagascar permitted the estimation of a $p_{r}$ for each of five non-overlapping time periods. The distribution for the length of the infectious period of SARS-CoV-2 ($\Delta_{t}$) was assumed known with a mean duration of $\bar{\Delta}$ days (*t* in 1…16 days). The incubation and latent periods were both assumed to follow the same distribution $\nabla_{t}$ for (t in 1…14 days), with greater probability mass at the shorter end of this range. The SAR_r_ is estimated by $\left[ 1-\sum_{t=1}^{16} \left( 1-p_{r}*\Delta_{t} \right) \right]$. An equivalently-scaled measure of the risk of SARS-CoV-2 among household contacts associated with exposure to non-household sources is estimated by $CPI=1-\left( 1-b_{c} \right)^{\bar{\Delta}}$.

This chain-binomial model was fit using TranStat (version 3.0) [4]. The input files for each country level analysis are provided here in the Supplementary Materials. The estimated distributions for the logits of *b,* $p_{r}$ and $q$ contribute to the calibration of the epidemiologic compartmental model using the surveillance data.

Surveillance data analysis

*Model.* A modified Susceptible, Infectious, Recovered (SIR) model (Figure 1) was formulated in order to model the epidemiology of the SARS-CoV-2 at each study site. The parameter $propI$ represents the proportion of the population that was infectious at the start of each model’s run, time 0 (early March 2020 for both countries), which we assume (for both countries) to be equivalent to 1 infectious individual. Each model was run from Time 0 until the end of prospective data collection in the country. Prospective data collection started over a year (Burkina Faso, 66 weeks and Madagascar, 64 weeks) after time 0. In the case of Madagascar, archived surveillance specimens were available for the period between time 0 and the start of prospective surveillance. The SIR model tracks the history of exposure to primary infection by allowing individuals in the Recovered state to be re-infected with reduced susceptibility by a factor of $\delta$, and those in the Recovered state are assumed to remain anti-SARS-CoV-2 IgG positive for the duration of the period of time being modeled. Gamma (γ) denotes the rate of loss of infectiousness and is fixed at the inverse of the mean duration of the assumed infectious period distribution for the household analysis ($7/\bar{\Delta}$). The impacts of birth, migration, and death during this short study period are assumed negligible, so we parsimoniously assume the model population is closed.

Force of infection

It is assumed that SARS-CoV-2 transmits via close person-to-person contact, similar to those occurring within the household. Under this assumption, we can estimate the mean number of within-household-like, potentially-infectious contacts that each susceptible individual experienced daily outside of the household during the time period that the household transmission study was conducted as $\hat{n}=\frac{log\left( 1-\hat{b} \right)}{log\left( 1-\hat{p} \right)}$. Given the average number of individuals enrolled in the households in the transmission study ($\hat{h}$), the estimated proportion of household contacts who were not susceptible to infection as enrollment ($\hat{q}$), and the estimate for n, we can calculate the ratio of total potentially-infectious contacts to within-household contacts per day as $\frac{\hat{n}+\hat{q}*\hat{h}}{\hat{q}*\hat{h}}$. The estimate for R0 at time 0 is therefore $\hat{R0}=\frac{\hat{n}+\hat{q}*\hat{h}}{\hat{q}*\hat{h}}*\hat{SAR}$. The force of infection for a timepoint *t* is calculated as $R0*\gamma*\frac{I\left( t \right)}{\left( S\left( t \right)+I\left( t \right)+R\left( t \right) \right)}*\varphi$, where $\varphi$ represents the temporal forcing function $2*\left[ 0.5*\cos\left( \left( t-delay \right)*2*\frac{\pi}{w_{v}} \right)+0.5 \right]$. The wavelength ($w_{v}$) and the shift (*delay*) of the cosine function were determine through manual examination of the relative timing of peaks in the observed outcome data, with wavelengths of 23 and 37 weeks and delays of 0 and 12 weeks for Madagascar and Burkina Faso, respectively. The uncertainty in the estimates for $\hat{q}$, $\hat{p}$, and $\hat{b}$ were propagated to the SIR model by randomly drawing values at each time *t* from the normal distribution for each parameter’s logit distribution implied by their estimated mean and variance from the household analysis. Uncertainty in the estimate for $\hat{h}$ was assumed to be reflected in the size distribution for the households enrolled into each country’s transmission study.

*Measurement model.* The measurement model links the transmission model to the observed data. For both country sites, the weekly number of prospectively-ascertained, laboratory-confirmed symptomatic SARS-CoV-2 infections (Y1) was assumed to follow a Poisson distribution with mean $\rho_{1}*C$. To improve calibration for the Madagascar model, the number of individuals in the Recovered state is linked to the period seroprevalence of anti-SARS-CoV-2 IgG seropositivity among archived surveillance specimens through multiplication by the population size for the period extending from time 0 to the start of prospective surveillance.

*Model calibration.* Each country’s model was calibrated to the data using an iterated filtering algorithm (mif2), a simulation-based inference algorithm implemented in the *pomp* package of R (R Foundation for Statistical Computing, version 4.2.3). To improve model calibration, the following steps were repeated four times, with the results of each step informing the range of possible values for the subsequent steps. During each step, a profile likelihood approach was employed to calibrate values for $\delta$ and $\rho_{1}$ for each country’s model. For each parameter, the mif2 algorithm was run for ten filtering iterations with 50 particles at each of 25 values (10 replicates at each value) evenly spaced across the step’s value range for the parameter being calibrated. A LOESS smoothing function was used to estimated the likelihood profile for each parameter at the end of each step. Likelihood ratio based 95% confidence intervals were also estimated from the LOESS fit likelihood profile. For the first of the four steps, the full range of plausible values for each parameter were consider, but for each subsequent step the range of possible values was delineated by the minimum and maximum of the values for the parameter whose profile likelihood was in the top 50% of the LOESS fit.

Synthetic epidemics were simulated based upon the final calibrated model, incorporating the uncertainty associated with estimation of some model parameters from the household transmission study. The expected incidence curves, cumulative seroprevalence for IgG, and other summary features of the epidemiology of SARS-CoV-2 at these two study sites were summarized from 1000 synthetic epidemics.

Citations

1. Park, S.E., et al., *The Severe Typhoid Fever in Africa Program: Study Design and Methodology to Assess Disease Severity, Host Immunity, and Carriage Associated With Invasive Salmonellosis.* Clin Infect Dis, 2019. **69**(Suppl 6): p. S422-S434.
2. Yang, Y., I.M. Longini, Jr., and M.E. Halloran, *Design and Evaluation of Prophylactic Interventions Using Infectious Disease Incidence Data from Close Contact Groups.* J R Stat Soc Ser C Appl Stat, 2006. **55**(3): p. 317-330.
3. Yang, Y., et al., *A hybrid EM and Monte Carlo EM algorithm and its application to analysis of transmission of infectious diseases.* Biometrics, 2012. **68**(4): p. 1238-49.
4. Jing, Q.L., et al., *Household secondary attack rate of COVID-19 and associated determinants in Guangzhou, China: a retrospective cohort study.* Lancet Infect Dis, 2020. **20**(10): p. 1141-1150.

**Section 2. Input files for the analyses of the household transmission studies in Burkina Faso and Madagascar**

**1a. Burkina Faso**

c2p_contact.dat

0 168 223 1 0 0

0 224 279 2 0 0

1 280 335 3 0 0

1 336 391 4 0 0

2 392 447 4 0 0

2 448 503 4 0 0

2 504 521 4 0 0

3 56 111 0 0 0

3 112 167 0 0 0

3 168 223 1 0 0

4 112 167 0 0 0

4 168 223 1 0 0

7 112 167 0 0 0

7 168 223 1 0 0

8 112 167 0 0 0

8 168 223 1 0 0

9 168 223 1 0 0

9 224 279 2 0 0

10 168 223 1 0 0

10 224 279 2 0 0

11 168 223 1 0 0

11 224 279 2 0 0

12 168 223 1 0 0

12 224 279 2 0 0

13 392 447 4 0 0

13 448 503 4 0 0

13 504 521 4 0 0

14 392 447 4 0 0

14 448 503 4 0 0

14 504 521 4 0 0

community.dat

0 175 252

1 310 384

2 445 521

3 96 185

4 115 192

5 120 148

6 138 163

7 145 192

8 163 189

9 176 255

10 181 255

11 183 261

12 192 262

13 439 521

14 440 521

config.file

# input-path

*INSERT THE ADDRESS/PATH FOR YOUR WORKING DIRECTORY*

# output-path

*INSERT THE ADDRESS/PATH FOR YOUR WORKING DIRECTORY*

# min-max-days-and-probs-of-incubation-period

1

1 14

0.05772521 0.1092732 0.1434386 0.1557347 0.1477473 0.1254733 0.09659894 0.06791829 0.04381318 0.02601084 0.01424119 0.007201515 0.00336705 0.001456672

1 14

0.04267924 0.07942394 0.1066206 0.1221778 0.1259875 0.1196949 0.1060976 0.08840803 0.06959097 0.05192086 0.03680436 0.02483118 0.01596707 0.009795895

# primary-lower-upper-bounds-and-probs-of-infectious-days-relative-to-symptom-onset-day

1

1 16

1.00 1.00 1.00 1.00 1.00 1.00 0.80 0.80 0.60 0.60 0.40 0.40 0.30 0.30 0.10 0.10

# number-of-c2p-transmission-probabilities

1

# number-of-p2p-transmission-probabilities

1

# number-of-pathogenicity-groups

0

# number-of-preseason-immunity-groups

1

# number-of-time-independent-covariates

0

# number-of-time-dependent-covariates

0

# illness-period-as-a-time-dependent-covariate-for-infectivity

0:

# covariates-affecting-susceptibility-for-c2p-transmission

0:

# covariates-affecting-susceptibility-for-p2p-transmission

0:

# covariates-affecting-infectiousness-for-p2p-transmission

0:

# interactions-for-p2p-transmission

0:

# covariates-affecting-pathogenicity

0:

# covariates-affecting-preseason-immunity

0:

# equal-parameters

3:

1:1

1:2

1:3

# fixed-parameters

0:

# perform-simulation

0

# proportion-with-ambiguity-about-preimmunity-and-escape-status

0

# optimization-choice

1

# converge-criteria

1:

b 1.0e-3

p0 1.0e-3

u0 1.0e-3

# initial-estimates

1:3

b0 0.088

p0 0.027

u0 0.1

b0 0.084

p0 0.019

u0 0.5

b0 0.087

p0 0.016

u0 0.7

# search-bounds

1:

b0 1.0e-12 0.1

p0 1.0e-12 0.1

u0 1.0e-12 0.99

# perform-EM-algorithm

1

# min-number-of-possible-status-to-use-mcem

10000

# use-community-specific-weighting

0

# number-of-base-mcmc-samples

100

# number-of-burnin-mcmc-samples

100

# number-of-burnin-mcmc-iterations

10

# number-of-samplings-for-mc-error

0

# use-bootstrap-for-mc-error

0

# do-not-calculate-average-variance-for-mc-error

0

# check-missingness

0

# check-mixing

0

# check-runtime

0

# relative-infectivity-of-asymptomatic-case-for-simulation

0

# relative-infectivity-of-asymptomatic-case-for-estimation

1

# members-share-common-contact-history-within-communities

1

# automatically-generate-c2p-contact-file

1

# automatically-generate-p2p-contact-file

0

# use-c2p-offset

0

# use-p2p-offset

0

# adjust-for-selection-bias

1

# adjust-for-right-censoring

1

# prefix-index-cases

1

# epidemic-duration-for-calculating-CPI

10.4

# effective-lower-upper-bounds-of-infectious-days-relative-to-symptom-onset-day

0:

# covariates-for-calculating-SAR-provided

0:

# multiplier-for-calculating-R0

0:

# serial-division-of-epidemic-for-calculating-time-varying-R0

0:

# goodness-of-fit

1

# perform-statistical-test

0

# do-not-estimate-variance

0

# do-not-output-estimates

0

# simplify-output

0

# simplify-output-SAR

0

# simplify-output-R0

0

# run-transtat-silently

0

# write-error-log

0

impute.dat

14 0 0 0 -1 -1 1 127 185

27 1 0 0 -1 -1 1 115 192

28 0 0 0 -1 -1 1 142 192

29 0 0 0 -1 -1 1 142 192

36 1 1 0 -1 -1 1 145 192

46 0 0 0 -1 -1 1 226 255

50 0 0 0 -1 -1 1 229 261

56 0 0 0 -1 -1 1 232 262

p2p_contact.dat

0 175 252 0 0 0

1 310 384 0 0 0

2 445 521 0 0 0

3 96 185 0 0 0

4 115 192 0 0 0

5 120 148 0 0 0

6 138 163 0 0 0

7 145 192 0 0 0

8 163 189 0 0 0

9 176 255 0 0 0

10 181 255 0 0 0

11 183 261 0 0 0

12 192 262 0 0 0

13 439 521 0 0 0

14 440 521 0 0 0

pop.dat

0 0 0 1 1 195 0 -1 1 0 0 1 0

1 0 1 0 0 -1 1 252 0 0 0 1 0

2 0 0 1 1 197 0 -1 0 0 0 1 0

3 0 0 1 1 197 0 -1 0 0 0 1 0

4 0 0 1 1 197 0 -1 0 0 0 1 0

5 1 0 1 1 330 0 -1 1 0 0 1 0

6 1 0 1 1 339 0 -1 0 0 0 1 0

7 2 0 1 1 465 0 -1 1 0 0 1 0

8 2 0 1 1 483 0 -1 0 0 0 1 0

9 3 0 1 1 116 0 -1 1 0 0 1 0

10 3 1 0 0 -1 1 126 0 0 0 1 0

11 3 0 0 0 -1 1 127 0 0 0 1 0

12 3 0 0 0 -1 1 155 0 0 0 1 0

13 3 1 0 0 -1 1 127 0 0 0 1 0

14 3 0 1 0 -1 0 -1 0 0 0 1 0

15 3 1 0 0 -1 1 127 0 0 0 1 0

16 3 1 0 0 -1 1 185 0 0 0 1 0

17 3 1 0 0 -1 1 127 0 0 0 1 0

18 3 0 0 0 -1 1 127 0 0 0 1 0

19 3 1 0 0 -1 1 127 0 0 0 1 0

20 3 0 0 0 -1 1 155 0 0 0 1 0

21 3 0 0 0 -1 1 127 0 0 0 1 0

22 3 1 0 0 -1 1 127 0 0 0 1 0

23 3 1 0 0 -1 1 127 0 0 0 1 0

24 3 0 0 0 -1 1 127 0 0 0 1 0

25 3 1 0 0 -1 1 127 0 0 0 1 0

26 4 0 1 1 135 0 -1 1 0 0 1 0

27 4 0 0 0 -1 1 192 0 0 0 1 0

28 4 0 1 0 -1 0 -1 0 0 0 1 0

29 4 0 1 0 -1 0 -1 0 0 0 1 0

30 5 0 1 1 140 0 -1 1 0 0 1 0

31 5 0 0 0 -1 1 148 0 0 0 1 0

32 6 0 1 1 158 0 -1 1 0 0 1 0

33 6 1 0 0 -1 1 163 0 0 0 1 0

34 7 0 1 1 165 0 -1 1 0 0 1 0

35 7 0 1 1 178 0 -1 0 0 0 1 0

36 7 0 0 0 -1 1 192 0 0 0 1 0

37 7 0 0 0 -1 1 170 0 0 0 1 0

38 8 0 1 1 183 0 -1 1 0 0 1 0

39 8 0 1 1 189 0 -1 0 0 0 1 0

40 8 0 1 1 189 0 -1 0 0 0 1 0

41 8 0 0 0 -1 1 189 0 0 0 1 0

42 9 0 1 1 196 0 -1 1 0 0 1 0

43 9 0 1 1 204 0 -1 0 0 0 1 0

44 10 0 1 1 201 0 -1 1 0 0 1 0

45 10 1 0 0 -1 1 205 0 0 0 1 0

46 10 0 1 0 -1 0 -1 0 0 0 1 0

47 10 1 0 0 -1 1 205 0 0 0 1 0

48 11 0 1 1 203 0 -1 1 0 0 1 0

49 11 1 0 0 -1 1 261 0 0 0 1 0

50 11 0 1 0 -1 0 -1 0 0 0 1 0

51 12 0 0 0 -1 1 212 1 0 0 1 0

52 12 1 0 0 -1 1 262 0 0 0 1 0

53 12 1 0 0 -1 1 262 0 0 0 1 0

54 12 1 0 0 -1 1 262 0 0 0 1 0

55 12 1 0 0 -1 1 262 0 0 0 1 0

56 12 0 1 0 -1 0 -1 0 0 0 1 0

57 13 0 1 1 459 0 -1 1 0 0 1 0

58 13 1 0 0 -1 1 521 0 0 0 1 0

59 13 0 1 1 469 0 -1 0 0 0 1 0

60 13 1 0 0 -1 1 469 0 0 0 1 0

61 13 0 1 1 476 0 -1 0 0 0 1 0

62 14 0 1 1 460 0 -1 1 0 0 1 0

63 14 1 0 0 -1 1 521 0 0 0 1 0

64 14 0 1 1 483 0 -1 0 0 0 1 0

**1b. Madagascar**

c2p_contact.dat

0 224 279 0 0 0

0 280 335 0 0 0

1 224 279 0 0 0

1 280 335 0 0 0

2 280 335 0 0 0

2 336 391 1 0 0

3 280 335 0 0 0

3 336 391 1 0 0

4 280 335 0 0 0

4 336 391 1 0 0

5 280 335 0 0 0

5 336 391 1 0 0

6 280 335 0 0 0

6 336 391 1 0 0

7 280 335 0 0 0

7 336 391 1 0 0

8 336 391 1 0 0

9 280 335 0 0 0

9 336 391 1 0 0

10 280 335 0 0 0

10 336 391 1 0 0

11 280 335 0 0 0

11 336 391 1 0 0

13 336 391 1 0 0

13 392 447 2 0 0

14 336 391 1 0 0

14 392 447 2 0 0

15 336 391 1 0 0

15 392 447 2 0 0

16 336 391 1 0 0

16 392 447 2 0 0

19 336 391 1 0 0

19 392 447 2 0 0

21 336 391 1 0 0

21 392 447 2 0 0

23 336 391 1 0 0

23 392 447 2 0 0

24 336 391 1 0 0

24 392 447 2 0 0

26 336 391 1 0 0

26 392 447 2 0 0

28 336 391 1 0 0

28 392 447 2 0 0

29 336 391 1 0 0

29 392 447 2 0 0

30 336 391 1 0 0

30 392 447 2 0 0

31 336 391 1 0 0

31 392 447 2 0 0

32 336 391 1 0 0

32 392 447 2 0 0

33 336 391 1 0 0

33 392 447 2 0 0

34 336 391 1 0 0

34 392 447 2 0 0

35 336 391 1 0 0

35 392 447 2 0 0

36 336 391 1 0 0

36 392 447 2 0 0

36 448 503 3 0 0

37 336 391 1 0 0

37 392 447 2 0 0

38 336 391 1 0 0

38 392 447 2 0 0

39 336 391 1 0 0

39 392 447 2 0 0

39 448 503 3 0 0

40 336 391 1 0 0

40 392 447 2 0 0

40 448 503 3 0 0

41 336 391 1 0 0

41 392 447 2 0 0

41 448 503 3 0 0

43 392 447 2 0 0

43 448 503 3 0 0

44 392 447 2 0 0

44 448 503 3 0 0

45 336 391 1 0 0

45 392 447 2 0 0

46 392 447 2 0 0

46 448 503 3 0 0

47 392 447 2 0 0

47 448 503 3 0 0

48 392 447 2 0 0

48 448 503 3 0 0

49 448 503 3 0 0

49 504 559 4 0 0

50 448 503 3 0 0

50 504 559 4 0 0

50 560 615 4 0 0

51 448 503 3 0 0

51 504 559 4 0 0

51 560 615 4 0 0

52 448 503 3 0 0

52 504 559 4 0 0

52 560 615 4 0 0

53 448 503 3 0 0

53 504 559 4 0 0

53 560 615 4 0 0

54 448 503 3 0 0

54 504 559 4 0 0

54 560 615 4 0 0

55 448 503 3 0 0

55 504 559 4 0 0

55 560 615 4 0 0

57 504 559 4 0 0

57 560 615 4 0 0

58 504 559 4 0 0

58 560 615 4 0 0

60 504 559 4 0 0

60 560 615 4 0 0

61 504 559 4 0 0

61 560 615 4 0 0

62 504 559 4 0 0

62 560 615 4 0 0

63 504 559 4 0 0

63 560 615 4 0 0

64 504 559 4 0 0

64 560 615 4 0 0

65 616 671 4 0 0

65 672 709 4 0 0

66 616 671 4 0 0

66 672 709 4 0 0

67 616 671 4 0 0

67 672 709 4 0 0

68 616 671 4 0 0

68 672 709 4 0 0

69 616 671 4 0 0

69 672 709 4 0 0

70 616 671 4 0 0

70 672 709 4 0 0

71 616 671 4 0 0

71 672 709 4 0 0

72 224 279 0 0 0

72 280 335 0 0 0

72 336 391 1 0 0

73 224 279 0 0 0

73 280 335 0 0 0

73 336 391 1 0 0

74 224 279 0 0 0

74 280 335 0 0 0

74 336 391 1 0 0

75 280 335 0 0 0

75 336 391 1 0 0

76 280 335 0 0 0

76 336 391 1 0 0

77 280 335 0 0 0

77 336 391 1 0 0

78 280 335 0 0 0

78 336 391 1 0 0

79 280 335 0 0 0

79 336 391 1 0 0

80 280 335 0 0 0

80 336 391 1 0 0

80 392 447 2 0 0

81 280 335 0 0 0

81 336 391 1 0 0

82 280 335 0 0 0

82 336 391 1 0 0

83 280 335 0 0 0

83 336 391 1 0 0

83 392 447 2 0 0

84 280 335 0 0 0

84 336 391 1 0 0

85 280 335 0 0 0

85 336 391 1 0 0

86 280 335 0 0 0

86 336 391 1 0 0

87 280 335 0 0 0

87 336 391 1 0 0

88 280 335 0 0 0

88 336 391 1 0 0

89 280 335 0 0 0

89 336 391 1 0 0

89 392 447 2 0 0

90 280 335 0 0 0

90 336 391 1 0 0

91 280 335 0 0 0

91 336 391 1 0 0

92 280 335 0 0 0

92 336 391 1 0 0

93 280 335 0 0 0

93 336 391 1 0 0

94 280 335 0 0 0

94 336 391 1 0 0

94 392 447 2 0 0

95 280 335 0 0 0

95 336 391 1 0 0

96 280 335 0 0 0

96 336 391 1 0 0

96 392 447 2 0 0

97 280 335 0 0 0

97 336 391 1 0 0

98 280 335 0 0 0

98 336 391 1 0 0

98 392 447 2 0 0

99 280 335 0 0 0

99 336 391 1 0 0

100 280 335 0 0 0

100 336 391 1 0 0

100 392 447 2 0 0

101 280 335 0 0 0

101 336 391 1 0 0

101 392 447 2 0 0

102 280 335 0 0 0

102 336 391 1 0 0

103 280 335 0 0 0

103 336 391 1 0 0

104 280 335 0 0 0

104 336 391 1 0 0

105 280 335 0 0 0

105 336 391 1 0 0

106 280 335 0 0 0

106 336 391 1 0 0

107 280 335 0 0 0

107 336 391 1 0 0

107 392 447 2 0 0

108 280 335 0 0 0

108 336 391 1 0 0

108 392 447 2 0 0

109 280 335 0 0 0

109 336 391 1 0 0

109 392 447 2 0 0

110 280 335 0 0 0

110 336 391 1 0 0

111 280 335 0 0 0

111 336 391 1 0 0

112 280 335 0 0 0

112 336 391 1 0 0

113 280 335 0 0 0

113 336 391 1 0 0

113 392 447 2 0 0

114 280 335 0 0 0

114 336 391 1 0 0

115 280 335 0 0 0

115 336 391 1 0 0

115 392 447 2 0 0

116 280 335 0 0 0

116 336 391 1 0 0

116 392 447 2 0 0

117 280 335 0 0 0

117 336 391 1 0 0

117 392 447 2 0 0

118 280 335 0 0 0

118 336 391 1 0 0

119 280 335 0 0 0

119 336 391 1 0 0

120 336 391 1 0 0

121 336 391 1 0 0

121 392 447 2 0 0

122 280 335 0 0 0

122 336 391 1 0 0

122 392 447 2 0 0

123 280 335 0 0 0

123 336 391 1 0 0

123 392 447 2 0 0

124 280 335 0 0 0

124 336 391 1 0 0

124 392 447 2 0 0

126 336 391 1 0 0

126 392 447 2 0 0

127 336 391 1 0 0

127 392 447 2 0 0

128 280 335 0 0 0

128 336 391 1 0 0

128 392 447 2 0 0

129 280 335 0 0 0

129 336 391 1 0 0

129 392 447 2 0 0

130 336 391 1 0 0

130 392 447 2 0 0

131 336 391 1 0 0

131 392 447 2 0 0

132 280 335 0 0 0

132 336 391 1 0 0

133 336 391 1 0 0

133 392 447 2 0 0

134 336 391 1 0 0

134 392 447 2 0 0

135 336 391 1 0 0

135 392 447 2 0 0

136 336 391 1 0 0

136 392 447 2 0 0

137 336 391 1 0 0

137 392 447 2 0 0

138 336 391 1 0 0

138 392 447 2 0 0

139 336 391 1 0 0

139 392 447 2 0 0

140 336 391 1 0 0

140 392 447 2 0 0

141 336 391 1 0 0

141 392 447 2 0 0

142 336 391 1 0 0

142 392 447 2 0 0

143 336 391 1 0 0

143 392 447 2 0 0

144 336 391 1 0 0

144 392 447 2 0 0

145 336 391 1 0 0

145 392 447 2 0 0

146 336 391 1 0 0

146 392 447 2 0 0

147 336 391 1 0 0

147 392 447 2 0 0

148 336 391 1 0 0

148 392 447 2 0 0

149 336 391 1 0 0

149 392 447 2 0 0

150 336 391 1 0 0

150 392 447 2 0 0

151 336 391 1 0 0

151 392 447 2 0 0

152 336 391 1 0 0

152 392 447 2 0 0

153 336 391 1 0 0

153 392 447 2 0 0

154 336 391 1 0 0

154 392 447 2 0 0

155 336 391 1 0 0

155 392 447 2 0 0

156 336 391 1 0 0

156 392 447 2 0 0

157 336 391 1 0 0

157 392 447 2 0 0

158 336 391 1 0 0

158 392 447 2 0 0

159 336 391 1 0 0

159 392 447 2 0 0

160 336 391 1 0 0

160 392 447 2 0 0

161 336 391 1 0 0

161 392 447 2 0 0

162 336 391 1 0 0

162 392 447 2 0 0

163 336 391 1 0 0

163 392 447 2 0 0

164 336 391 1 0 0

164 392 447 2 0 0

165 336 391 1 0 0

165 392 447 2 0 0

166 336 391 1 0 0

166 392 447 2 0 0

167 336 391 1 0 0

167 392 447 2 0 0

168 336 391 1 0 0

168 392 447 2 0 0

169 336 391 1 0 0

169 392 447 2 0 0

170 336 391 1 0 0

170 392 447 2 0 0

171 336 391 1 0 0

171 392 447 2 0 0

172 336 391 1 0 0

172 392 447 2 0 0

173 336 391 1 0 0

173 392 447 2 0 0

174 336 391 1 0 0

174 392 447 2 0 0

175 336 391 1 0 0

175 392 447 2 0 0

176 336 391 1 0 0

176 392 447 2 0 0

177 336 391 1 0 0

177 392 447 2 0 0

178 336 391 1 0 0

178 392 447 2 0 0

179 336 391 1 0 0

179 392 447 2 0 0

180 336 391 1 0 0

180 392 447 2 0 0

181 336 391 1 0 0

181 392 447 2 0 0

182 336 391 1 0 0

182 392 447 2 0 0

183 336 391 1 0 0

183 392 447 2 0 0

184 336 391 1 0 0

184 392 447 2 0 0

185 336 391 1 0 0

185 392 447 2 0 0

186 336 391 1 0 0

186 392 447 2 0 0

187 336 391 1 0 0

187 392 447 2 0 0

188 336 391 1 0 0

188 392 447 2 0 0

189 336 391 1 0 0

189 392 447 2 0 0

190 336 391 1 0 0

190 392 447 2 0 0

191 336 391 1 0 0

191 392 447 2 0 0

192 336 391 1 0 0

192 392 447 2 0 0

193 336 391 1 0 0

193 392 447 2 0 0

194 336 391 1 0 0

194 392 447 2 0 0

195 336 391 1 0 0

195 392 447 2 0 0

196 336 391 1 0 0

196 392 447 2 0 0

197 336 391 1 0 0

197 392 447 2 0 0

198 336 391 1 0 0

198 392 447 2 0 0

199 336 391 1 0 0

199 392 447 2 0 0

200 336 391 1 0 0

200 392 447 2 0 0

201 336 391 1 0 0

201 392 447 2 0 0

202 336 391 1 0 0

202 392 447 2 0 0

203 392 447 2 0 0

203 448 503 3 0 0

204 392 447 2 0 0

204 448 503 3 0 0

205 448 503 3 0 0

205 504 559 4 0 0

206 448 503 3 0 0

206 504 559 4 0 0

207 448 503 3 0 0

207 504 559 4 0 0

208 448 503 3 0 0

208 504 559 4 0 0

209 448 503 3 0 0

209 504 559 4 0 0

209 560 615 4 0 0

210 448 503 3 0 0

210 504 559 4 0 0

210 560 615 4 0 0

211 448 503 3 0 0

211 504 559 4 0 0

212 448 503 3 0 0

212 504 559 4 0 0

213 448 503 3 0 0

213 504 559 4 0 0

213 560 615 4 0 0

214 448 503 3 0 0

214 504 559 4 0 0

214 560 615 4 0 0

215 504 559 4 0 0

215 560 615 4 0 0

217 504 559 4 0 0

217 560 615 4 0 0

218 504 559 4 0 0

218 560 615 4 0 0

219 504 559 4 0 0

219 560 615 4 0 0

220 392 447 2 0 0

220 448 503 3 0 0

220 504 559 4 0 0

220 560 615 4 0 0

221 504 559 4 0 0

221 560 615 4 0 0

222 504 559 4 0 0

222 560 615 4 0 0

223 616 671 4 0 0

223 672 709 4 0 0

224 616 671 4 0 0

224 672 709 4 0 0

225 616 671 4 0 0

225 672 709 4 0 0

226 616 671 4 0 0

226 672 709 4 0 0

227 616 671 4 0 0

227 672 709 4 0 0

community.dat

0 246 298

1 268 304

2 314 344

3 317 382

4 321 347

5 324 348

6 326 382

7 330 352

8 336 359

9 334 387

10 332 390

11 329 374

12 338 390

13 340 392

14 339 397

15 342 403

16 346 422

17 344 374

18 345 368

19 346 405

20 348 383

21 353 409

22 354 375

23 346 414

24 355 439

25 355 380

26 357 408

27 359 390

28 361 418

29 362 418

30 361 426

31 364 423

32 367 424

33 369 423

34 370 426

35 370 398

36 380 452

37 381 438

38 386 415

39 390 455

40 390 455

41 381 460

42 393 429

43 402 457

44 400 470

45 378 436

46 400 468

47 398 470

48 428 494

49 493 557

50 499 566

51 499 566

52 501 567

53 501 567

54 500 560

55 497 569

56 506 557

57 506 579

58 512 582

59 514 543

60 513 581

61 520 579

62 519 580

63 540 600

64 510 601

65 653 688

66 654 689

67 655 690

68 655 691

69 655 691

70 650 684

71 659 689

72 234 338

73 276 341

74 274 362

75 281 348

76 282 354

77 284 344

78 284 346

79 302 368

80 306 394

81 306 387

82 307 387

83 311 394

84 311 374

85 314 373

86 315 376

87 317 387

88 318 377

89 318 411

90 318 382

91 319 389

92 307 389

93 320 384

94 320 396

95 318 383

96 321 396

97 323 384

98 321 397

99 320 384

100 323 419

101 318 408

102 319 391

103 322 383

104 321 388

105 325 384

106 326 348

107 327 394

108 325 410

109 324 395

110 323 388

111 324 383

112 325 353

113 326 403

114 325 389

115 320 394

116 331 402

117 330 394

118 325 389

119 330 389

120 336 389

121 336 394

122 332 410

123 331 419

124 335 410

125 338 360

126 338 398

127 339 418

128 331 403

129 335 408

130 339 412

131 337 394

132 335 390

133 339 394

134 337 410

135 338 403

136 338 399

137 341 412

138 342 404

139 342 403

140 341 411

141 337 416

142 338 418

143 340 397

144 339 401

145 339 431

146 340 417

147 346 404

148 343 411

149 341 405

150 345 426

151 345 417

152 342 425

153 341 411

154 347 418

155 352 405

156 351 410

157 353 430

158 355 412

159 354 444

160 354 418

161 355 408

162 355 417

163 355 433

164 357 411

165 358 417

166 358 426

167 359 411

168 361 416

169 359 412

170 360 432

171 358 424

172 360 425

173 361 418

174 362 425

175 361 425

176 362 425

177 366 422

178 364 429

179 365 430

180 364 422

181 365 433

182 363 429

183 367 437

184 356 431

185 359 438

186 367 431

187 363 424

188 367 437

189 373 433

190 372 431

191 371 438

192 371 437

193 371 429

194 373 405

195 374 438

196 368 433

197 375 430

198 346 443

199 378 438

200 381 440

201 386 445

202 388 447

203 394 453

204 396 468

205 466 538

206 487 548

207 488 552

208 494 552

209 496 572

210 499 564

211 500 559

212 500 559

213 502 576

214 503 564

215 506 569

216 515 551

217 515 580

218 517 577

219 509 579

220 429 579

221 535 594

222 544 611

223 644 704

224 647 678

225 648 688

226 651 688

227 655 691

228 675 703

229 675 703

config.file, one single p_r_

# input-path

*INSERT THE ADDRESS/PATH FOR YOUR WORKING DIRECTORY*

# output-path

*INSERT THE ADDRESS/PATH FOR YOUR WORKING DIRECTORY*

# min-max-days-and-probs-of-incubation-period

1

1 14

0.05772521 0.1092732 0.1434386 0.1557347 0.1477473 0.1254733 0.09659894 0.06791829 0.04381318 0.02601084 0.01424119 0.007201515 0.00336705 0.001456672

1 14

0.04267924 0.07942394 0.1066206 0.1221778 0.1259875 0.1196949 0.1060976 0.08840803 0.06959097 0.05192086 0.03680436 0.02483118 0.01596707 0.009795895

# primary-lower-upper-bounds-and-probs-of-infectious-days-relative-to-symptom-onset-day

1

1 16

1.00 1.00 1.00 1.00 1.00 1.00 0.80 0.80 0.60 0.60 0.40 0.40 0.30 0.30 0.10 0.10

# number-of-c2p-transmission-probabilities

1

# number-of-p2p-transmission-probabilities

1

# number-of-pathogenicity-groups

0

# number-of-preseason-immunity-groups

1

# number-of-time-independent-covariates

0

# number-of-time-dependent-covariates

0

# illness-period-as-a-time-dependent-covariate-for-infectivity

0:

# covariates-affecting-susceptibility-for-c2p-transmission

0:

# covariates-affecting-susceptibility-for-p2p-transmission

0:

# covariates-affecting-infectiousness-for-p2p-transmission

0:

# interactions-for-p2p-transmission

0:

# covariates-affecting-pathogenicity

0:

# covariates-affecting-preseason-immunity

0:

# equal-parameters

3:

1:1

1:2

1:3

# fixed-parameters

0:

# perform-simulation

0

# proportion-with-ambiguity-about-preimmunity-and-escape-status

0

# optimization-choice

1

# converge-criteria

1:

b 1.0e-3

p0 1.0e-3

u0 1.0e-3

# initial-estimates

1:3

b0 0.088

p0 0.018

u0 0.1

b0 0.084

p0 0.019

u0 0.5

b0 0.087

p0 0.016

u0 0.7

# search-bounds

1:

b0 1.0e-12 0.1

p0 1.0e-12 0.1

u0 1.0e-12 0.99

# perform-EM-algorithm

1

# min-number-of-possible-status-to-use-mcem

10000

# use-community-specific-weighting

0

# number-of-base-mcmc-samples

100

# number-of-burnin-mcmc-samples

100

# number-of-burnin-mcmc-iterations

10

# number-of-samplings-for-mc-error

0

# use-bootstrap-for-mc-error

0

# do-not-calculate-average-variance-for-mc-error

0

# check-missingness

0

# check-mixing

0

# check-runtime

0

# relative-infectivity-of-asymptomatic-case-for-simulation

0

# relative-infectivity-of-asymptomatic-case-for-estimation

1

# members-share-common-contact-history-within-communities

1

# automatically-generate-c2p-contact-file

1

# automatically-generate-p2p-contact-file

0

# use-c2p-offset

0

# use-p2p-offset

0

# adjust-for-selection-bias

1

# adjust-for-right-censoring

1

# prefix-index-cases

1

# epidemic-duration-for-calculating-CPI

10.4

# effective-lower-upper-bounds-of-infectious-days-relative-to-symptom-onset-day

0:

# covariates-for-calculating-SAR-provided

0:

# multiplier-for-calculating-R0

5:

3.6 3.02

0 0

0 0

0 0

0 0

0 0

3.6 3.02

0 0

0 0

0 0

0 0

0 0

3.6 3.02

0 0

0 0

0 0

0 0

0 0

3.6 3.02

0 0

0 0

0 0

0 0

0 0

3.6 3.02

# serial-division-of-epidemic-for-calculating-time-varying-R0

0:

168 335 168

336 419 84

420 503 84

504 587 84

588 709 122

# goodness-of-fit

1

# perform-statistical-test

0

# do-not-estimate-variance

0

# do-not-output-estimates

0

# simplify-output

0

# simplify-output-SAR

0

# simplify-output-R0

0

# run-transtat-silently

0

# write-error-log

0

config.file, five p_r_’s

# input-path

*INSERT THE ADDRESS/PATH FOR YOUR WORKING DIRECTORY*

# output-path

*INSERT THE ADDRESS/PATH FOR YOUR WORKING DIRECTORY*

# min-max-days-and-probs-of-incubation-period

1

1 14

0.05772521 0.1092732 0.1434386 0.1557347 0.1477473 0.1254733 0.09659894 0.06791829 0.04381318 0.02601084 0.01424119 0.007201515 0.00336705 0.001456672

1 14

0.04267924 0.07942394 0.1066206 0.1221778 0.1259875 0.1196949 0.1060976 0.08840803 0.06959097 0.05192086 0.03680436 0.02483118 0.01596707 0.009795895

# primary-lower-upper-bounds-and-probs-of-infectious-days-relative-to-symptom-onset-day

1

1 16

1.00 1.00 1.00 1.00 1.00 1.00 0.80 0.80 0.60 0.60 0.40 0.40 0.30 0.30 0.10 0.10

# number-of-c2p-transmission-probabilities

1

# number-of-p2p-transmission-probabilities

5

# number-of-pathogenicity-groups

0

# number-of-preseason-immunity-groups

1

# number-of-time-independent-covariates

0

# number-of-time-dependent-covariates

0

# illness-period-as-a-time-dependent-covariate-for-infectivity

0:

# covariates-affecting-susceptibility-for-c2p-transmission

0:

# covariates-affecting-susceptibility-for-p2p-transmission

0:

# covariates-affecting-infectiousness-for-p2p-transmission

0:

# interactions-for-p2p-transmission

0:

# covariates-affecting-pathogenicity

0:

# covariates-affecting-preseason-immunity

0:

# equal-parameters

7:

1:1

1:2

1:3

1:4

1:5

1:6

1:7

# fixed-parameters

0:

# perform-simulation

0

# proportion-with-ambiguity-about-preimmunity-and-escape-status

0

# optimization-choice

1

# converge-criteria

1:

b 1.0e-3

p0 1.0e-3

p1 1.0e-3

p2 1.0e-3

p3 1.0e-3

p4 1.0e-3

u0 1.0e-3

# initial-estimates

1:3

b0 0.088

b1 0.018

b2 0.065

b3 0.057

b4 0.015

p0 0.027

u0 0.1

b0 0.084

b1 0.081

b2 0.014

b3 0.04

b4 0.012

p0 0.019

u0 0.5

b0 0.087

b1 0.049

b2 0.01

b3 0.061

b4 0.02

p0 0.016

u0 0.7

# search-bounds

1:

b0 1.0e-12 0.1

b1 1.0e-12 0.1

b2 1.0e-12 0.1

b3 1.0e-12 0.1

b4 1.0e-12 0.1

p0 1.0e-12 0.1

u0 1.0e-12 0.99

# perform-EM-algorithm

1

# min-number-of-possible-status-to-use-mcem

10000

# use-community-specific-weighting

0

# number-of-base-mcmc-samples

100

# number-of-burnin-mcmc-samples

100

# number-of-burnin-mcmc-iterations

10

# number-of-samplings-for-mc-error

0

# use-bootstrap-for-mc-error

0

# do-not-calculate-average-variance-for-mc-error

0

# check-missingness

0

# check-mixing

0

# check-runtime

0

# relative-infectivity-of-asymptomatic-case-for-simulation

0

# relative-infectivity-of-asymptomatic-case-for-estimation

1

# members-share-common-contact-history-within-communities

1

# automatically-generate-c2p-contact-file

1

# automatically-generate-p2p-contact-file

0

# use-c2p-offset

0

# use-p2p-offset

0

# adjust-for-selection-bias

1

# adjust-for-right-censoring

1

# prefix-index-cases

1

# epidemic-duration-for-calculating-CPI

10.4

# effective-lower-upper-bounds-of-infectious-days-relative-to-symptom-onset-day

0:

# covariates-for-calculating-SAR-provided

0:

# multiplier-for-calculating-R0

5:

3.6 3.02

0 0

0 0

0 0

0 0

0 0

3.6 3.02

0 0

0 0

0 0

0 0

0 0

3.6 3.02

0 0

0 0

0 0

0 0

0 0

3.6 3.02

0 0

0 0

0 0

0 0

0 0

3.6 3.02

# serial-division-of-epidemic-for-calculating-time-varying-R0

0:

168 335 168

336 419 84

420 503 84

504 587 84

588 709 122

# goodness-of-fit

1

# perform-statistical-test

0

# do-not-estimate-variance

0

# do-not-output-estimates

0

# simplify-output

0

# simplify-output-SAR

0

# simplify-output-R0

0

# run-transtat-silently

0

# write-error-log

0

impute.dat

13 0 0 0 -1 -1 1 345 382

21 0 0 0 -1 -1 1 348 382

32 0 0 0 -1 -1 1 367 390

62 0 0 0 -1 -1 1 383 414

64 0 0 0 -1 -1 1 383 439

130 0 0 0 -1 -1 1 464 494

245 0 0 0 -1 -1 1 334 389

249 0 0 0 -1 -1 1 338 387

295 0 0 0 -1 -1 1 355 382

298 0 0 0 -1 -1 1 355 388

299 0 0 0 -1 -1 1 355 389

313 0 0 0 -1 -1 1 353 384

315 0 0 0 -1 -1 1 353 384

321 0 0 0 -1 -1 1 352 383

322 0 0 0 -1 -1 1 352 383

324 0 0 0 -1 -1 1 352 383

330 0 0 0 -1 -1 1 352 384

334 0 0 0 -1 -1 1 355 389

339 0 0 0 -1 -1 1 360 384

361 0 0 0 -1 -1 1 354 383

402 0 0 0 -1 -1 1 359 389

403 0 0 0 -1 -1 1 359 389

413 0 0 0 -1 -1 1 352 402

416 0 0 0 -1 -1 1 362 394

448 0 0 0 -1 -1 1 382 418

474 0 0 0 -1 -1 1 377 410

476 0 0 0 -1 -1 1 370 403

478 0 0 0 -1 -1 1 362 399

483 0 0 0 -1 -1 1 381 412

491 0 0 0 -1 -1 1 376 411

522 0 0 0 -1 -1 1 382 411

528 0 0 0 -1 -1 1 396 426

536 0 0 0 -1 -1 1 397 425

559 0 0 0 -1 -1 1 375 430

561 1 0 0 -1 -1 1 353 430

573 0 0 0 -1 -1 1 389 436

576 0 0 0 -1 -1 1 390 418

593 0 0 0 -1 -1 1 396 425

613 0 0 0 -1 -1 1 396 424

625 0 0 0 -1 -1 1 397 425

650 0 0 0 -1 -1 1 394 422

675 1 1 0 -1 -1 1 367 396

735 0 0 0 -1 -1 1 432 465

761 0 0 0 -1 -1 1 534 564

784 0 0 0 -1 -1 1 550 580

809 1 1 0 -1 -1 1 647 678

811 1 1 0 -1 -1 1 647 678

813 1 1 0 -1 -1 1 648 688

815 1 1 0 -1 -1 1 651 688

816 1 1 0 -1 -1 1 651 688

817 1 1 0 -1 -1 1 651 688

819 1 1 0 -1 -1 1 655 691

820 1 1 0 -1 -1 1 655 690

821 1 1 0 -1 -1 1 655 690

825 1 1 0 -1 -1 1 675 703

826 1 1 0 -1 -1 1 675 703

827 1 1 0 -1 -1 1 675 703

p2p_contact.dat

0 246 298 0 0 0

1 268 304 0 0 0

2 314 344 0 0 0

3 317 382 0 0 0

4 321 347 0 0 0

5 324 348 0 0 0

6 326 382 0 0 0

7 330 352 0 0 0

8 336 359 0 0 0

9 334 387 0 0 0

10 332 390 0 0 0

11 329 374 0 0 0

12 338 390 0 0 0

13 340 392 0 0 0

14 339 397 0 0 0

15 342 403 0 0 0

16 346 422 0 0 0

17 344 374 0 0 0

18 345 368 0 0 0

19 346 405 0 0 0

20 348 383 0 0 0

21 353 409 0 0 0

22 354 375 0 0 0

23 346 414 0 0 0

24 355 439 0 0 0

25 355 380 0 0 0

26 357 408 0 0 0

27 359 390 0 0 0

28 361 418 0 0 0

29 362 418 0 0 0

30 361 426 0 0 0

31 364 423 0 0 0

32 367 424 0 0 0

33 369 423 0 0 0

34 370 426 0 0 0

35 370 398 0 0 0

36 380 452 0 0 0

37 381 438 0 0 0

38 386 415 0 0 0

39 390 455 0 0 0

40 390 455 0 0 0

41 381 460 0 0 0

42 393 429 0 0 0

43 402 457 0 0 0

44 400 470 0 0 0

45 378 436 0 0 0

46 400 468 0 0 0

47 398 470 0 0 0

48 428 494 0 0 0

49 493 557 0 0 0

50 499 566 0 0 0

51 499 566 0 0 0

52 501 567 0 0 0

53 501 567 0 0 0

54 500 560 0 0 0

55 497 569 0 0 0

56 506 557 0 0 0

57 506 579 0 0 0

58 512 582 0 0 0

59 514 543 0 0 0

60 513 581 0 0 0

61 520 579 0 0 0

62 519 580 0 0 0

63 540 600 0 0 0

64 510 601 0 0 0

65 653 688 0 0 0

66 654 689 0 0 0

67 655 690 0 0 0

68 655 691 0 0 0

69 655 691 0 0 0

70 650 684 0 0 0

71 659 689 0 0 0

72 234 338 0 0 0

73 276 341 0 0 0

74 274 362 0 0 0

75 281 348 0 0 0

76 282 354 0 0 0

77 284 344 0 0 0

78 284 346 0 0 0

79 302 368 0 0 0

80 306 394 0 0 0

81 306 387 0 0 0

82 307 387 0 0 0

83 311 394 0 0 0

84 311 374 0 0 0

85 314 373 0 0 0

86 315 376 0 0 0

87 317 387 0 0 0

88 318 377 0 0 0

89 318 411 0 0 0

90 318 382 0 0 0

91 319 389 0 0 0

92 307 389 0 0 0

93 320 384 0 0 0

94 320 396 0 0 0

95 318 383 0 0 0

96 321 396 0 0 0

97 323 384 0 0 0

98 321 397 0 0 0

99 320 384 0 0 0

100 323 419 0 0 0

101 318 408 0 0 0

102 319 391 0 0 0

103 322 383 0 0 0

104 321 388 0 0 0

105 325 384 0 0 0

106 326 348 0 0 0

107 327 394 0 0 0

108 325 410 0 0 0

109 324 395 0 0 0

110 323 388 0 0 0

111 324 383 0 0 0

112 325 353 0 0 0

113 326 403 0 0 0

114 325 389 0 0 0

115 320 394 0 0 0

116 331 402 0 0 0

117 330 394 0 0 0

118 325 389 0 0 0

119 330 389 0 0 0

120 336 389 0 0 0

121 336 394 0 0 0

122 332 410 0 0 0

123 331 419 0 0 0

124 335 410 0 0 0

125 338 360 0 0 0

126 338 398 0 0 0

127 339 418 0 0 0

128 331 403 0 0 0

129 335 408 0 0 0

130 339 412 0 0 0

131 337 394 0 0 0

132 335 390 0 0 0

133 339 394 0 0 0

134 337 410 0 0 0

135 338 403 0 0 0

136 338 399 0 0 0

137 341 412 0 0 0

138 342 404 0 0 0

139 342 403 0 0 0

140 341 411 0 0 0

141 337 416 0 0 0

142 338 418 0 0 0

143 340 397 0 0 0

144 339 401 0 0 0

145 339 431 0 0 0

146 340 417 0 0 0

147 346 404 0 0 0

148 343 411 0 0 0

149 341 405 0 0 0

150 345 426 0 0 0

151 345 417 0 0 0

152 342 425 0 0 0

153 341 411 0 0 0

154 347 418 0 0 0

155 352 405 0 0 0

156 351 410 0 0 0

157 353 430 0 0 0

158 355 412 0 0 0

159 354 444 0 0 0

160 354 418 0 0 0

161 355 408 0 0 0

162 355 417 0 0 0

163 355 433 0 0 0

164 357 411 0 0 0

165 358 417 0 0 0

166 358 426 0 0 0

167 359 411 0 0 0

168 361 416 0 0 0

169 359 412 0 0 0

170 360 432 0 0 0

171 358 424 0 0 0

172 360 425 0 0 0

173 361 418 0 0 0

174 362 425 0 0 0

175 361 425 0 0 0

176 362 425 0 0 0

177 366 422 0 0 0

178 364 429 0 0 0

179 365 430 0 0 0

180 364 422 0 0 0

181 365 433 0 0 0

182 363 429 0 0 0

183 367 437 0 0 0

184 356 431 0 0 0

185 359 438 0 0 0

186 367 431 0 0 0

187 363 424 0 0 0

188 367 437 0 0 0

189 373 433 0 0 0

190 372 431 0 0 0

191 371 438 0 0 0

192 371 437 0 0 0

193 371 429 0 0 0

194 373 405 0 0 0

195 374 438 0 0 0

196 368 433 0 0 0

197 375 430 0 0 0

198 346 443 0 0 0

199 378 438 0 0 0

200 381 440 0 0 0

201 386 445 0 0 0

202 388 447 0 0 0

203 394 453 0 0 0

204 396 468 0 0 0

205 466 538 0 0 0

206 487 548 0 0 0

207 488 552 0 0 0

208 494 552 0 0 0

209 496 572 0 0 0

210 499 564 0 0 0

211 500 559 0 0 0

212 500 559 0 0 0

213 502 576 0 0 0

214 503 564 0 0 0

215 506 569 0 0 0

216 515 551 0 0 0

217 515 580 0 0 0

218 517 577 0 0 0

219 509 579 0 0 0

220 429 579 0 0 0

221 535 594 0 0 0

222 544 611 0 0 0

223 644 704 0 0 0

224 647 678 0 0 0

225 648 688 0 0 0

226 651 688 0 0 0

227 655 691 0 0 0

228 675 703 0 0 0

229 675 703 0 0 0

pop.dat

0 0 0 1 1 266 0 -1 1 0 0 1 0

1 0 0 1 1 298 0 -1 0 0 0 1 0

2 1 0 1 1 288 0 -1 1 0 0 1 0

3 1 1 0 0 -1 1 304 0 0 0 1 0

4 1 0 0 0 -1 1 304 0 0 0 1 0

5 1 1 0 0 -1 1 303 0 0 0 1 0

6 1 0 1 1 304 0 -1 0 0 0 1 0

7 1 0 1 1 304 0 -1 0 0 0 1 0

8 2 0 1 1 334 0 -1 1 0 0 1 0

9 2 0 0 0 -1 1 340 0 0 0 1 0

10 2 0 0 0 -1 1 344 0 0 0 1 0

11 2 0 0 0 -1 1 343 0 0 0 1 0

12 3 0 1 1 337 0 -1 1 0 0 1 0

13 3 0 1 0 -1 0 -1 0 0 0 1 0

14 4 0 1 1 341 0 -1 1 0 0 1 0

15 4 0 0 0 -1 1 347 0 0 0 1 0

16 4 0 1 1 347 0 -1 0 0 0 1 0

17 4 0 0 0 -1 1 347 0 0 0 1 0

18 5 0 1 1 344 0 -1 1 0 0 1 0

19 5 1 0 0 -1 1 348 0 0 0 1 0

20 6 0 1 1 346 0 -1 1 0 0 1 0

21 6 0 1 0 -1 0 -1 0 0 0 1 0

22 7 0 1 1 350 0 -1 1 0 0 1 0

23 7 0 1 1 352 0 -1 0 0 0 1 0

24 8 0 1 1 356 0 -1 1 0 0 1 0

25 8 0 1 1 358 0 -1 0 0 0 1 0

26 9 0 1 1 354 0 -1 1 0 0 1 0

27 9 1 0 0 -1 1 362 0 0 0 1 0

28 9 1 0 0 -1 1 362 0 0 0 1 0

29 9 0 0 0 -1 1 362 0 0 0 1 0

30 9 0 0 0 -1 1 362 0 0 0 1 0

31 10 0 1 1 352 0 -1 1 0 0 1 0

32 10 0 1 0 -1 0 -1 0 0 0 1 0

33 11 0 1 1 349 0 -1 1 0 0 1 0

34 11 0 1 1 374 0 -1 0 0 0 1 0

35 12 0 1 1 358 0 -1 1 0 0 1 0

36 12 0 1 1 360 0 -1 0 0 0 1 0

37 13 0 1 1 360 0 -1 1 0 0 1 0

38 13 1 0 0 -1 1 392 0 0 0 1 0

39 14 0 1 1 359 0 -1 1 0 0 1 0

40 14 1 0 0 -1 1 397 0 0 0 1 0

41 15 0 1 1 362 0 -1 1 0 0 1 0

42 15 1 0 0 -1 1 403 0 0 0 1 0

43 16 0 1 1 366 0 -1 1 0 0 1 0

44 16 1 0 0 -1 1 422 0 0 0 1 0

45 17 0 1 1 364 0 -1 1 0 0 1 0

46 17 0 0 0 -1 1 374 0 0 0 1 0

47 18 0 1 1 365 0 -1 1 0 0 1 0

48 18 0 0 0 -1 1 368 0 0 0 1 0

49 19 0 1 1 366 0 -1 1 0 0 1 0

50 19 0 1 1 374 0 -1 0 0 0 1 0

51 19 1 0 0 -1 1 374 0 0 0 1 0

52 20 0 1 1 368 0 -1 1 0 0 1 0

53 20 1 0 0 -1 1 383 0 0 0 1 0

54 21 0 1 1 373 0 -1 1 0 0 1 0

55 21 1 0 0 -1 1 409 0 0 0 1 0

56 21 1 0 0 -1 1 409 0 0 0 1 0

57 21 1 0 0 -1 1 409 0 0 0 1 0

58 22 0 1 1 374 0 -1 1 0 0 1 0

59 22 1 0 0 -1 1 375 0 0 0 1 0

60 23 0 1 1 366 0 -1 1 0 0 1 0

61 23 0 1 1 368 0 -1 0 0 0 1 0

62 23 0 1 0 -1 0 -1 0 0 0 1 0

63 24 0 1 1 375 0 -1 1 0 0 1 0

64 24 0 1 0 -1 0 -1 0 0 0 1 0

65 24 0 0 0 -1 1 439 0 0 0 1 0

66 24 1 0 0 -1 1 439 0 0 0 1 0

67 25 0 1 1 375 0 -1 1 0 0 1 0

68 25 1 0 0 -1 1 380 0 0 0 1 0

69 26 0 1 1 377 0 -1 1 0 0 1 0

70 26 0 1 1 380 0 -1 0 0 0 1 0

71 27 0 1 1 379 0 -1 1 0 0 1 0

72 27 0 0 0 -1 1 390 0 0 0 1 0

73 28 0 1 1 381 0 -1 1 0 0 1 0

74 28 1 0 0 -1 1 418 0 0 0 1 0

75 28 1 0 0 -1 1 418 0 0 0 1 0

76 28 1 0 0 -1 1 418 0 0 0 1 0

77 29 0 1 1 382 0 -1 1 0 0 1 0

78 29 1 0 0 -1 1 418 0 0 0 1 0

79 29 0 0 0 -1 1 418 0 0 0 1 0

80 29 1 0 0 -1 1 418 0 0 0 1 0

81 30 0 1 1 381 0 -1 1 0 0 1 0

82 30 1 0 0 -1 1 426 0 0 0 1 0

83 30 0 1 1 390 0 -1 0 0 0 1 0

84 31 0 1 1 384 0 -1 1 0 0 1 0

85 31 1 0 0 -1 1 423 0 0 0 1 0

86 31 1 0 0 -1 1 423 0 0 0 1 0

87 32 0 1 1 387 0 -1 1 0 0 1 0

88 32 1 0 0 -1 1 424 0 0 0 1 0

89 32 0 0 0 -1 1 424 0 0 0 1 0

90 32 1 0 0 -1 1 424 0 0 0 1 0

91 32 1 0 0 -1 1 424 0 0 0 1 0

92 33 0 1 1 389 0 -1 1 0 0 1 0

93 33 0 0 0 -1 1 423 0 0 0 1 0

94 34 0 1 1 390 0 -1 1 0 0 1 0

95 34 1 0 0 -1 1 426 0 0 0 1 0

96 34 1 0 0 -1 1 426 0 0 0 1 0

97 35 0 1 1 390 0 -1 1 0 0 1 0

98 35 1 0 0 -1 1 398 0 0 0 1 0

99 35 1 0 0 -1 1 398 0 0 0 1 0

100 36 0 1 1 400 0 -1 1 0 0 1 0

101 36 1 0 0 -1 1 452 0 0 0 1 0

102 36 1 0 0 -1 1 452 0 0 0 1 0

103 37 0 1 1 401 0 -1 1 0 0 1 0

104 37 0 0 0 -1 1 438 0 0 0 1 0

105 38 0 1 1 406 0 -1 1 0 0 1 0

106 38 1 0 0 -1 1 415 0 0 0 1 0

107 38 1 0 0 -1 1 415 0 0 0 1 0

108 38 1 0 0 -1 1 415 0 0 0 1 0

109 38 1 0 0 -1 1 415 0 0 0 1 0

110 39 0 1 1 410 0 -1 1 0 0 1 0

111 39 1 0 0 -1 1 455 0 0 0 1 0

112 40 0 1 1 410 0 -1 1 0 0 1 0

113 40 1 0 0 -1 1 424 0 0 0 1 0

114 41 0 1 1 401 0 -1 1 0 0 1 0

115 41 0 0 0 -1 1 447 0 0 0 1 0

116 42 0 1 1 413 0 -1 1 0 0 1 0

117 42 0 0 0 -1 1 429 0 0 0 1 0

118 43 0 1 1 422 0 -1 1 0 0 1 0

119 43 1 0 0 -1 1 457 0 0 0 1 0

120 44 0 1 1 420 0 -1 1 0 0 1 0

121 44 0 0 0 -1 1 470 0 0 0 1 0

122 45 0 1 1 398 0 -1 1 0 0 1 0

123 45 0 0 0 -1 1 436 0 0 0 1 0

124 46 0 1 1 420 0 -1 1 0 0 1 0

125 46 1 0 0 -1 1 468 0 0 0 1 0

126 46 0 0 0 -1 1 468 0 0 0 1 0

127 47 0 1 1 418 0 -1 1 0 0 1 0

128 47 0 0 0 -1 1 470 0 0 0 1 0

129 48 0 1 1 448 0 -1 1 0 0 1 0

130 48 0 1 0 -1 0 -1 0 0 0 1 0

131 49 0 1 1 513 0 -1 1 0 0 1 0

132 49 1 0 0 -1 1 557 0 0 0 1 0

133 49 1 0 0 -1 1 557 0 0 0 1 0

134 50 0 1 1 519 0 -1 1 0 0 1 0

135 50 1 0 0 -1 1 566 0 0 0 1 0

136 50 1 0 0 -1 1 566 0 0 0 1 0

137 50 1 0 0 -1 1 566 0 0 0 1 0

138 50 1 0 0 -1 1 566 0 0 0 1 0

139 50 1 0 0 -1 1 566 0 0 0 1 0

140 50 0 0 0 -1 1 566 0 0 0 1 0

141 51 0 1 1 519 0 -1 1 0 0 1 0

142 51 1 0 0 -1 1 566 0 0 0 1 0

143 51 0 0 0 -1 1 566 0 0 0 1 0

144 52 0 1 1 521 0 -1 1 0 0 1 0

145 52 0 0 0 -1 1 567 0 0 0 1 0

146 53 0 1 1 521 0 -1 1 0 0 1 0

147 53 0 0 0 -1 1 567 0 0 0 1 0

148 53 1 0 0 -1 1 567 0 0 0 1 0

149 53 1 0 0 -1 1 567 0 0 0 1 0

150 54 0 1 1 520 0 -1 1 0 0 1 0

151 54 1 0 0 -1 1 560 0 0 0 1 0

152 55 0 1 1 517 0 -1 1 0 0 1 0

153 55 1 0 0 -1 1 569 0 0 0 1 0

154 55 1 0 0 -1 1 569 0 0 0 1 0

155 55 1 0 0 -1 1 569 0 0 0 1 0

156 56 0 1 1 526 0 -1 1 0 0 1 0

157 56 1 0 0 -1 1 557 0 0 0 1 0

158 56 1 0 0 -1 1 557 0 0 0 1 0

159 57 0 1 1 526 0 -1 1 0 0 1 0

160 57 1 0 0 -1 1 579 0 0 0 1 0

161 57 0 0 0 -1 1 579 0 0 0 1 0

162 58 0 1 1 532 0 -1 1 0 0 1 0

163 58 1 0 0 -1 1 572 0 0 0 1 0

164 58 1 0 0 -1 1 582 0 0 0 1 0

165 58 1 0 0 -1 1 572 0 0 0 1 0

166 59 0 1 1 534 0 -1 1 0 0 1 0

167 59 1 0 0 -1 1 543 0 0 0 1 0

168 59 1 0 0 -1 1 543 0 0 0 1 0

169 59 1 0 0 -1 1 543 0 0 0 1 0

170 60 0 1 1 533 0 -1 1 0 0 1 0

171 60 0 0 0 -1 1 581 0 0 0 1 0

172 61 0 1 1 540 0 -1 1 0 0 1 0

173 61 1 0 0 -1 1 579 0 0 0 1 0

174 61 1 0 0 -1 1 579 0 0 0 1 0

175 62 0 1 1 539 0 -1 1 0 0 1 0

176 62 1 0 0 -1 1 549 0 0 0 1 0

177 63 0 1 1 560 0 -1 1 0 0 1 0

178 63 1 0 0 -1 1 600 0 0 0 1 0

179 64 0 1 1 530 0 -1 1 0 0 1 0

180 64 1 0 0 -1 1 601 0 0 0 1 0

181 64 1 0 0 -1 1 601 0 0 0 1 0

182 65 0 1 1 673 0 -1 1 0 0 1 0

183 65 0 0 0 -1 1 688 0 0 0 1 0

184 65 0 0 0 -1 1 688 0 0 0 1 0

185 65 1 0 0 -1 1 681 0 0 0 1 0

186 66 0 1 1 674 0 -1 1 0 0 1 0

187 66 1 0 0 -1 1 682 0 0 0 1 0

188 66 0 0 0 -1 1 689 0 0 0 1 0

189 66 0 0 0 -1 1 689 0 0 0 1 0

190 67 0 1 1 675 0 -1 1 0 0 1 0

191 67 1 0 0 -1 1 683 0 0 0 1 0

192 67 0 0 0 -1 1 690 0 0 0 1 0

193 67 0 0 0 -1 1 690 0 0 0 1 0

194 68 0 1 1 675 0 -1 1 0 0 1 0

195 68 1 0 0 -1 1 684 0 0 0 1 0

196 68 0 0 0 -1 1 691 0 0 0 1 0

197 69 0 1 1 675 0 -1 1 0 0 1 0

198 69 1 0 0 -1 1 684 0 0 0 1 0

199 69 0 0 0 -1 1 691 0 0 0 1 0

200 70 0 1 1 670 0 -1 1 0 0 1 0

201 70 1 0 0 -1 1 684 0 0 0 1 0

202 71 0 1 1 679 0 -1 1 0 0 1 0

203 71 1 0 0 -1 1 689 0 0 0 1 0

204 71 0 0 0 -1 1 689 0 0 0 1 0

205 72 0 1 1 254 0 -1 1 0 0 1 0

206 72 0 0 0 -1 1 338 0 0 0 1 0

207 72 0 0 0 -1 1 338 0 0 0 1 0

208 72 0 0 0 -1 1 338 0 0 0 1 0

209 72 0 0 0 -1 1 338 0 0 0 1 0

210 72 0 0 0 -1 1 306 0 0 0 1 0

211 73 0 1 1 296 0 -1 1 0 0 1 0

212 73 0 1 1 304 0 -1 0 0 0 1 0

213 73 0 0 0 -1 1 341 0 0 0 1 0

214 73 0 1 1 305 0 -1 0 0 0 1 0

215 74 0 1 1 294 0 -1 1 0 0 1 0

216 74 0 1 1 304 0 -1 0 0 0 1 0

217 74 1 0 0 -1 1 304 0 0 0 1 0

218 74 0 1 1 304 0 -1 0 0 0 1 0

219 75 0 1 1 301 0 -1 1 0 0 1 0

220 75 0 0 0 -1 1 348 0 0 0 1 0

221 76 0 1 1 302 0 -1 1 0 0 1 0

222 76 0 0 0 -1 1 354 0 0 0 1 0

223 76 0 0 0 -1 1 319 0 0 0 1 0

224 76 0 1 1 312 0 -1 0 0 0 1 0

225 76 0 0 0 -1 1 323 0 0 0 1 0

226 76 0 0 0 -1 1 319 0 0 0 1 0

227 77 0 1 1 304 0 -1 1 0 0 1 0

228 77 1 0 0 -1 1 314 0 0 0 1 0

229 77 1 0 0 -1 1 344 0 0 0 1 0

230 77 1 0 0 -1 1 314 0 0 0 1 0

231 77 1 0 0 -1 1 314 0 0 0 1 0

232 78 0 1 1 304 0 -1 1 0 0 1 0

233 78 0 0 0 -1 1 346 0 0 0 1 0

234 78 0 0 0 -1 1 346 0 0 0 1 0

235 78 0 1 1 313 0 -1 0 0 0 1 0

236 78 0 0 0 -1 1 346 0 0 0 1 0

237 79 0 1 1 322 0 -1 1 0 0 1 0

238 79 1 0 0 -1 1 325 0 0 0 1 0

239 79 0 0 0 -1 1 368 0 0 0 1 0

240 79 0 0 0 -1 1 368 0 0 0 1 0

241 79 0 0 0 -1 1 368 0 0 0 1 0

242 80 0 1 1 326 0 -1 1 0 0 1 0

243 80 0 1 1 320 0 -1 1 0 0 1 0

244 80 0 1 1 320 0 -1 1 0 0 1 0

245 80 0 1 0 -1 0 -1 0 0 0 1 0

246 80 0 0 0 -1 1 394 0 0 0 1 0

247 80 0 1 1 333 0 -1 0 0 0 1 0

248 81 0 1 1 326 0 -1 1 0 0 1 0

249 81 0 1 0 -1 0 -1 0 0 0 1 0

250 81 0 1 1 328 0 -1 0 0 0 1 0

251 81 0 1 1 331 0 -1 0 0 0 1 0

252 82 0 1 1 327 0 -1 1 0 0 1 0

253 82 0 1 1 331 0 -1 0 0 0 1 0

254 82 0 0 0 -1 1 387 0 0 0 1 0

255 83 0 1 1 331 0 -1 1 0 0 1 0

256 83 0 1 1 332 0 -1 0 0 0 1 0

257 83 0 0 0 -1 1 363 0 0 0 1 0

258 83 0 1 1 330 0 -1 1 0 0 1 0

259 83 0 0 0 -1 1 334 0 0 0 1 0

260 84 0 1 1 331 0 -1 1 0 0 1 0

261 84 0 0 0 -1 1 352 0 0 0 1 0

262 84 0 0 0 -1 1 352 0 0 0 1 0

263 84 0 0 0 -1 1 352 0 0 0 1 0

264 84 0 0 0 -1 1 335 0 0 0 1 0

265 85 0 1 1 334 0 -1 1 0 0 1 0

266 85 1 0 0 -1 1 335 0 0 0 1 0

267 85 0 1 1 335 0 -1 0 0 0 1 0

268 85 0 0 0 -1 1 335 0 0 0 1 0

269 85 0 1 1 334 0 -1 1 0 0 1 0

270 85 0 1 1 334 0 -1 1 0 0 1 0

271 85 0 1 1 335 0 -1 0 0 0 1 0

272 85 0 0 0 -1 1 346 0 0 0 1 0

273 86 0 1 1 335 0 -1 1 0 0 1 0

274 86 0 1 1 340 0 -1 0 0 0 1 0

275 87 0 1 1 337 0 -1 1 0 0 1 0

276 87 0 0 0 -1 1 346 0 0 0 1 0

277 87 0 0 0 -1 1 339 0 0 0 1 0

278 88 0 1 1 338 0 -1 1 0 0 1 0

279 88 0 1 1 338 0 -1 1 0 0 1 0

280 88 0 1 1 337 0 -1 1 0 0 1 0

281 88 1 0 0 -1 1 340 0 0 0 1 0

282 88 1 0 0 -1 1 340 0 0 0 1 0

283 89 0 1 1 338 0 -1 1 0 0 1 0

284 89 1 0 0 -1 1 340 0 0 0 1 0

285 90 0 1 1 338 0 -1 1 0 0 1 0

286 90 1 0 0 -1 1 382 0 0 0 1 0

287 90 1 0 0 -1 1 382 0 0 0 1 0

288 90 0 1 1 352 0 -1 0 0 0 1 0

289 90 1 0 0 -1 1 382 0 0 0 1 0

290 90 1 0 0 -1 1 382 0 0 0 1 0

291 90 1 0 0 -1 1 382 0 0 0 1 0

292 90 0 1 1 341 0 -1 0 0 0 1 0

293 90 1 0 0 -1 1 382 0 0 0 1 0

294 90 1 0 0 -1 1 382 0 0 0 1 0

295 90 0 1 0 -1 0 -1 0 0 0 1 0

296 91 0 1 1 339 0 -1 1 0 0 1 0

297 91 0 1 1 342 0 -1 0 0 0 1 0

298 91 0 1 0 -1 0 -1 0 0 0 1 0

299 91 0 1 0 -1 0 -1 0 0 0 1 0

300 92 0 1 1 327 0 -1 1 0 0 1 0

301 92 0 1 1 340 0 -1 0 0 0 1 0

302 92 0 0 0 -1 1 342 0 0 0 1 0

303 92 0 1 1 342 0 -1 0 0 0 1 0

304 93 0 1 1 340 0 -1 1 0 0 1 0

305 93 0 0 0 -1 1 353 0 0 0 1 0

306 93 0 0 0 -1 1 384 0 0 0 1 0

307 93 0 0 0 -1 1 384 0 0 0 1 0

308 93 0 1 1 346 0 -1 0 0 0 1 0

309 93 0 1 1 344 0 -1 0 0 0 1 0

310 93 1 0 0 -1 1 384 0 0 0 1 0

311 93 0 1 1 344 0 -1 0 0 0 1 0

312 93 0 1 1 343 0 -1 0 0 0 1 0

313 93 0 1 0 -1 0 -1 0 0 0 1 0

314 93 0 1 1 344 0 -1 0 0 0 1 0

315 93 0 1 0 -1 0 -1 0 0 0 1 0

316 94 0 1 1 340 0 -1 1 0 0 1 0

317 94 0 1 1 345 0 -1 0 0 0 1 0

318 94 0 0 0 -1 1 389 0 0 0 1 0

319 95 0 1 1 338 0 -1 1 0 0 1 0

320 95 1 0 0 -1 1 383 0 0 0 1 0

321 95 0 1 0 -1 0 -1 0 0 0 1 0

322 95 0 1 0 -1 0 -1 0 0 0 1 0

323 95 0 1 1 352 0 -1 0 0 0 1 0

324 95 0 1 0 -1 0 -1 0 0 0 1 0

325 96 0 1 1 341 0 -1 1 0 0 1 0

326 96 1 0 0 -1 1 396 0 0 0 1 0

327 97 0 1 1 343 0 -1 1 0 0 1 0

328 97 1 0 0 -1 1 383 0 0 0 1 0

329 97 0 1 1 345 0 -1 0 0 0 1 0

330 97 0 1 0 -1 0 -1 0 0 0 1 0

331 97 0 1 1 352 0 -1 0 0 0 1 0

332 97 0 0 0 -1 1 383 0 0 0 1 0

333 98 0 1 1 341 0 -1 1 0 0 1 0

334 98 0 1 0 -1 0 -1 0 0 0 1 0

335 98 0 0 0 -1 1 389 0 0 0 1 0

336 99 0 1 1 340 0 -1 1 0 0 1 0

337 99 0 1 1 346 0 -1 0 0 0 1 0

338 99 0 1 1 346 0 -1 0 0 0 1 0

339 99 0 1 0 -1 0 -1 0 0 0 1 0

340 100 0 1 1 343 0 -1 1 0 0 1 0

341 100 0 0 0 -1 1 391 0 0 0 1 0

342 101 0 1 1 338 0 -1 1 0 0 1 0

343 101 1 0 0 -1 1 347 0 0 0 1 0

344 101 1 0 0 -1 1 408 0 0 0 1 0

345 101 0 1 1 347 0 -1 0 0 0 1 0

346 102 0 1 1 339 0 -1 1 0 0 1 0

347 102 0 1 1 339 0 -1 1 0 0 1 0

348 102 0 0 0 -1 1 391 0 0 0 1 0

349 103 0 1 1 342 0 -1 1 0 0 1 0

350 103 1 0 0 -1 1 383 0 0 0 1 0

351 103 1 0 0 -1 1 383 0 0 0 1 0

352 103 0 0 0 -1 1 383 0 0 0 1 0

353 103 0 1 1 344 0 -1 0 0 0 1 0

354 103 1 0 0 -1 1 383 0 0 0 1 0

355 103 1 0 0 -1 1 383 0 0 0 1 0

356 103 0 1 1 352 0 -1 0 0 0 1 0

357 103 1 0 0 -1 1 383 0 0 0 1 0

358 103 1 0 0 -1 1 383 0 0 0 1 0

359 103 0 0 0 -1 1 383 0 0 0 1 0

360 103 0 1 1 353 0 -1 0 0 0 1 0

361 103 0 1 0 -1 0 -1 0 0 0 1 0

362 104 0 1 1 341 0 -1 1 0 0 1 0

363 104 1 0 0 -1 1 388 0 0 0 1 0

364 104 1 0 0 -1 1 388 0 0 0 1 0

365 104 1 0 0 -1 1 388 0 0 0 1 0

366 105 0 1 1 345 0 -1 1 0 0 1 0

367 105 0 1 1 346 0 -1 0 0 0 1 0

368 105 0 1 1 353 0 -1 0 0 0 1 0

369 105 0 1 1 352 0 -1 0 0 0 1 0

370 106 0 1 1 346 0 -1 1 0 0 1 0

371 106 0 0 0 -1 1 348 0 0 0 1 0

372 107 0 1 1 347 0 -1 1 0 0 1 0

373 107 0 1 1 361 0 -1 0 0 0 1 0

374 107 1 0 0 -1 1 394 0 0 0 1 0

375 107 0 1 1 362 0 -1 0 0 0 1 0

376 108 0 1 1 345 0 -1 1 0 0 1 0

377 108 0 0 0 -1 1 363 0 0 0 1 0

378 108 0 1 1 352 0 -1 0 0 0 1 0

379 109 0 1 1 344 0 -1 1 0 0 1 0

380 109 0 1 1 360 0 -1 0 0 0 1 0

381 109 1 0 0 -1 1 395 0 0 0 1 0

382 110 0 1 1 343 0 -1 1 0 0 1 0

383 110 0 0 0 -1 1 348 0 0 0 1 0

384 110 1 0 0 -1 1 388 0 0 0 1 0

385 110 0 1 1 347 0 -1 0 0 0 1 0

386 111 0 1 1 344 0 -1 1 0 0 1 0

387 111 1 0 0 -1 1 383 0 0 0 1 0

388 111 0 1 1 347 0 -1 0 0 0 1 0

389 111 1 0 0 -1 1 383 0 0 0 1 0

390 112 0 1 1 345 0 -1 1 0 0 1 0

391 112 1 0 0 -1 1 353 0 0 0 1 0

392 113 0 1 1 346 0 -1 1 0 0 1 0

393 113 0 1 1 361 0 -1 0 0 0 1 0

394 113 0 1 1 353 0 -1 0 0 0 1 0

395 113 0 1 1 361 0 -1 0 0 0 1 0

396 113 1 0 0 -1 1 403 0 0 0 1 0

397 113 1 0 0 -1 1 403 0 0 0 1 0

398 114 0 1 1 345 0 -1 1 0 0 1 0

399 114 0 1 1 359 0 -1 0 0 0 1 0

400 114 0 1 1 359 0 -1 0 0 0 1 0

401 114 1 0 0 -1 1 389 0 0 0 1 0

402 114 0 1 0 -1 0 -1 0 0 0 1 0

403 114 0 1 0 -1 0 -1 0 0 0 1 0

404 114 0 0 0 -1 1 360 0 0 0 1 0

405 114 0 1 1 348 0 -1 0 0 0 1 0

406 114 1 0 0 -1 1 352 0 0 0 1 0

407 115 0 1 1 340 0 -1 1 0 0 1 0

408 115 0 1 1 350 0 -1 0 0 0 1 0

409 115 1 0 0 -1 1 352 0 0 0 1 0

410 115 0 1 1 352 0 -1 0 0 0 1 0

411 115 1 0 0 -1 1 394 0 0 0 1 0

412 116 0 1 1 351 0 -1 1 0 0 1 0

413 116 0 1 0 -1 0 -1 0 0 0 1 0

414 117 0 1 1 350 0 -1 1 0 0 1 0

415 117 0 0 0 -1 1 394 0 0 0 1 0

416 117 0 1 0 -1 0 -1 0 0 0 1 0

417 118 0 1 1 345 0 -1 1 0 0 1 0

418 118 0 1 1 353 0 -1 0 0 0 1 0

419 118 0 1 1 355 0 -1 0 0 0 1 0

420 119 0 1 1 350 0 -1 1 0 0 1 0

421 119 1 0 0 -1 1 362 0 0 0 1 0

422 120 0 1 1 356 0 -1 1 0 0 1 0

423 120 1 0 0 -1 1 389 0 0 0 1 0

424 121 0 1 1 356 0 -1 1 0 0 1 0

425 121 1 0 0 -1 1 394 0 0 0 1 0

426 122 0 1 1 352 0 -1 1 0 0 1 0

427 122 1 0 0 -1 1 410 0 0 0 1 0

428 123 0 1 1 351 0 -1 1 0 0 1 0

429 123 1 0 0 -1 1 419 0 0 0 1 0

430 123 1 0 0 -1 1 419 0 0 0 1 0

431 124 0 1 1 355 0 -1 1 0 0 1 0

432 124 0 1 1 360 0 -1 0 0 0 1 0

433 124 0 1 1 367 0 -1 0 0 0 1 0

434 124 0 1 1 357 0 -1 0 0 0 1 0

435 125 0 1 1 358 0 -1 1 0 0 1 0

436 125 1 0 0 -1 1 360 0 0 0 1 0

437 125 0 0 0 -1 1 360 0 0 0 1 0

438 125 1 0 0 -1 1 360 0 0 0 1 0

439 126 0 1 1 358 0 -1 1 0 0 1 0

440 126 0 1 1 356 0 -1 1 0 0 1 0

441 126 1 0 0 -1 1 360 0 0 0 1 0

442 126 0 1 1 357 0 -1 1 0 0 1 0

443 126 0 1 1 360 0 -1 0 0 0 1 0

444 127 0 1 1 359 0 -1 1 0 0 1 0

445 127 1 0 0 -1 1 417 0 0 0 1 0

446 127 0 1 1 357 0 -1 1 0 0 1 0

447 127 0 0 0 -1 1 418 0 0 0 1 0

448 127 0 1 0 -1 0 -1 0 0 0 1 0

449 128 0 1 1 351 0 -1 1 0 0 1 0

450 128 0 1 1 357 0 -1 0 0 0 1 0

451 128 0 1 1 358 0 -1 0 0 0 1 0

452 128 1 0 0 -1 1 403 0 0 0 1 0

453 129 0 1 1 355 0 -1 1 0 0 1 0

454 129 0 1 1 358 0 -1 0 0 0 1 0

455 129 1 0 0 -1 1 408 0 0 0 1 0

456 129 0 1 1 356 0 -1 0 0 0 1 0

457 130 0 1 1 359 0 -1 1 0 0 1 0

458 130 1 0 0 -1 1 412 0 0 0 1 0

459 130 1 0 0 -1 1 412 0 0 0 1 0

460 130 1 0 0 -1 1 412 0 0 0 1 0

461 130 0 1 1 368 0 -1 0 0 0 1 0

462 131 0 1 1 357 0 -1 1 0 0 1 0

463 131 1 0 0 -1 1 394 0 0 0 1 0

464 132 0 1 1 355 0 -1 1 0 0 1 0

465 132 1 0 0 -1 1 361 0 0 0 1 0

466 133 0 1 1 359 0 -1 1 0 0 1 0

467 133 0 1 1 358 0 -1 1 0 0 1 0

468 133 0 1 1 360 0 -1 0 0 0 1 0

469 134 0 1 1 357 0 -1 1 0 0 1 0

470 134 1 0 0 -1 1 410 0 0 0 1 0

471 134 0 1 1 363 0 -1 0 0 0 1 0

472 134 1 0 0 -1 1 410 0 0 0 1 0

473 134 0 1 1 363 0 -1 0 0 0 1 0

474 134 0 1 0 -1 0 -1 0 0 0 1 0

475 135 0 1 1 358 0 -1 1 0 0 1 0

476 135 0 1 0 -1 0 -1 0 0 0 1 0

477 136 0 1 1 358 0 -1 1 0 0 1 0

478 136 0 1 0 -1 0 -1 0 0 0 1 0

479 136 1 0 0 -1 1 399 0 0 0 1 0

480 136 0 1 1 358 0 -1 1 0 0 1 0

481 136 0 1 1 362 0 -1 0 0 0 1 0

482 137 0 1 1 361 0 -1 1 0 0 1 0

483 137 0 1 0 -1 0 -1 0 0 0 1 0

484 138 0 1 1 362 0 -1 1 0 0 1 0

485 138 1 0 0 -1 1 404 0 0 0 1 0

486 138 0 1 1 373 0 -1 0 0 0 1 0

487 139 0 1 1 362 0 -1 1 0 0 1 0

488 139 0 1 1 382 0 -1 0 0 0 1 0

489 139 1 0 0 -1 1 403 0 0 0 1 0

490 140 0 1 1 361 0 -1 1 0 0 1 0

491 140 0 1 0 -1 0 -1 0 0 0 1 0

492 141 0 1 1 357 0 -1 1 0 0 1 0

493 141 0 0 0 -1 1 416 0 0 0 1 0

494 141 1 0 0 -1 1 416 0 0 0 1 0

495 141 0 1 1 367 0 -1 0 0 0 1 0

496 142 0 1 1 358 0 -1 1 0 0 1 0

497 142 1 0 0 -1 1 418 0 0 0 1 0

498 142 0 0 0 -1 1 418 0 0 0 1 0

499 142 1 0 0 -1 1 418 0 0 0 1 0

500 142 1 0 0 -1 1 418 0 0 0 1 0

501 142 1 0 0 -1 1 418 0 0 0 1 0

502 143 0 1 1 360 0 -1 1 0 0 1 0

503 143 1 0 0 -1 1 397 0 0 0 1 0

504 144 0 1 1 359 0 -1 1 0 0 1 0

505 144 1 0 0 -1 1 370 0 0 0 1 0

506 144 0 1 1 366 0 -1 0 0 0 1 0

507 145 0 1 1 359 0 -1 1 0 0 1 0

508 145 0 1 1 382 0 -1 0 0 0 1 0

509 145 0 1 1 380 0 -1 0 0 0 1 0

510 145 0 1 1 387 0 -1 0 0 0 1 0

511 145 0 1 1 388 0 -1 0 0 0 1 0

512 146 0 1 1 360 0 -1 1 0 0 1 0

513 146 1 0 0 -1 1 417 0 0 0 1 0

514 146 1 0 0 -1 1 417 0 0 0 1 0

515 146 1 0 0 -1 1 417 0 0 0 1 0

516 146 1 0 0 -1 1 417 0 0 0 1 0

517 146 0 0 0 -1 1 417 0 0 0 1 0

518 146 0 0 0 -1 1 417 0 0 0 1 0

519 147 0 1 1 366 0 -1 1 0 0 1 0

520 147 0 0 0 -1 1 404 0 0 0 1 0

521 148 0 1 1 363 0 -1 1 0 0 1 0

522 148 0 1 0 -1 0 -1 0 0 0 1 0

523 149 0 1 1 361 0 -1 1 0 0 1 0

524 149 0 1 1 374 0 -1 0 0 0 1 0

525 149 0 1 1 367 0 -1 0 0 0 1 0

526 150 0 1 1 365 0 -1 1 0 0 1 0

527 150 1 0 0 -1 1 368 0 0 0 1 0

528 150 0 1 0 -1 0 -1 0 0 0 1 0

529 150 0 1 1 382 0 -1 0 0 0 1 0

530 151 0 1 1 365 0 -1 1 0 0 1 0

531 151 0 0 0 -1 1 417 0 0 0 1 0

532 152 0 1 1 362 0 -1 1 0 0 1 0

533 152 0 1 1 360 0 -1 1 0 0 1 0

534 152 1 0 0 -1 1 425 0 0 0 1 0

535 152 0 1 1 367 0 -1 0 0 0 1 0

536 152 0 1 0 -1 0 -1 0 0 0 1 0

537 152 0 1 1 383 0 -1 0 0 0 1 0

538 153 0 1 1 361 0 -1 1 0 0 1 0

539 153 0 1 1 379 0 -1 0 0 0 1 0

540 153 1 0 0 -1 1 411 0 0 0 1 0

541 153 1 0 0 -1 1 411 0 0 0 1 0

542 153 1 0 0 -1 1 411 0 0 0 1 0

543 154 0 1 1 367 0 -1 1 0 0 1 0

544 154 0 1 1 385 0 -1 0 0 0 1 0

545 154 0 1 1 371 0 -1 0 0 0 1 0

546 154 0 1 1 372 0 -1 0 0 0 1 0

547 154 0 1 1 373 0 -1 0 0 0 1 0

548 154 0 1 1 371 0 -1 0 0 0 1 0

549 154 0 1 1 370 0 -1 0 0 0 1 0

550 155 0 1 1 372 0 -1 1 0 0 1 0

551 155 0 1 1 375 0 -1 0 0 0 1 0

552 156 0 1 1 371 0 -1 1 0 0 1 0

553 156 1 0 0 -1 1 375 0 0 0 1 0

554 156 1 0 0 -1 1 410 0 0 0 1 0

555 156 1 0 0 -1 1 410 0 0 0 1 0

556 156 1 0 0 -1 1 410 0 0 0 1 0

557 157 0 1 1 373 0 -1 1 0 0 1 0

558 157 0 1 1 368 0 -1 1 0 0 1 0

559 157 0 1 0 -1 0 -1 0 0 0 1 0

560 157 0 1 1 387 0 -1 0 0 0 1 0

561 157 0 0 0 -1 1 430 0 0 0 1 0

562 157 0 1 1 389 0 -1 0 0 0 1 0

563 158 0 1 1 375 0 -1 1 0 0 1 0

564 158 1 0 0 -1 1 412 0 0 0 1 0

565 158 0 1 1 379 0 -1 0 0 0 1 0

566 158 0 1 1 375 0 -1 1 0 0 1 0

567 158 0 1 1 382 0 -1 0 0 0 1 0

568 159 0 1 1 374 0 -1 1 0 0 1 0

569 159 1 0 0 -1 1 444 0 0 0 1 0

570 159 1 0 0 -1 1 436 0 0 0 1 0

571 159 1 0 0 -1 1 436 0 0 0 1 0

572 159 0 0 0 -1 1 436 0 0 0 1 0

573 159 0 1 0 -1 0 -1 0 0 0 1 0

574 160 0 1 1 374 0 -1 1 0 0 1 0

575 160 1 0 0 -1 1 418 0 0 0 1 0

576 160 0 1 0 -1 0 -1 0 0 0 1 0

577 161 0 1 1 375 0 -1 1 0 0 1 0

578 161 1 0 0 -1 1 408 0 0 0 1 0

579 161 1 0 0 -1 1 408 0 0 0 1 0

580 162 0 1 1 375 0 -1 1 0 0 1 0

581 162 0 1 1 382 0 -1 0 0 0 1 0

582 163 0 1 1 375 0 -1 1 0 0 1 0

583 163 1 0 0 -1 1 429 0 0 0 1 0

584 163 0 1 1 380 0 -1 0 0 0 1 0

585 163 1 0 0 -1 1 433 0 0 0 1 0

586 163 0 0 0 -1 1 429 0 0 0 1 0

587 164 0 1 1 377 0 -1 1 0 0 1 0

588 164 1 0 0 -1 1 411 0 0 0 1 0

589 165 0 1 1 378 0 -1 1 0 0 1 0

590 165 1 0 0 -1 1 417 0 0 0 1 0

591 165 0 1 1 381 0 -1 0 0 0 1 0

592 166 0 1 1 378 0 -1 1 0 0 1 0

593 166 0 1 0 -1 0 -1 0 0 0 1 0

594 166 1 0 0 -1 1 426 0 0 0 1 0

595 166 0 0 0 -1 1 426 0 0 0 1 0

596 167 0 1 1 379 0 -1 1 0 0 1 0

597 167 1 0 0 -1 1 411 0 0 0 1 0

598 167 1 0 0 -1 1 411 0 0 0 1 0

599 168 0 1 1 381 0 -1 1 0 0 1 0

600 168 0 1 1 382 0 -1 0 0 0 1 0

601 168 0 0 0 -1 1 416 0 0 0 1 0

602 169 0 1 1 379 0 -1 1 0 0 1 0

603 169 0 1 1 381 0 -1 0 0 0 1 0

604 170 0 1 1 380 0 -1 1 0 0 1 0

605 170 1 0 0 -1 1 432 0 0 0 1 0

606 170 1 0 0 -1 1 432 0 0 0 1 0

607 170 1 0 0 -1 1 432 0 0 0 1 0

608 170 0 0 0 -1 1 432 0 0 0 1 0

609 170 0 1 1 390 0 -1 0 0 0 1 0

610 170 0 1 1 390 0 -1 0 0 0 1 0

611 171 0 1 1 378 0 -1 1 0 0 1 0

612 171 1 0 0 -1 1 424 0 0 0 1 0

613 171 0 1 0 -1 0 -1 0 0 0 1 0

614 171 1 0 0 -1 1 424 0 0 0 1 0

615 172 0 1 1 380 0 -1 1 0 0 1 0

616 172 0 1 1 393 0 -1 0 0 0 1 0

617 172 1 0 0 -1 1 389 0 0 0 1 0

618 172 1 0 0 -1 1 389 0 0 0 1 0

619 173 0 1 1 381 0 -1 1 0 0 1 0

620 173 1 0 0 -1 1 418 0 0 0 1 0

621 174 0 1 1 382 0 -1 1 0 0 1 0

622 174 1 0 0 -1 1 425 0 0 0 1 0

623 174 0 0 0 -1 1 425 0 0 0 1 0

624 174 0 0 0 -1 1 425 0 0 0 1 0

625 174 0 1 0 -1 0 -1 0 0 0 1 0

626 175 0 1 1 381 0 -1 1 0 0 1 0

627 175 1 0 0 -1 1 425 0 0 0 1 0

628 175 0 1 1 390 0 -1 0 0 0 1 0

629 175 1 0 0 -1 1 425 0 0 0 1 0

630 175 1 0 0 -1 1 425 0 0 0 1 0

631 176 0 1 1 382 0 -1 1 0 0 1 0

632 176 1 0 0 -1 1 425 0 0 0 1 0

633 176 1 0 0 -1 1 425 0 0 0 1 0

634 176 0 0 0 -1 1 425 0 0 0 1 0

635 177 0 1 1 386 0 -1 1 0 0 1 0

636 177 1 0 0 -1 1 387 0 0 0 1 0

637 178 0 1 1 384 0 -1 1 0 0 1 0

638 178 1 0 0 -1 1 429 0 0 0 1 0

639 178 0 0 0 -1 1 429 0 0 0 1 0

640 178 1 0 0 -1 1 429 0 0 0 1 0

641 179 0 1 1 385 0 -1 1 0 0 1 0

642 179 0 1 1 383 0 -1 1 0 0 1 0

643 179 0 0 0 -1 1 430 0 0 0 1 0

644 179 0 0 0 -1 1 430 0 0 0 1 0

645 179 1 0 0 -1 1 394 0 0 0 1 0

646 179 1 0 0 -1 1 394 0 0 0 1 0

647 179 0 0 0 -1 1 401 0 0 0 1 0

648 179 1 0 0 -1 1 401 0 0 0 1 0

649 180 0 1 1 384 0 -1 1 0 0 1 0

650 180 0 1 0 -1 0 -1 0 0 0 1 0

651 180 1 0 0 -1 1 394 0 0 0 1 0

652 180 0 0 0 -1 1 394 0 0 0 1 0

653 181 0 1 1 385 0 -1 1 0 0 1 0

654 181 0 0 0 -1 1 431 0 0 0 1 0

655 181 0 0 0 -1 1 431 0 0 0 1 0

656 181 0 0 0 -1 1 433 0 0 0 1 0

657 182 0 1 1 383 0 -1 1 0 0 1 0

658 182 0 0 0 -1 1 429 0 0 0 1 0

659 182 1 0 0 -1 1 429 0 0 0 1 0

660 183 0 1 1 387 0 -1 1 0 0 1 0

661 183 0 0 0 -1 1 437 0 0 0 1 0

662 183 1 0 0 -1 1 437 0 0 0 1 0

663 183 0 1 1 395 0 -1 0 0 0 1 0

664 184 0 1 1 376 0 -1 1 0 0 1 0

665 184 0 0 0 -1 1 431 0 0 0 1 0

666 184 1 0 0 -1 1 431 0 0 0 1 0

667 184 0 0 0 -1 1 431 0 0 0 1 0

668 185 0 1 1 379 0 -1 1 0 0 1 0

669 185 1 0 0 -1 1 438 0 0 0 1 0

670 185 1 0 0 -1 1 397 0 0 0 1 0

671 185 0 0 0 -1 1 438 0 0 0 1 0

672 186 0 1 1 387 0 -1 1 0 0 1 0

673 186 1 0 0 -1 1 431 0 0 0 1 0

674 186 0 0 0 -1 1 431 0 0 0 1 0

675 186 0 0 0 -1 1 396 0 0 0 1 0

676 187 0 1 1 383 0 -1 1 0 0 1 0

677 187 1 0 0 -1 1 424 0 0 0 1 0

678 187 1 0 0 -1 1 424 0 0 0 1 0

679 188 0 1 1 387 0 -1 1 0 0 1 0

680 188 0 0 0 -1 1 437 0 0 0 1 0

681 188 1 0 0 -1 1 437 0 0 0 1 0

682 188 0 0 0 -1 1 437 0 0 0 1 0

683 189 0 1 1 393 0 -1 1 0 0 1 0

684 189 1 0 0 -1 1 433 0 0 0 1 0

685 189 1 0 0 -1 1 433 0 0 0 1 0

686 190 0 1 1 392 0 -1 1 0 0 1 0

687 190 1 0 0 -1 1 431 0 0 0 1 0

688 190 1 0 0 -1 1 431 0 0 0 1 0

689 191 0 1 1 391 0 -1 1 0 0 1 0

690 191 1 0 0 -1 1 438 0 0 0 1 0

691 191 1 0 0 -1 1 438 0 0 0 1 0

692 191 1 0 0 -1 1 438 0 0 0 1 0

693 191 0 0 0 -1 1 438 0 0 0 1 0

694 192 0 1 1 391 0 -1 1 0 0 1 0

695 192 1 0 0 -1 1 429 0 0 0 1 0

696 192 1 0 0 -1 1 437 0 0 0 1 0

697 193 0 1 1 391 0 -1 1 0 0 1 0

698 193 1 0 0 -1 1 429 0 0 0 1 0

699 193 1 0 0 -1 1 429 0 0 0 1 0

700 193 1 0 0 -1 1 429 0 0 0 1 0

701 193 1 0 0 -1 1 429 0 0 0 1 0

702 194 0 1 1 393 0 -1 1 0 0 1 0

703 194 0 0 0 -1 1 405 0 0 0 1 0

704 194 1 0 0 -1 1 405 0 0 0 1 0

705 194 1 0 0 -1 1 405 0 0 0 1 0

706 195 0 1 1 394 0 -1 1 0 0 1 0

707 195 1 0 0 -1 1 438 0 0 0 1 0

708 195 1 0 0 -1 1 402 0 0 0 1 0

709 195 0 1 1 402 0 -1 0 0 0 1 0

710 195 0 0 0 -1 1 438 0 0 0 1 0

711 196 0 1 1 388 0 -1 1 0 0 1 0

712 196 1 0 0 -1 1 433 0 0 0 1 0

713 197 0 1 1 395 0 -1 1 0 0 1 0

714 197 1 0 0 -1 1 430 0 0 0 1 0

715 197 1 0 0 -1 1 430 0 0 0 1 0

716 198 0 1 1 366 0 -1 1 0 0 1 0

717 198 1 0 0 -1 1 443 0 0 0 1 0

718 198 0 0 0 -1 1 443 0 0 0 1 0

719 199 0 1 1 398 0 -1 1 0 0 1 0

720 199 1 0 0 -1 1 408 0 0 0 1 0

721 199 1 0 0 -1 1 438 0 0 0 1 0

722 200 0 1 1 401 0 -1 1 0 0 1 0

723 200 1 0 0 -1 1 440 0 0 0 1 0

724 201 0 1 1 406 0 -1 1 0 0 1 0

725 201 1 0 0 -1 1 445 0 0 0 1 0

726 201 1 0 0 -1 1 445 0 0 0 1 0

727 201 1 0 0 -1 1 445 0 0 0 1 0

728 202 0 1 1 408 0 -1 1 0 0 1 0

729 202 1 0 0 -1 1 447 0 0 0 1 0

730 203 0 1 1 414 0 -1 1 0 0 1 0

731 203 1 0 0 -1 1 453 0 0 0 1 0

732 203 1 0 0 -1 1 453 0 0 0 1 0

733 204 0 1 1 416 0 -1 1 0 0 1 0

734 204 1 0 0 -1 1 468 0 0 0 1 0

735 204 0 1 0 -1 0 -1 0 0 0 1 0

736 204 1 0 0 -1 1 465 0 0 0 1 0

737 205 0 1 1 486 0 -1 1 0 0 1 0

738 205 0 1 1 508 0 -1 0 0 0 1 0

739 205 0 1 1 508 0 -1 0 0 0 1 0

740 205 0 1 1 501 0 -1 0 0 0 1 0

741 206 0 1 1 507 0 -1 1 0 0 1 0

742 206 1 0 0 -1 1 548 0 0 0 1 0

743 206 1 0 0 -1 1 548 0 0 0 1 0

744 206 0 1 1 516 0 -1 0 0 0 1 0

745 206 1 0 0 -1 1 548 0 0 0 1 0

746 207 0 1 1 508 0 -1 1 0 0 1 0

747 207 1 0 0 -1 1 552 0 0 0 1 0

748 207 1 0 0 -1 1 552 0 0 0 1 0

749 208 0 1 1 514 0 -1 1 0 0 1 0

750 208 1 0 0 -1 1 552 0 0 0 1 0

751 209 0 1 1 516 0 -1 1 0 0 1 0

752 209 1 0 0 -1 1 524 0 0 0 1 0

753 209 1 0 0 -1 1 572 0 0 0 1 0

754 209 1 0 0 -1 1 572 0 0 0 1 0

755 209 1 0 0 -1 1 572 0 0 0 1 0

756 209 0 0 0 -1 1 572 0 0 0 1 0

757 209 0 1 1 521 0 -1 0 0 0 1 0

758 209 1 0 0 -1 1 572 0 0 0 1 0

759 210 0 1 1 519 0 -1 1 0 0 1 0

760 210 1 0 0 -1 1 564 0 0 0 1 0

761 210 0 1 0 -1 0 -1 0 0 0 1 0

762 210 0 0 0 -1 1 564 0 0 0 1 0

763 210 1 0 0 -1 1 564 0 0 0 1 0

764 211 0 1 1 520 0 -1 1 0 0 1 0

765 211 1 0 0 -1 1 559 0 0 0 1 0

766 211 1 0 0 -1 1 559 0 0 0 1 0

767 212 0 1 1 520 0 -1 1 0 0 1 0

768 212 1 0 0 -1 1 559 0 0 0 1 0

769 213 0 1 1 522 0 -1 1 0 0 1 0

770 213 0 0 0 -1 1 576 0 0 0 1 0

771 213 1 0 0 -1 1 576 0 0 0 1 0

772 213 0 1 1 531 0 -1 0 0 0 1 0

773 214 0 1 1 523 0 -1 1 0 0 1 0

774 214 1 0 0 -1 1 564 0 0 0 1 0

775 214 1 0 0 -1 1 564 0 0 0 1 0

776 214 1 0 0 -1 1 564 0 0 0 1 0

777 215 0 1 1 526 0 -1 1 0 0 1 0

778 215 1 0 0 -1 1 569 0 0 0 1 0

779 215 1 0 0 -1 1 541 0 0 0 1 0

780 216 0 1 1 535 0 -1 1 0 0 1 0

781 216 1 0 0 -1 1 551 0 0 0 1 0

782 217 0 1 1 535 0 -1 1 0 0 1 0

783 217 1 0 0 -1 1 580 0 0 0 1 0

784 217 0 1 0 -1 0 -1 0 0 0 1 0

785 217 0 0 0 -1 1 580 0 0 0 1 0

786 218 0 1 1 537 0 -1 1 0 0 1 0

787 218 1 0 0 -1 1 577 0 0 0 1 0

788 219 0 1 1 529 0 -1 1 0 0 1 0

789 219 1 0 0 -1 1 579 0 0 0 1 0

790 219 1 0 0 -1 1 579 0 0 0 1 0

791 219 1 0 0 -1 1 579 0 0 0 1 0

792 220 0 1 1 449 0 -1 1 0 0 1 0

793 220 1 0 0 -1 1 579 0 0 0 1 0

794 220 1 0 0 -1 1 579 0 0 0 1 0

795 221 0 1 1 555 0 -1 1 0 0 1 0

796 221 1 0 0 -1 1 594 0 0 0 1 0

797 222 0 1 1 564 0 -1 1 0 0 1 0

798 222 1 0 0 -1 1 611 0 0 0 1 0

799 222 0 0 0 -1 1 611 0 0 0 1 0

800 222 1 0 0 -1 1 611 0 0 0 1 0

801 222 1 0 0 -1 1 611 0 0 0 1 0

802 222 1 0 0 -1 1 611 0 0 0 1 0

803 223 0 1 1 664 0 -1 1 0 0 1 0

804 223 1 0 0 -1 1 674 0 0 0 1 0

805 223 1 0 0 -1 1 674 0 0 0 1 0

806 223 1 0 0 -1 1 704 0 0 0 1 0

807 223 1 0 0 -1 1 704 0 0 0 1 0

808 224 0 1 1 667 0 -1 1 0 0 1 0

809 224 0 0 0 -1 1 678 0 0 0 1 0

810 224 0 1 1 676 0 -1 0 0 0 1 0

811 224 0 0 0 -1 1 678 0 0 0 1 0

812 225 0 1 1 668 0 -1 1 0 0 1 0

813 225 0 0 0 -1 1 688 0 0 0 1 0

814 226 0 1 1 671 0 -1 1 0 0 1 0

815 226 0 0 0 -1 1 688 0 0 0 1 0

816 226 0 0 0 -1 1 688 0 0 0 1 0

817 226 0 0 0 -1 1 688 0 0 0 1 0

818 227 0 1 1 675 0 -1 1 0 0 1 0

819 227 0 0 0 -1 1 691 0 0 0 1 0

820 227 0 0 0 -1 1 690 0 0 0 1 0

821 227 0 0 0 -1 1 690 0 0 0 1 0

822 228 0 1 1 695 0 -1 1 0 0 1 0

823 228 0 1 1 700 0 -1 0 0 0 1 0

824 229 0 1 1 695 0 -1 1 0 0 1 0

825 229 0 0 0 -1 1 703 0 0 0 1 0

826 229 0 0 0 -1 1 703 0 0 0 1 0

827 229 0 0 0 -1 1 703 0 0 0 1 0

**Section 3. Output files from the analyses of the household transmission studies in Burkina Faso and Madagascar**

**3a. Burkina Faso**

# Estimates of b

1.693933e-02, 6.649502e-03, 7.816400e-03, 3.632035e-02

# Estimates of p

3.565228e-02, 2.269447e-02, 1.003686e-02, 1.187967e-01

# Estimates of u

# Estimates of q

4.245501e-01, 7.080933e-02, 2.947961e-01, 5.656092e-01

# Estimates of odds ratios for c2p exposure

# Estimates of odds ratios for p2p exposure

# Estimates of odds ratios for pathogenicity

# Estimates of odds ratios for pre-immunity level

# Estimates of CPI

1.570473e-01, 5.701800e-02, 7.547120e-02, 3.092405e-01

# Estimates of SAR

3.136152e-01, 1.668904e-01, 9.088735e-02, 6.761873e-01

# Estimates of R0

#Log-likelihood

-116.86022248

#Covariance Matrix Estimates

4.421588e-05 -7.854094e-05 -5.897781e-06

-7.854094e-05 5.150388e-04 3.893396e-05

-5.897781e-06 3.893396e-05 5.013961e-03

#Covariance Matrix Estimates for logit(b), logit(p) and log(OR)

1.594501e-01 -1.371824e-01 -1.449691e-03

-1.371824e-01 4.357105e-01 4.635228e-03

-1.449691e-03 4.635228e-03 8.400556e-02

**3b. Madagascar, a single p_r_**

# Estimates of b

7.243455e-03, 1.371960e-03, 4.994886e-03, 1.049360e-02

# Estimates of p

3.130363e-02, 4.506981e-03, 2.357917e-02, 4.145116e-02

# Estimates of u

# Estimates of q

4.830473e-01, 2.083138e-02, 4.424209e-01, 5.238991e-01

# Estimates of odds ratios for c2p exposure

# Estimates of odds ratios for p2p exposure

# Estimates of odds ratios for pathogenicity

# Estimates of odds ratios for pre-immunity level

# Estimates of CPI

7.011853e-02, 1.285068e-02, 4.884098e-02, 1.001169e-01

# Estimates of SAR

2.809411e-01, 3.459349e-02, 2.183282e-01, 3.533924e-01

# Estimates of R0

1.01138805, 0.50385679, 0.38093906, 2.68522160

0.00000000, 0.00000000, -nan, -nan

0.00000000, 0.00000000, -nan, -nan

0.00000000, 0.00000000, -nan, -nan

0.00000000, 0.00000000, -nan, -nan

#Log-likelihood

-1195.05990908

#Covariance Matrix Estimates

1.882274e-06 -3.365791e-06 1.161154e-08

-3.365791e-06 2.031287e-05 1.308379e-07

1.161154e-08 1.308379e-07 4.339464e-04

#Covariance Matrix Estimates for logit(b), logit(p) and log(OR)

3.640038e-02 -1.543534e-02 6.466373e-06

-1.543534e-02 2.209056e-02 1.727869e-05

6.466373e-06 1.727869e-05 6.959133e-03

**3c. Madagascar, five p_r_’s**

# Estimates of b

7.081927e-03, 1.376033e-03, 4.836815e-03, 1.035831e-02

# Estimates of p

1.832277e-02, 8.461816e-03, 7.368249e-03, 4.482808e-02

3.730559e-02, 5.457310e-03, 2.796534e-02, 4.960622e-02

1.661747e-02, 2.314725e-02, 1.051113e-03, 2.134535e-01

1.567300e-02, 1.357694e-02, 2.829197e-03, 8.202782e-02

1.110183e-02, 1.586332e-02, 6.606997e-04, 1.601097e-01

# Estimates of u

# Estimates of q

4.829871e-01, 2.083467e-02, 4.423547e-01, 5.238457e-01

# Estimates of odds ratios for c2p exposure

# Estimates of odds ratios for p2p exposure

# Estimates of odds ratios for pathogenicity

# Estimates of odds ratios for pre-immunity level

# Estimates of CPI

6.860445e-02, 1.290772e-02, 4.732885e-02, 9.888582e-02

# Estimates of SAR

1.746955e-01, 7.373761e-02, 7.208279e-02, 3.657973e-01

3.256747e-01, 3.948223e-02, 2.534704e-01, 4.072273e-01

1.597114e-01, 2.050794e-01, 9.419656e-03, 7.916234e-01

1.513051e-01, 1.213965e-01, 2.719044e-02, 5.320858e-01

1.095137e-01, 1.482607e-01, 6.209099e-03, 7.076661e-01

# Estimates of R0

0.62890368, 0.40327715, 0.17895797, 2.21012699

1.17242881, 0.58353740, 0.44199918, 3.10993631

0.57496113, 0.78873263, 0.03907845, 8.45940132

0.54469836, 0.51002984, 0.08691935, 3.41346665

0.39424947, 0.56665360, 0.02356781, 6.59512551

#Log-likelihood

-1189.71467820

#Covariance Matrix Estimates

1.893466e-06 -2.759699e-06 -3.628302e-06 -4.344348e-06 -2.949111e-06 -3.011239e-06 1.472038e-08

-2.759699e-06 7.160233e-05 4.392781e-06 6.213683e-06 4.155086e-06 1.193983e-06 1.779125e-07

-3.628302e-06 4.392781e-06 2.978223e-05 8.637666e-06 5.231592e-06 5.472503e-06 2.669613e-07

-4.344348e-06 6.213683e-06 8.637666e-06 5.357954e-04 -6.552599e-05 1.629902e-05 -4.930267e-07

-2.949111e-06 4.155086e-06 5.231592e-06 -6.552599e-05 1.843333e-04 2.919290e-06 -4.400772e-07

-3.011239e-06 1.193983e-06 5.472503e-06 1.629902e-05 2.919290e-06 2.516448e-04 -1.357442e-06

1.472038e-08 1.779125e-07 2.669613e-07 -4.930267e-07 -4.400772e-07 -1.357442e-06 4.340835e-04

#Covariance Matrix Estimates for logit(b), logit(p) and log(OR)

3.829375e-02 -2.181912e-02 -1.436733e-02 -3.780701e-02 -2.718534e-02 -3.900625e-02 8.383343e-06

-2.181912e-02 2.213133e-01 6.800132e-03 2.113985e-02 1.497369e-02 6.046334e-03 3.961046e-05

-1.436733e-02 6.800132e-03 2.309046e-02 1.471792e-02 9.442348e-03 1.387961e-02 2.976795e-05

-3.780701e-02 2.113985e-02 1.471792e-02 2.006430e+00 -2.599170e-01 9.085060e-02 -1.208220e-04

-2.718534e-02 1.497369e-02 9.442348e-03 -2.599170e-01 7.744989e-01 1.723612e-02 -1.142354e-04

-3.900625e-02 6.046334e-03 1.387961e-02 9.085060e-02 1.723612e-02 2.087832e+00 -4.951517e-04

8.383343e-06 3.961046e-05 2.976795e-05 -1.208220e-04 -1.142354e-04 -4.951517e-04 6.961445e-03

**Section 4. Input files for the calibration of each country’s Susceptible, Infectious, and Recovered (SIR) mathematical model**

**4a. Burkina Faso**

**4a.1. Data files**

Codebook:

time : week since March 8, 2020

Y1 : number of observed laboratory-confirmed symptomatic SARS-CoV-2 cases observed since the last observation time

| **time** | **Y1** |
| --- | --- |
| 1 | 0 |
| 2 | 1 |
| 4 | 0 |
| 5 | 0 |
| 6 | 0 |
| 8 | 0 |
| 9 | 0 |
| 10 | 0 |
| 11 | 1 |
| 12 | 0 |
| 13 | 0 |
| 14 | 1 |
| 15 | 0 |
| 16 | 1 |
| 17 | 1 |
| 18 | 0 |
| 19 | 0 |
| 20 | 2 |
| 21 | 3 |
| 22 | 1 |
| 23 | 1 |
| 24 | 1 |
| 25 | 2 |
| 26 | 11 |
| 27 | 12 |
| 28 | 24 |
| 29 | 61 |
| 30 | 54 |
| 31 | 8 |
| 32 | 3 |
| 33 | 3 |
| 34 | 0 |
| 35 | 0 |
| 36 | 0 |
| 37 | 0 |
| 38 | 0 |
| 39 | 0 |
| 40 | 0 |
| 41 | 0 |
| 42 | 0 |
| 43 | 0 |
| 44 | 0 |
| 45 | 0 |
| 46 | 0 |
| 47 | 1 |
| 48 | 3 |
| 49 | 1 |
| 50 | 0 |
| 51 | 1 |
| 52 | 0 |
| 53 | 0 |
| 54 | 0 |
| 55 | 2 |
| 56 | 0 |
| 57 | 0 |
| 58 | 0 |
| 59 | 1 |
| 60 | 0 |
| 61 | 0 |
| 62 | 2 |
| 63 | 3 |
| 64 | 2 |
| 65 | 1 |
| 66 | 7 |
| 67 | 4 |
| 68 | 4 |
| 69 | 0 |

**4a.2. Model definition**

##The following procedure defines the SIR model

rproc<-Csnippet("

double foi, births,mn_p,var_lgt_p,lgt_p,p_sel,lgt_p_sel, Re,mn_hh_size,min_R0,max_R0, mn_b,var_lgt_b,lgt_b,lgt_b_sel,b_sel, mn_q, var_lgt_q, lgt_q, lgt_q_sel, q_sel, mn_hh_lg_sel, mn_hh_lgt, var_hh_lgt, SAR,n,slp;

double rate[10], trans[10];

//Force of infection, takes the general form of average number of contacts * secondary attack

//rate * temporal forcing function * current prevalence of infectious individuals

//Inputs

//Mean and variance of the natural logarithm of the average number of members

//(including index case) from the household transmission study

mn_hh_lgt = 1.270132;

var_hh_lgt = 0.3373533;

//Household transmission study estimated mean for the probability of transmitting

//SARS-CoV-2 per daily per within-household contact, followed by the variance of the logit of

//the estimate

mn_p = 3.565228e-02;

var_lgt_p = 4.357105e-01;

//Household transmission study estimated mean daily probability of being infected with

//SARS-CoV-2 due to exposure to sources outside of the household, followed by the variance

//of the logit of the estimate

mn_b = 1.693933e-02;

var_lgt_b = 1.594501e-01;

//Household transmission study estimated mean probability of being not susceptible to SARS-

//CoV-2 infection at enrollment into the household transmission study, followed by the

//variance of the logit of the estimate

mn_q = 4.245501e-01;

var_lgt_q = 8.400556e-02;

//For every week, the following code draws a random sample of values for p, b, q, and h from

//the distributions implied by the model inputs above.

lgt_p = log(mn_p/(1-mn_p));

lgt_p_sel = rnorm(lgt_p,sqrt(var_lgt_p));

p_sel = exp(lgt_p_sel)/(1+exp(lgt_p_sel));

lgt_b = log(mn_b/(1-mn_b));

lgt_b_sel = rnorm(lgt_b,sqrt(var_lgt_b));

b_sel = exp(lgt_b_sel)/(1+exp(lgt_b_sel));

lgt_q = log(mn_q/(1-mn_q));

lgt_q_sel = rnorm(lgt_q,sqrt(var_lgt_q));

q_sel = exp(lgt_q_sel)/(1+exp(lgt_q_sel));

mn_hh_lg_sel = rnorm(mn_hh_lgt,sqrt(var_hh_lgt));

mn_hh_size = exp(mn_hh_lg_sel);

//Calculate household secondary attack rate based upon sampled value for p

SAR = (1-pow((1-p_sel),(7/gamma)));

//For the time period during which the household transmission study was conducted, calculate

//the ratio for the overall number of potentially-infectious contacts per day to the number

//among members of the same household. This ratio is multiplied against the sampled value

//for the mean number of household contacts to estimate the average number of susceptible

//contacts experienced by each infectious individual in an entirely susceptible population.

//Here, we assume that all contacts are close in nature (i.e., similar to within-household

//contacts), which leads to the assumption that b represents the daily cumulative probability

//of infection associated with n potentially infectious contacts with probability of transmission

//of p.

n = mn_hh_size*(q_sel*mn_hh_size+(log(1-b_sel) / log(1 - p_sel)))/(q_sel*mn_hh_size);

//R0 is calculated below, under the assumption detailed a few lines up.

//We introduce a temporal forcing function, where the wavelength of the cosine is fixed at

// 2*3.1415 / 37 weeks (defined in the model calibration component of the code. The

//timeline is shifted by 66 weeks so that the week of March 8, 2020 is defined as time 0. The code

//also allows for a shifting of the cosine wave, using the delay input parameter, which was set

//to 12 for Burkina Faso (also defined in the model calibration component of the code).

slp = n*SAR;

Re = slp*2*(0.5*cos((t+66-delay)*wvl)+0.5);

//Define force of infection (foi)

foi = Re*gamma*I/(S+I+R);

//Define rates for movement of individuals in and out of disease states

rate[0] = foi; //force of infection leaving the S state (stochastic)

rate[1] = mu; //natural death/migration rate leaving the S state (set to 0 by assumption)

rate[5] = gamma; // loss of infectious rate (set to 10.4 days / 7 days per week by assumption)

rate[6] = mu; // natural death/migration rate leaving the I state (set to 0 by assumption)

rate[7] = foi*delta; // force of infection among previously infected individuals (stochastic)

rate[8] = mu; // natural death/migration rate leaving the R state (set to 0 by assumption)

// transitions between classes/states

reulermultinom(2,S,&rate[0],dt,&trans[0]);

reulermultinom(2,I,&rate[5],dt,&trans[5]);

reulermultinom(3,R,&rate[7],dt,&trans[7]);

//The population is assumed to be stable in size, so the number of births is equal to the total

//number of deaths/out-migrations (set to 0 by assumption)

births = trans[1]+trans[6]+trans[8];

S += births - trans[0] - trans[1];

I += trans[0] + trans[7] - trans[5] - trans[6];

R += trans[5] - trans[7] - trans[8];

C = trans[0]+trans[7]; // incident infections

A += R; //Tracking state that accumulates the number of individuals who are in the R state

//between observation times. This variable is reset to 0 at each observation time.

Z += 1; //Tracking state that accumulates the number of weeks that occur between observation

//times. A/Z provides an estimate of the mean number of individuals in the R state

//during the period between observation times. This variable is reset to 0 at each

//observation time.

")

##The following procedure defines the Measurement model linking the SIR model to the

##observed data. This code allows for two potential assumptions regarding the distributions of

##Y1 and Gobs1: negative binomial or Poisson (i.e., setting psi’s equal to 0). We chose the

##latter.

dmeas2 <- Csnippet("

double rho,psi;

rho = rho1;

psi = psi1;

double m4, m6,m4_var;

double m1 = rho*C;

double v1 = m1*(1.0-rho+psi*psi*m1);

double tol = 1.0e-18;

//Likelihood only defined for observations during the prospective surveillance period.

if (t > 0) {

if (Y1 > 0.0) {

lik = pnorm(Y1+0.5,m1,sqrt(v1)+tol,1,0)-pnorm(Y1-0.5,m1,sqrt(v1)+tol,1,0)+tol;

} else {

lik = pnorm(Y1+0.5,m1,sqrt(v1)+tol,1,0)+tol;

}

} else {

lik = tol;

}

if (give_log) lik = log(lik);

")

##The following code defines the measurement model for simulations using the calibrated

##model.

rmeas2 <- Csnippet("

double tol = 1.0e-18;

double m1 = rho1*C;

double v1 = m1*(1.0-rho1+psi1*psi1*m1);

Y1 = rnorm(m1,sqrt(v1)+tol);

if (Y1 > 0.0) {

Y1 = nearbyint(Y1);

} else {

Y1 = 0.0;

}

")

##The following function defines the initial state for the SIR model at time 0, i.e., week -66.

##propI represents the initial proportion of the population who are infectious. We assume that

##only 1 individual was infectious in this population at time 0. This assumption is encoded in

##the model calibration section of the code.

rinit <- Csnippet("

double pop = 80000;

double intI = propI;

double intS = 1.0 - intI;

double m = pop/(intS+intI);

S = nearbyint(m*intS);

I = nearbyint(m*intI);

R = 0;

C = 0;

A = 0;

Z = 1; //initially set to 1 to avoid division by 0

")

##This function builds the code object that will be used to calibrate and simulate the model

##the partrans setting defines transformations to be applied to input parameters prior to

##sampling values for simulation-based inference. This helps to improve the calibration

##algorithms performance.

m1<-pomp(data=dat[,!(colnames(dat) %in% c("st","ed"))],t0=-66,

time="time",

rprocess=euler(rproc,delta.t=1),

dmeasure=dmeas2,

rmeasure=rmeas2,

rinit=rinit,

partrans=parameter_trans(

log=c("gamma","mu","psi1","delay"),

logit=c("rho1","delta","propI","wvl")

),

obsnames=c("Y1","Gobs1","N1"),

statenames=c("S","I","R","C","A","Z"),

paramnames=c("gamma","rho1","delta","mu","propI","psi1","decay","wvl","delay"),

accumvars = c("C","A","Z")

)

**4a.3. Model calibration**

Note: The following code calibrates the above model in an iterative fashion. A profile likelihood approach is used to estimate the likelihood profile for each parameter at twenty-five equal spaced intervals across a range of values that can change for each iteration. The initial range of values for each parameter is defined by the user and typically includes the range of all plausible values. For subsequent iterations, the possible range of values for each parameter is defined by the maximum and minimum values for the parameter’s values whose profile likelihood falls within the top 50% of all of the values estimated from a LOESS fit to the accumulated calibration runs for all preceding iterations. For each profile likelihood run, we run ten random replicates for each of the twenty-five locations along the current range for the parameter’s values. By random replicate, we mean that though the value for the parameter being profiled remains fixed, we randomly draw values from the allowed range(s) for all other parameters that we are trying to calibrate.

##Start of code

set.seed(123456)

for(x in 1:4) {

crange<-c(2,3) ##define which of the parameters in the ‘ranges’ list we are planning to profile

vind<-235 ##define the model calibration run’s unique identifier

##define whether or not we want to turn on standard error informed smoothing of the LOESS smoothing procedure for defining each likelihood profile. We chose to not use this functionality.

wind<-0

##Define the range of allowed values for each parameter. In the initial part of the code, the user defines the initial

##allowed range. The first vector defines the minimum and the second the maximum for each parameter. Here is

##where some assumptions are encoded.

if (x==1) {

ranges<-rbind(c(gamma=7/10.4,rho1=0.0,delta=0.0,mu=0,propI=1/80000,psi1=0,decay=0.0,wvl=2*pi/37,delay=12),

c(gamma=7/10.4,rho1=1.0,delta=1.0,mu=0,propI=1/80000,psi1=0,decay=0.0,wvl=2*pi/37,delay=12),deparse.level=0)

}

if(x > 1) {

ranges1<-ranges2[1:2,]

for(c in crange) {

if(ranges[1,c]==ranges2[1,c]) {

ranges1[1,c]<-(ranges2[1,c]+0.5*(ranges2[2,c]-ranges2[1,c]))

if((ranges2[1,c]+0.5*(ranges2[2,c]-ranges2[1,c])) < ranges[2,c]*0.5) {

ranges1[2,c]<-ranges[2,c]*0.5

}

if((ranges2[1,c]+0.5*(ranges2[2,c]-ranges2[1,c])) >= ranges[2,c]*0.5) {

ranges1[2,c]<-ranges[2,c]

}

}

if(ranges[2,c]==ranges2[2,c]) {

ranges1[2,c]<-(ranges2[1,c]+0.5*(ranges2[2,c]-ranges2[1,c]))

if((ranges2[1,c]+0.5*(ranges2[2,c]-ranges2[1,c])) > ranges[2,c]*0.5) {

ranges1[1,c]<-ranges[2,c]*0.5

}

if((ranges2[1,c]+0.5*(ranges2[2,c]-ranges2[1,c])) <= ranges[2,c]*0.5) {

ranges1[1,c]<-ranges[1,c]

}

}

if (ranges1[1,c] > ranges1[2,c]) {

ranges1[1:2,c]<-ranges1[2:1,c]

}

}

ranges<-ranges1

rm(ranges1)

}

cn<-colnames(ranges)

##Start the process of defining the input to facilitate the profile likelihood exercise

starts<-c()

rind<-x

minrind<-rep(1,12)

maxrind<-rep(rind,12)

ccnt<-0

ranges2<-c()

##Here we are starting the looping over the parameters to be profiled.

for(c in crange) {

cn_temp <- c(cn[c],cn[-c])

##The rv[] vector controls how the modified iterative filtering algorithm samples each parameter (using the

##index/order listed in ‘ranges’) during the calibration process. Essentially, if rv[] is set to 0, then the algorithm will

##not try to vary the input value for a parameter.

rv=rep(0.02,ncol(ranges))

rv[c]<-0

rv[1]<-0

##rv[2]<-0

##rv[3]<-0

rv[4]<-0

rv[5]<-0

rv[6]<-0

rv[7]<-0

rv[8]<-0

rv[9]<-0

##This builds the set of start values for the 10 replicates runs for each of the 25 locations along the possible range ##of values for the parameter being profiled.

starts <- profile_design(

t1=seq(

from=ranges[1,c],

to=ranges[2,c],

length=25

),

lower=ranges[1,-c],

upper=ranges[2,-c],

nprof=10

)

colnames(starts)<-cn_temp

start_t<-Sys.time()

coefs<-c()

logliks<-c()

##The following loop estimates the likelihood of the data given the model and the starting parameter values

##presented in each row of the ‘starts’ matrix. Since values for the parameter being profiled are being held

##constant (i.e., the modified iterative filtering algorithm does not change the initial input value), the results of this

##loop are the estimated likelihood for each of the ten replicates of the twenty-five different values for the

##parameter being profiled.

for(i in 1:nrow(starts)) {

start_t_step<-Sys.time()

mif<-try(mif2(m1,params=starts[i,],

Nmif=10,

Np=50,

rw.sd=rw_sd(gamma=rv[1],

rho1=ifelse(time < 1,0,rv[2]),

delta=rv[3],

mu=rv[4],

propI=rv[5],

theta=rv[6],

ehh=rv[7],

psi1=ifelse(time < 1,0,rv[8]),

decay = rv[9],

wvl = rv[10],

delay = rv[11]),

cooling.type = "geometric",cooling.fraction.50=0.5,verbose=F))

coefs<-rbind(coefs,try(coef(mif)),deparse.level=0)

try(mif |> pfilter(Nrep=5,verbose=F) |> logLik() |> logmeanexp(se=TRUE,ess=TRUE)) -> tloglik

logliks<-rbind(logliks,tloglik,deparse.level=0)

rm(mif)

rm(tloglik)

print(paste(cn[c],": ",i,": ",Sys.time()-start_t_step,": ",Sys.time()-start_t))

}

##Writing output to file

write.csv(coefs,paste("profile_",cn[c],"_coef_v",vind,"_",rind,".csv",sep=""))

write.csv(logliks,paste("profile_",cn[c],"_loglik_v",vind,"_",rind,".csv",sep=""))

}

##Fit LOESS curve to all profile likelihood estimates generated up to this point (across the current and preceding

##iterations) for parameter currently being profiled.

minrind<-rep(1,12)

maxrind<-rep(rind,12)

ranges2<-c()

ccnt<-0

for(c in crange ) {

ccnt<-ccnt+1

cn_temp <- c(cn[c],cn[-c],"loglik","loglik.se","loglik.ess")

t0<-c()

for(i in minrind[ccnt]:maxrind[ccnt]) {

if(file.exists(paste("profile_",cn[c],"_coef_v",vind,"_",i,".csv",sep=""))) {

t1<-read.csv(paste("profile_",cn[c],"_coef_v",vind,"_",i,".csv",sep=""))

t2<-read.csv(paste("profile_",cn[c],"_loglik_v",vind,"_",i,".csv",sep=""))

t1a<-which(grepl("Error",t1[,2],fixed=T)==F & grepl("Error",t2[,2],fixed=T)==F)

t1<-t1[t1a,]

t2<-t2[t1a,]

t1<-matrix(as.numeric(unlist(t1)),nrow=nrow(t1))

t2<-matrix(as.numeric(unlist(t2)),nrow=nrow(t2))

t0<-as.data.frame(rbind(t0,cbind(t1[,-1],t2[,-1]),deparse.level=0))

}

}

colnames(t0)<-cn_temp

t0<-t0[apply(t0,1,FUN=function(x) sum(as.numeric(grepl("Error",x,fixed=T)))) == 0,]

##If using standard error informed weighting, then these weights are defined here.

if(wind==1) {

wts<-t0$loglik.ess

} else {

wts<-rep(1,nrow(t0))

}

##The LOESS fit for the likelihood profile, including definition of likelihood ratio based 95% confidence limits

m1_x<-seq(min(t0[,1]),max(t0[,1]),length.out=1.0e6)

m1m <- loess(t0$loglik~t0[,1],model=T,weights=wts)

m1p<-predict(m1m,newdata=m1_x)

m1_xs<-m1_x[m1p >= (max(m1p)-5)]

m1_xs2<-m1_x[m1p >= (max(m1p)-2)]

m1ps<-m1p[m1p >= (max(m1p)-5)]

m1ps2<-m1p[m1p >= (max(m1p)-2)]

m1_xs2<-m1_x[m1p >= (max(m1p) - 0.1*(max(m1p)-min(m1p)))]

ll<-min(m1_xs2)

ul<-max(m1_xs2)

m1_xs2<-m1_x[m1p >= (max(m1p)-2)]

mle<-m1_xs2[m1ps2==max(m1ps2)]

mle_ll<-max(m1ps2)

ranges2<-cbind(ranges2,c(ll,ul,mle))

}

##End of the parameter calibration loop for an iteration

ranges2<-cbind(rep(ranges[1,1],nrow(ranges2)),ranges2[,1:2],rep(ranges[1,4],nrow(ranges2)),rep(ranges[1,5],nrow(ranges2)),rep(ranges[1,6],nrow(ranges2)),rep(ranges[1,7],nrow(ranges2)),rep(ranges[1,8],nrow(ranges2)),rep(ranges[1,9],nrow(ranges2)))

colnames(ranges2)<-cn

##Simulate and plot epidemics using the current model calibration

t3<-dat$time[dat$time>=64]

ranges3<-ranges2[3,]

simout<-c()

i<-0

plot(dat$time,dat$Y1,ylim=c(1,100),xlim=c(0,max(dat$time)),type="p",col=0,xlab="Study Week",ylab="Number of laboratory-confirmed symptomatic SARS-CoV-2 cases")

while(i < 1000) {

t1<-simulate(m1,nsim=1,params=ranges3,format="data.frame",verbose=F,times=dat$time[dat$time >= 64])

if(t1$I[nrow(t1)] >= 0)

{

i<-i+1

if(i %in% c(10*seq(1,100))) {

lines(t1$time,t1$Y1, col="gray")

}

simout<-rbind(simout,cbind(rep(i,nrow(t1)),t1),deparse.level=0)

}

t3<-cbind(t3,t1$A/t1$Z/80000)

}

t2<-aggregate(simout$Y1,by=list(simout$time),mean)

lines(t2$Group.1,t2$x,col="red")

points(dat$time,dat$Y1,ylim=c(1,100),col=1,pch=20)

cbind(t3[t3[,1] < 0,1],rowSums(t3[t3[,1] < 0,2:ncol(t3)])/ncol(t3))

unique(simout[simout[,2]==150 & simout$Y1 > 50,1])

}

## End of the iteration loops

**4b. Madagascar**

**4b.1. Data files**

Codebook:

time : week since March 3, 2020

Y1 : number of observed laboratory-confirmed symptomatic SARS-CoV-2 cases observed since the last observation time

Gobs1 : number febrile individuals enrolled in SETA surveillance who were positive for anti-SARS-CoV-2 IgG by rapid antibody test

N1 : number febrile individuals enrolled in SETA surveillance who were not positive for anti-SARS-CoV-2 IgG by rapid antibody test

| **time** | **Gobs1** | **N1** |
| --- | --- | --- |
| 64 | 1 | 51 |
| 78 | 7 | 93 |
| 95 | 20 | 80 |
| 104 | 17 | 83 |
| 113 | 9 | 75 |
| 123 | 27 | 73 |
| 134 | 42 | 84 |

| **time** | **Y1** |
| --- | --- |
| 137 | 1 |
| 138 | 0 |
| 139 | 0 |
| 141 | 0 |
| 142 | 0 |
| 143 | 0 |
| 144 | 0 |
| 145 | 1 |
| 146 | 0 |
| 147 | 1 |
| 148 | 0 |
| 149 | 0 |
| 150 | 2 |
| 151 | 4 |
| 152 | 8 |
| 153 | 1 |
| 154 | 4 |
| 155 | 9 |
| 156 | 14 |
| 157 | 52 |
| 158 | 48 |
| 159 | 49 |
| 160 | 57 |
| 161 | 33 |
| 162 | 29 |
| 163 | 34 |
| 164 | 28 |
| 165 | 19 |
| 166 | 12 |
| 167 | 10 |
| 168 | 3 |
| 169 | 9 |
| 170 | 0 |
| 171 | 0 |
| 172 | 0 |
| 173 | 3 |
| 174 | 2 |
| 175 | 0 |
| 176 | 0 |
| 177 | 0 |
| 178 | 1 |
| 179 | 0 |
| 180 | 0 |
| 181 | 5 |
| 182 | 2 |
| 183 | 16 |
| 184 | 7 |
| 185 | 12 |
| 186 | 5 |
| 187 | 0 |
| 188 | 1 |
| 189 | 2 |
| 190 | 0 |
| 191 | 0 |
| 192 | 0 |
| 193 | 0 |
| 194 | 0 |
| 195 | 0 |
| 196 | 0 |
| 197 | 0 |
| 198 | 0 |
| 199 | 0 |
| 200 | 0 |
| 201 | 0 |
| 202 | 0 |
| 203 | 0 |
| 204 | 4 |
| 205 | 10 |
| 206 | 3 |
| 207 | 0 |
| 208 | 2 |
| 209 | 1 |
| 210 | 0 |

**4b.2. Model definition**

##The following procedure defines the SIR model

rproc<-Csnippet("

double foi, births,mn_p,var_lgt_p,lgt_p,p_sel,lgt_p_sel, Re,mn_hh_size,min_R0,max_R0, mn_b,var_lgt_b,lgt_b,lgt_b_sel,b_sel, mn_q, var_lgt_q, lgt_q, lgt_q_sel, q_sel, mn_hh_lg_sel, mn_hh_lgt, var_hh_lgt, SAR,n,slp;

double rate[10], trans[10];

//Force of infection, takes the general form of average number of contacts * secondary attack

//rate * temporal forcing function * current prevalence of infectious individuals

//Inputs

//Mean and variance of the natural logarithm of the average number of members

//(including index case) from the household transmission study

mn_hh_lgt = 1.186454;

var_hh_lgt = 0.1788603;

//Household transmission study estimated mean for the probability of transmitting

//SARS-CoV-2 per daily per within-household contact, followed by the variance of the logit of

//the estimate

mn_p = 3.130363e-02;

var_lgt_p = 2.209056e-02;

//Household transmission study estimated mean daily probability of being infected with

//SARS-CoV-2 due to exposure to sources outside of the household, followed by the variance

//of the logit of the estimate

mn_b = 7.243455e-03;

var_lgt_b = 3.640038e-02;

//Household transmission study estimated mean probability of being not susceptible to SARS-

//CoV-2 infection at enrollment into the household transmission study, followed by the

//variance of the logit of the estimate

mn_q = 4.830473e-01;

var_lgt_q = 6.959133e-03;

//For every week, the following code draws a random sample of values for p, b, q, and h from

//the distributions implied by the model inputs above.

lgt_p = log(mn_p/(1-mn_p));

lgt_p_sel = rnorm(lgt_p,sqrt(var_lgt_p));

p_sel = exp(lgt_p_sel)/(1+exp(lgt_p_sel));

lgt_b = log(mn_b/(1-mn_b));

lgt_b_sel = rnorm(lgt_b,sqrt(var_lgt_b));

b_sel = exp(lgt_b_sel)/(1+exp(lgt_b_sel));

lgt_q = log(mn_q/(1-mn_q));

lgt_q_sel = rnorm(lgt_q,sqrt(var_lgt_q));

q_sel = exp(lgt_q_sel)/(1+exp(lgt_q_sel));

mn_hh_lg_sel = rnorm(mn_hh_lgt,sqrt(var_hh_lgt));

mn_hh_size = exp(mn_hh_lg_sel);

//Calculate household secondary attack rate based upon sampled value for p

SAR = (1-pow((1-p_sel),(7/gamma)));

//For the time period during which the household transmission study was conducted, calculate

//the ratio for the overall number of potentially-infectious contacts per day to the number

//among members of the same household. This ratio is multiplied against the sampled value

//for the mean number of household contacts to estimate the average number of susceptible

//contacts experienced by each infectious individual in an entirely susceptible population.

//Here, we assume that all contacts are close in nature (i.e., similar to within-household

//contacts), which leads to the assumption that b represents the daily cumulative probability

//of infection associated with n potentially infectious contacts with probability of transmission

//of p.

n = mn_hh_size*(q_sel*mn_hh_size+(log(1-b_sel) / log(1 - p_sel)))/(q_sel*mn_hh_size);

//R0 is calculated below, under the assumption detailed a few lines up.

//We introduce a temporal forcing function, where the wavelength of the cosine is fixed at

// 2*3.1415 / 23 weeks (defined in the model calibration component of the code. The

//timeline is shifted by 64 weeks the week of March 3, 2020 is defined as time 0. The code

//also allows for a shifting of the cosine wave, using the delay input parameter, but this was set

//to 0 for Madagascar (also defined in the model calibration component of the code).

slp = n*SAR;

Re = slp*2*(0.5*cos((t-64-delay)*wvl)+0.5);

//Define force of infection (foi)

foi = Re*gamma*I/(S+I+R);

//Define rates for movement of individuals in and out of disease states

rate[0] = foi; //force of infection leaving the S state (stochastic)

rate[1] = mu; //natural death/migration rate leaving the S state (set to 0 by assumption)

rate[5] = gamma; // loss of infectious rate (set to 10.4 days / 7 days per week by assumption)

rate[6] = mu; // natural death/migration rate leaving the I state (set to 0 by assumption)

rate[7] = foi*delta; // force of infection among previously infected individuals (stochastic)

rate[8] = mu; // natural death/migration rate leaving the R state (set to 0 by assumption)

// transitions between classes/states

reulermultinom(2,S,&rate[0],dt,&trans[0]);

reulermultinom(2,I,&rate[5],dt,&trans[5]);

reulermultinom(3,R,&rate[7],dt,&trans[7]);

//The population is assumed to be stable in size, so the number of births is equal to the total

//number of deaths/out-migrations (set to 0 by assumption)

births = trans[1]+trans[6]+trans[8];

S += births - trans[0] - trans[1];

I += trans[0] + trans[7] - trans[5] - trans[6];

R += trans[5] - trans[7] - trans[8];

C = trans[0]+trans[7]; // incident infections

A += R; //Tracking state that accumulates the number of individuals who are in the R state

//between observation times. This variable is reset to 0 at each observation time.

Z += 1; //Tracking state that accumulates the number of weeks that occur between observation

//times. A/Z provides an estimate of the mean number of individuals in the R state

//during the period between observation times. This variable is reset to 0 at each

//observation time.

")

##The following procedure defines the Measurement model linking the SIR model to the

##observed data. This code allows for two potential assumptions regarding the distributions of

##Y1 and Gobs1: negative binomial or Poisson (i.e., setting psi’s equal to 0). We chose the

##latter.

dmeas2 <- Csnippet("

double rho,psi;

rho = rho1;

psi = psi1;

double m4, m6,m4_var;

double m1 = rho*C;

double v1 = m1*(1.0-rho+psi*psi*m1);

double tol = 1.0e-18;

//For the retrospective surveillance period, the expected number of individuals in the R state

//for the period between observations was defined as the observed proportion of SETA

//archived specimens that were anti-SARS-CoV-2 IgG positive multiplied by the population size.

//The observed proportion of SETA archived specimens that were anti-SARS-CoV-2 IgG positive

//was assumed to follow a binomial distribution.

double m3 = Gobs1/(Gobs1+N1)*(S+I+R);

double m3_var = (Gobs1/(Gobs1+N1))*(1-Gobs1/(Gobs1+N1))*(S+I+R);

double m5 = A/Z; //The average R for the period of time since last observation.

//Set non-integer values to the nearest integer value.

if (m3 > 0.0) {

m4 = nearbyint(m3);

} else {

m4 = 0.0;

}

if (m3_var > 0.0) {

m4_var = nearbyint(m3_var);

} else {

m4_var = 0.0;

}

if (m5 > 0.0) {

m6 = nearbyint(m5);

} else {

m6 = 0.0;

}

//Define the likelihood contribution of each observation, with those observations during and after week 137 (start

//of prospective surveillance activities) contributing to the likelihood based upon the observed value for Y1 and

//observations between weeks 64 and 136 contributing to the likelihood based upon the values for Gobs1 and N1.

if (t >= 137) {

if (Y1 > 0.0) {

lik = pnorm(Y1+0.5,m1,sqrt(v1)+tol,1,0)-pnorm(Y1-0.5,m1,sqrt(v1)+tol,1,0)+tol;

} else {

lik = pnorm(Y1+0.5,m1,sqrt(v1)+tol,1,0)+tol;

}

} else {

lik = pnorm(m4+0.5,m6,sqrt(m4_var)+tol,1,0)-pnorm(m4-0.5,m6,sqrt(m4_var)+tol,1,0)+tol;

}

if (give_log) lik = log(lik);

")

##The following code defines the measurement model for simulations using the calibrated

##model. We do not need to generate the Gobs1 and N1 values from the simulations, as they

##are directly reflected by the proportion of the population in the R state.

rmeas2 <- Csnippet("

double tol = 1.0e-18;

double m1 = rho1*C;

double v1 = m1*(1.0-rho1+psi1*psi1*m1);

Y1 = rnorm(m1,sqrt(v1)+tol);

if (Y1 > 0.0) {

Y1 = nearbyint(Y1);

} else {

Y1 = 0.0;

}

")

##The following function defines the initial state for the SIR model at time 0, i.e., week 64.

##propI represents the initial proportion of the population who are infectious. We assume that

##only 1 individual was infectious in this population at time 0. This assumption is encoded in

##the model calibration section of the code.

rinit <- Csnippet("

double pop = 220000;

double intI = propI;

double intS = 1.0 - intI;

double m = pop/(intS+intI);

S = nearbyint(m*intS);

I = nearbyint(m*intI);

R = 0;

C = 0;

A = 0;

Z = 1; //initially set to 1 to avoid division by 0

")

##This function builds the code object that will be used to calibrate and simulate the model

##the partrans setting defines transformations to be applied to input parameters prior to

##sampling values for simulation-based inference. This helps to improve the calibration

##algorithms performance.

m1<-pomp(data=dat[dat$time >= 64,!(colnames(dat) %in% c("st","ed"))],t0=64,

time="time",

rprocess=euler(rproc,delta.t=1),

dmeasure=dmeas2,

rmeasure=rmeas2,

rinit=rinit,

partrans=parameter_trans(

log=c("gamma","mu","psi1","delay"),

logit=c("rho1","delta","propI","wvl")

),

obsnames=c("Y1","Gobs1","N1"),

statenames=c("S","I","R","C","A","Z"),

paramnames=c("gamma","rho1","delta","mu","propI","psi1","decay","wvl","delay"),

accumvars = c("C","A","Z")

)

**4b.3. Model calibration**

Note: The following code calibrates the above model in an iterative fashion. A profile likelihood approach is used to estimate the likelihood profile for each parameter at twenty-five equal spaced intervals across a range of values that can change for each iteration. The initial range of values for each parameter is defined by the user and typically includes the range of all plausible values. For subsequent iterations, the possible range of values for each parameter is defined by the maximum and minimum values for the parameter’s values whose profile likelihood falls within the top 50% of all of the values estimated from a LOESS fit to the accumulated calibration runs for all preceding iterations. For each profile likelihood run, we run ten random replicates for each of the twenty-five locations along the current range for the parameter’s values. By random replicate, we mean that though the value for the parameter being profiled remains fixed, we randomly draw values from the allowed range(s) for all other parameters that we are trying to calibrate.

##Start of code

set.seed(123456)

for(x in 1:4) {

crange<-c(2,3) ##define which of the parameters in the ‘ranges’ list we are planning to profile

vind<-235 ##define the model calibration run’s unique identifier

##define whether or not we want to turn on standard error informed smoothing of the LOESS smoothing procedure for defining each likelihood profile. We chose to not use this functionality.

wind<-0

##Define the range of allowed values for each parameter. In the initial part of the code, the user defines the initial

##allowed range. The first vector defines the minimum and the second the maximum for each parameter. Here is

##where some assumptions are encoded.

if (x==1) {

ranges<-rbind(c(gamma=7/10.4,rho1=0.0,delta=0.0,mu=0,propI=1/220000,psi1=0,decay=0.0,wvl=2*pi/23,delay=0),

c(gamma=7/10.4,rho1=1.0,delta=1.0,mu=0,propI=1/220000,psi1=0,decay=0.0,wvl=2*pi/23,delay=0),deparse.level=0)

}

if(x > 1) {

ranges1<-ranges2[1:2,]

for(c in crange) {

if(ranges[1,c]==ranges2[1,c]) {

ranges1[1,c]<-(ranges2[1,c]+0.5*(ranges2[2,c]-ranges2[1,c]))

if((ranges2[1,c]+0.5*(ranges2[2,c]-ranges2[1,c])) < ranges[2,c]*0.5) {

ranges1[2,c]<-ranges[2,c]*0.5

}

if((ranges2[1,c]+0.5*(ranges2[2,c]-ranges2[1,c])) >= ranges[2,c]*0.5) {

ranges1[2,c]<-ranges[2,c]

}

}

if(ranges[2,c]==ranges2[2,c]) {

ranges1[2,c]<-(ranges2[1,c]+0.5*(ranges2[2,c]-ranges2[1,c]))

if((ranges2[1,c]+0.5*(ranges2[2,c]-ranges2[1,c])) > ranges[2,c]*0.5) {

ranges1[1,c]<-ranges[2,c]*0.5

}

if((ranges2[1,c]+0.5*(ranges2[2,c]-ranges2[1,c])) <= ranges[2,c]*0.5) {

ranges1[1,c]<-ranges[1,c]

}

}

if (ranges1[1,c] > ranges1[2,c]) {

ranges1[1:2,c]<-ranges1[2:1,c]

}

}

ranges<-ranges1

rm(ranges1)

}

cn<-colnames(ranges)

##Start the process of defining the input to facilitate the profile likelihood exercise

starts<-c()

rind<-x

minrind<-rep(1,12)

maxrind<-rep(rind,12)

ccnt<-0

ranges2<-c()

##Here we are starting the looping over the parameters to be profiled.

for(c in crange) {

cn_temp <- c(cn[c],cn[-c])

##The rv[] vector controls how the modified iterative filtering algorithm samples each parameter (using the

##index/order listed in ‘ranges’) during the calibration process. Essentially, if rv[] is set to 0, then the algorithm will

##not try to vary the input value for a parameter.

rv=rep(0.02,ncol(ranges))

rv[c]<-0

rv[1]<-0

##rv[2]<-0

##rv[3]<-0

rv[4]<-0

rv[5]<-0

rv[6]<-0

rv[7]<-0

rv[8]<-0

rv[9]<-0

##This builds the set of start values for the 10 replicates runs for each of the 25 locations along the possible range ##of values for the parameter being profiled.

starts <- profile_design(

t1=seq(

from=ranges[1,c],

to=ranges[2,c],

length=25

),

lower=ranges[1,-c],

upper=ranges[2,-c],

nprof=10

)

colnames(starts)<-cn_temp

start_t<-Sys.time()

coefs<-c()

logliks<-c()

##The following loop estimates the likelihood of the data given the model and the starting parameter values

##presented in each row of the ‘starts’ matrix. Since values for the parameter being profiled are being held

##constant (i.e., the modified iterative filtering algorithm does not change the initial input value), the results of this

##loop are the estimated likelihood for each of the ten replicates of the twenty-five different values for the

##parameter being profiled.

for(i in 1:nrow(starts)) {

start_t_step<-Sys.time()

mif<-try(mif2(m1,params=starts[i,],

Nmif=10,

Np=50,

rw.sd=rw_sd(gamma=rv[1],

rho1=ifelse(time < 137,0,rv[2]),

delta=rv[3],

mu=rv[4],

propI=rv[5],

theta=rv[6],

ehh=rv[7],

psi1=ifelse(time < 137,0,rv[8]),

decay = rv[9],

wvl = rv[10],

delay = rv[11]),

cooling.type = "geometric",cooling.fraction.50=0.5,verbose=F))

coefs<-rbind(coefs,try(coef(mif)),deparse.level=0)

try(mif |> pfilter(Nrep=5,verbose=F) |> logLik() |> logmeanexp(se=TRUE,ess=TRUE)) -> tloglik

logliks<-rbind(logliks,tloglik,deparse.level=0)

rm(mif)

rm(tloglik)

print(paste(cn[c],": ",i,": ",Sys.time()-start_t_step,": ",Sys.time()-start_t))

}

##Writing output to file

write.csv(coefs,paste("profile_",cn[c],"_coef_v",vind,"_",rind,".csv",sep=""))

write.csv(logliks,paste("profile_",cn[c],"_loglik_v",vind,"_",rind,".csv",sep=""))

}

##Fit LOESS curve to all profile likelihood estimates generated up to this point (across the current and preceding

##iterations) for parameter currently being profiled.

minrind<-rep(1,12)

maxrind<-rep(rind,12)

ranges2<-c()

ccnt<-0

for(c in crange ) {

ccnt<-ccnt+1

cn_temp <- c(cn[c],cn[-c],"loglik","loglik.se","loglik.ess")

t0<-c()

for(i in minrind[ccnt]:maxrind[ccnt]) {

if(file.exists(paste("profile_",cn[c],"_coef_v",vind,"_",i,".csv",sep=""))) {

t1<-read.csv(paste("profile_",cn[c],"_coef_v",vind,"_",i,".csv",sep=""))

t2<-read.csv(paste("profile_",cn[c],"_loglik_v",vind,"_",i,".csv",sep=""))

t1a<-which(grepl("Error",t1[,2],fixed=T)==F & grepl("Error",t2[,2],fixed=T)==F)

t1<-t1[t1a,]

t2<-t2[t1a,]

t1<-matrix(as.numeric(unlist(t1)),nrow=nrow(t1))

t2<-matrix(as.numeric(unlist(t2)),nrow=nrow(t2))

t0<-as.data.frame(rbind(t0,cbind(t1[,-1],t2[,-1]),deparse.level=0))

}

}

colnames(t0)<-cn_temp

t0<-t0[apply(t0,1,FUN=function(x) sum(as.numeric(grepl("Error",x,fixed=T)))) == 0,]

##If using standard error informed weighting, then these weights are defined here.

if(wind==1) {

wts<-t0$loglik.ess

} else {

wts<-rep(1,nrow(t0))

}

##The LOESS fit for the likelihood profile, including definition of likelihood ratio based 95% confidence limits

m1_x<-seq(min(t0[,1]),max(t0[,1]),length.out=1.0e6)

m1m <- loess(t0$loglik~t0[,1],model=T,weights=wts)

m1p<-predict(m1m,newdata=m1_x)

m1_xs<-m1_x[m1p >= (max(m1p)-5)]

m1_xs2<-m1_x[m1p >= (max(m1p)-2)]

m1ps<-m1p[m1p >= (max(m1p)-5)]

m1ps2<-m1p[m1p >= (max(m1p)-2)]

m1_xs2<-m1_x[m1p >= (max(m1p) - 0.1*(max(m1p)-min(m1p)))]

ll<-min(m1_xs2)

ul<-max(m1_xs2)

m1_xs2<-m1_x[m1p >= (max(m1p)-2)]

mle<-m1_xs2[m1ps2==max(m1ps2)]

mle_ll<-max(m1ps2)

ranges2<-cbind(ranges2,c(ll,ul,mle))

}

##End of the parameter calibration loop for an iteration

ranges2<-cbind(rep(ranges[1,1],nrow(ranges2)),ranges2[,1:2],rep(ranges[1,4],nrow(ranges2)),rep(ranges[1,5],nrow(ranges2)),rep(ranges[1,6],nrow(ranges2)),rep(ranges[1,7],nrow(ranges2)),rep(ranges[1,8],nrow(ranges2)),rep(ranges[1,9],nrow(ranges2)))

colnames(ranges2)<-cn

##Simulate and plot epidemics using the current model calibration

t3<-dat$time[dat$time>=64]

ranges3<-ranges2[3,]

simout<-c()

i<-0

plot(dat$time,dat$Y1,ylim=c(1,100),xlim=c(137,max(dat$time)),type="p",col=0,xlab="Study Week",ylab="Number of laboratory-confirmed symptomatic SARS-CoV-2 cases")

while(i < 1000) {

t1<-simulate(m1,nsim=1,params=ranges3,format="data.frame",verbose=F,times=dat$time[dat$time >= 64])

if(t1$I[nrow(t1)] >= 0)

{

i<-i+1

if(i %in% c(10*seq(1,100))) {

lines(t1$time,t1$Y1, col="gray")

}

simout<-rbind(simout,cbind(rep(i,nrow(t1)),t1),deparse.level=0)

}

t3<-cbind(t3,t1$A/t1$Z/220000)

}

t2<-aggregate(simout$Y1,by=list(simout$time),mean)

lines(t2$Group.1,t2$x,col="red")

points(dat$time,dat$Y1,ylim=c(1,100),col=1,pch=20)

cbind(t3[t3[,1] < 137,1],rowSums(t3[t3[,1] < 137,2:ncol(t3)])/ncol(t3))

unique(simout[simout[,2]==150 & simout$Y1 > 50,1])

}

## End of the iteration loops

**Section 5. R code and data for implementing the exploratory test-negative case control study for vaccination in Burkina Faso, with matching on age group () and month of enrollment into the prospective surveillance system**

**5a. Input data elements:** formatted as four input data matrices containing the number of individuals (counts for each combination of vaccination and case/control status) falling within strata defined by two matching criteria: age group (0-4, 5-9, 10-14, 15-19, 20-24, and 25-54 years, as well as 55 years and older) and visit month (yyyy-mm).

t1.case <-

|  |  | **Age group** | | | | | | |
| --- | --- | --- | --- | --- | --- | --- | --- | --- |
| **Enrollment Month (yyyy-mm)** | **Vaccination Status (1=Yes,0=No)** | **0-4 years** | **5-9 years** | **10-14 years** | **15-19 years** | **20-24 years** | **25-54 years** | **55 years and older** |
| 2021-06 | 0 | 0 | 0 | 0 | 0 | 0 | 1 | 0 |
| 2021-08 | 0 | 0 | 0 | 0 | 0 | 0 | 1 | 0 |
| 2021-09 | 0 | 0 | 0 | 0 | 1 | 0 | 1 | 0 |
| 2021-10 | 0 | 0 | 0 | 0 | 1 | 1 | 2 | 0 |
| 2021-11 | 0 | 0 | 0 | 0 | 1 | 1 | 4 | 0 |
| 2021-12 | 0 | 3 | 0 | 2 | 3 | 20 | 89 | 15 |
| 2021-12 | 1 | 0 | 0 | 0 | 0 | 1 | 15 | 3 |
| 2022-01 | 0 | 2 | 0 | 1 | 0 | 0 | 14 | 2 |
| 2022-01 | 1 | 0 | 0 | 0 | 0 | 0 | 4 | 0 |
| 2022-02 | 1 | 0 | 0 | 0 | 0 | 0 | 1 | 0 |
| 2022-05 | 0 | 0 | 0 | 0 | 0 | 1 | 4 | 0 |
| 2022-05 | 1 | 0 | 0 | 0 | 0 | 0 | 1 | 0 |
| 2022-06 | 0 | 1 | 0 | 0 | 0 | 0 | 1 | 0 |
| 2022-07 | 0 | 0 | 0 | 1 | 0 | 0 | 0 | 0 |
| 2022-08 | 0 | 2 | 1 | 0 | 0 | 2 | 2 | 0 |
| 2022-09 | 0 | 3 | 1 | 0 | 0 | 2 | 8 | 0 |

t1.control <-

|  |  | **Age group** | | | | | | |
| --- | --- | --- | --- | --- | --- | --- | --- | --- |
| **Enrollment Month (yyyy-mm)** | **Vaccination Status (1=Yes,0=No)** | **0-4 years** | **5-9 years** | **10-14 years** | **15-19 years** | **20-24 years** | **25-54 years** | **55 years and older** |
| 2021-06 | 0 | 0 | 0 | 0 | 0 | 0 | 1 | 0 |
| 2021-08 | 0 | 0 | 0 | 0 | 0 | 0 | 8 | 0 |
| 2021-09 | 0 | 0 | 0 | 0 | 1 | 0 | 3 | 0 |
| 2021-10 | 0 | 0 | 0 | 0 | 1 | 4 | 4 | 0 |
| 2021-11 | 0 | 0 | 0 | 0 | 4 | 1 | 7 | 0 |
| 2021-12 | 0 | 15 | 0 | 16 | 21 | 45 | 279 | 39 |
| 2021-12 | 1 | 0 | 0 | 0 | 0 | 3 | 36 | 8 |
| 2022-01 | 0 | 28 | 0 | 5 | 0 | 0 | 92 | 13 |
| 2022-01 | 1 | 0 | 0 | 1 | 0 | 0 | 21 | 9 |
| 2022-02 | 0 | 0 | 0 | 0 | 0 | 0 | 13 | 0 |
| 2022-02 | 1 | 0 | 0 | 0 | 0 | 0 | 2 | 0 |
| 2022-05 | 0 | 0 | 0 | 0 | 0 | 4 | 28 | 0 |
| 2022-05 | 1 | 0 | 0 | 0 | 0 | 0 | 2 | 0 |
| 2022-06 | 0 | 70 | 0 | 0 | 0 | 0 | 28 | 0 |
| 2022-06 | 1 | 0 | 0 | 0 | 0 | 0 | 2 | 0 |
| 2022-07 | 0 | 0 | 0 | 3 | 0 | 0 | 0 | 0 |
| 2022-08 | 0 | 52 | 7 | 0 | 0 | 27 | 37 | 0 |
| 2022-09 | 0 | 45 | 2 | 0 | 0 | 23 | 46 | 0 |
| 2022-09 | 1 | 0 | 0 | 0 | 0 | 0 | 5 | 0 |

**5b. Code for running generating 1000 test-negative studies based upon the data inputs**

results<-c()

for(r in 1:1000){

tempdata<-c()

clusterid<-0

iid<-0

for(c in 3:ncol(t1.case)) {

for(d in 1:nrow(t1.case)) {

if(t1.case[d,c] > 0) {

##temp.case<-t1.case[t1.case$agegrp==strata$agegrp[s] & t1.case$visitcat==strata$visitcat[s],]

##temp.control<-t1.control[t1.control$agegrp==strata$agegrp[s] & t1.control$visitcat==strata$visitcat[s],]

for(i in 1:t1.case[d,c]) {

clusterid<-clusterid+1

iid<-iid+1

control_unexp <- ifelse(length(t1.control[t1.control$visitcat==t1.case$visitcat[d] & t1.control$vax==0,c]) > 0,t1.control[t1.control$visitcat==t1.case$visitcat[d] & t1.control$vax==0,c]/sum(t1.control[t1.control$visitcat==t1.case$visitcat[d],c]),0)

control_exp <- 1 - control_unexp

##control_order<-matrix(cbind(seq(1,nrow(temp.control)),runif(nrow(temp.control))),nrow=nrow(temp.control))

##control_order<-matrix(control_order[order(control_order[,2]),],nrow=nrow(temp.control))

tempdata<-rbind(tempdata,c(iid,c-2,t1.case$visitcat[d],t1.case$vax[d],1,clusterid),deparse.level=0)

for(j in 1:min(sum(t1.control[t1.control$visitcat==t1.case$visitcat[d],c]),max_control_ratio,na.rm=T)) {

iid<-iid+1

tempdata<-rbind(tempdata,c(iid,c-2,t1.case$visitcat[d],as.numeric(runif(1,0,1) < control_exp),0,clusterid),deparse.level=0)

}

}

}

}

}

##tempdata<-matrix(tempdata,nrow=nrow(tempdata))

colnames(tempdata)<-c("iid","agegrp","visitcat","vax","case","clusterid")

tm<-clogistic(as.numeric(case) ~ as.numeric(vax),strata=clusterid,data=as.data.frame(tempdata))

results<-rbind(results,c(r,tm$coefficients,tm$var),deparse.level=0)

}

exp(mean(results[,2]))

exp(mean(results[,2])-1.96*sqrt(var(results[,2])+mean(results[,3])))

exp(mean(results[,2])+1.96*sqrt(var(results[,2])+mean(results[,3])))
